# Induction of Neuronal Morphology in the 661W Cone Photoreceptor Cell Line with Staurosporine

**DOI:** 10.1371/journal.pone.0145270

**Published:** 2015-12-18

**Authors:** Alex F. Thompson, Megan E. Crowe, Christopher J. Lieven, Leonard A. Levin

**Affiliations:** 1 Department of Ophthalmology and Visual Sciences, University of Wisconsin School of Medicine and Public Health, Madison, Wisconsin, United States of America; 2 Department of Ophthalmology, McGill University, Montreal, Quebec, Canada; Dalhousie University, CANADA

## Abstract

**Purpose:**

RGC-5 cells undergo differentiation into a neuronal phenotype with low concentrations of staurosporine. Although the RGC-5 cell line was initially thought to be of retinal ganglion cell origin, recent evidence suggests that the RGC-5 line could have been the result of contamination with 661W mouse cone photoreceptor cells. This raised the possibility that a cone photoreceptor cell line could be multipotent and could be differentiated to a neuronal phenotype.

**Methods:**

661W and RGC-5 cells, non-neuronal retinal astrocytes, retinal endothelial cells, retinal pericytes, M21 melanoma cells, K562 chronic myelogenous leukemia cells, and Daudi Burkitt lymphoma cells, were differentiated with staurosporine. The resulting morphology was quantitated using NeuronJ with respect to neurite counts and topology.

**Results:**

Treatment with staurosporine induced similar-appearing morphological differentiation in both 661W and RGC-5 cells. The following measures were not significantly different between 661W and RGC-5 cells: number of neurites per cell, total neurite field length, number of neurite branch points, and cell viability. Neuronal-like differentiation was not observed in the other cell lines tested.

**Conclusions:**

661W and RGC-5 cells have virtually identical and distinctive morphology when differentiated with low concentrations of staurosporine. This result demonstrates that a retinal neuronal precursor cell with cone photoreceptor lineage can be differentiated to express a neuronal morphology.

## Introduction

The RGC-5 cell line was produced as a continuously proliferating model of rat retinal ganglion cells (RGCs). A characteristic feature of RGC-5 cells is that they develop a neuronal morphology when treated with the broad-spectrum protein kinase inhibitor staurosporine [1–9]. While untreated RGC-5 cells are flat and polygonal, cells treated with staurosporine do not divide and instead develop processes and round somas [1]. Treatment with staurosporine also causes RGC-5 cells to express ion channels, as evidenced by electrophysiology [1], and induces neurite expression of microtubule-associated protein 2, tau, and growth-associated protein 43 [2]. The presence of these markers indicates that RGC-5 cells can develop axon-like and dendrite-like processes when differentiated, features suggestive of a terminally differentiated neuron.

Recent studies have questioned the origin of the RGC-5 cell line [8, 10]. We and others have used mitochondrial DNA sequencing to show that RGC-5 cells are of mouse and not rat origin [10]. Studies inconsistently show expression of the RGC marker Thy-1 in RGC-5 cells [8, 10, 11], although differentiation increases Thy-1 levels in several laboratories [1, 9, 12]. This was in contrast to earlier work, which reported RGC-5 cells as consistently Thy-1 positive [13]. It has been speculated that RGC-5 cells could have been unintentionally contaminated by 661W cells, a mouse photoreceptor cell line that was being studied in the same laboratory where RGC-5 cells were produced [10, 14–21]. The 661W cell line was derived from retinal tumors induced in transgenic mice by infecting embryos with a construct containing SV40 large T-antigen under the control of the human interphotoreceptor retinoid binding protein (IRBP) gene [22]. Proteins expressed by 661W cells include cone pigments, transducin, and cone arrestin, antigens consistent with a cone photoreceptor lineage [23].

If the RGC-5 cell line were actually derived from contaminating 661W cone photoreceptor-like cells, then the staurosporine-induced expression of dendritic and axonal markers in RGC-5 cells would imply cells of a cone lineage could be induced to display a neuronal phenotype. To directly test this hypothesis, 661W and RGC-5 cells were treated with low concentrations of staurosporine and the resulting neuronal morphology was quantitatively assessed. In order to see whether the neuronal morphology was a general phenomena associated with staurosporine exposure, the same procedure was followed for several non-neuronal cell lines.

## Methods

### Materials

Staurosporine (isolated from *Streptomyces staurosporeus*) was obtained from Alexis Biochemical (San Diego, CA). Fetal bovine serum was obtained from Gemini Bio-products (West Sacramento, CA). Other cell culture reagents were obtained from Mediatech (Herndon, VA) unless noted. The commercial or laboratory sources and literature reference to their first description for all cell lines used in this study are detailed in Table 1. Paraformaldehyde (16% solution) was obtained from Electron Microscopy Sciences (Hatfield, PA).

**Table 1:** Sources and literature references for all cell lines used in the study.

Cell line	Description and Literature References	Source
RGC-5	Originally described as an immortalized cell line derived from rat retinal ganglion cells [13], Recently shown to be of mouse origin instead of rat [10, 20]	Dr. Neeraj Agarwal of the Utah School of Medicine
661W	Immortalized mouse photoreceptor cell line [22]	Dr. Muayyad Al-Ubaidi of the University of Oklahoma Health Sciences Center
Retinal astrocytes	Astrocytes isolated from wild-type and transgenic Immortomice by collagenase digestion of the retina [30]	Dr. Nader Sheibani of the University of Wisconsin
Retinal endothelial cells	Isolated from wild type or transgenic-immortomouse by collagenase digestion of retina and affinity purification using magnetic beads coated with platelet/endothelial cell adhesion molecule-1 [31]	Dr. Nader Sheibani
Retinal Pericytes	Isolated from mouse retinas by collagenase digestion [32]	Dr. Nader Sheibani
M21 melanoma	Melanoma cell line derived from human metastatic axillary node [33]	Dr. Paul Sondel of the University of Wisconsin-Madison
K562 chronic myelogenous leukemia	Lymphoblast derived from human bone marrow of chronic myelogenous leukemia patient [34]	Dr. Paul Sondel
Daudi Burkitt lymphoma	Lymphoblast derived from peripheral blood of Human Burkitt’s lymphoma patient [35]	Dr. Paul Sondel

### Cell culture

661W and RGC-5 cells were cultured in Dulbecco's modified Eagle's medium (DMEM) with 1 g/L glucose and l-glutamine, supplemented with 10% fetal bovine serum (FBS), 100 U/mL penicillin, and 100 μg/mL streptomycin. Cells were incubated at 37°C in humidified 5% CO_2_. Standard line-specific culture conditions were used for non-neuronal control cells.

### Differentiation with staurosporine

Twenty-four hours before treatment with staurosporine, cells were plated onto 12 mm round glass cover slips coated with 0.01% poly-L-lysine in 24-well plates at a density of approximately 4,000 cells/cm^2^ in 450 μL medium. Stock staurosporine in DMSO was diluted in medium and added to cultures to final concentrations of 0 nM, 100 nM, 316 nM, or 1 μM. After 24 hours of treatment, cells were fixed in 4% paraformaldehyde for 10 min, and cover slips mounted on glass slides and sealed. All conditions were performed in triplicate.

### Cell morphology

Digital photomicrographs of cells on mounted cover slips were taken at 400X total magnification on a Zeiss Axiophot microscope with DIC optics and stored as TIFF images. Photomicrographs were analyzed and the number of primary neurites per cell counted. Primary neurites were classified as projections that originated at the cell soma and were at least as long as the soma was long or wide. Neurite counts were averaged for each condition. The NeuronJ plug-in for NIH ImageJ software was used to assess neurite length and branching. Neurites were traced, and labeled as primary, secondary, tertiary, quaternary, or quinary. The number of points at which neurites branched was counted for each cell. Length measurement from individual neurites were converted to microns, and the value for total neurite field length for each cell was calculated.

### Cell viability

After differentiation with staurosporine, growth media was removed and cells treated with 10 μg/mL calcein-AM in phosphate-buffered saline and incubated for 30 minutes at room temperature in the dark. Digital photomicrographs of the wells were taken at a total magnification of 200X. ImageJ software was used to tabulate the number of viable green-fluorescing cells.

### Statistics

Significance testing was performed with Student’s t to compare continuous values between groups.

## Results

### Staurosporine treatment induces neuronal-like differentiation in 661W and RGC-5 cells

**Fig 1:**
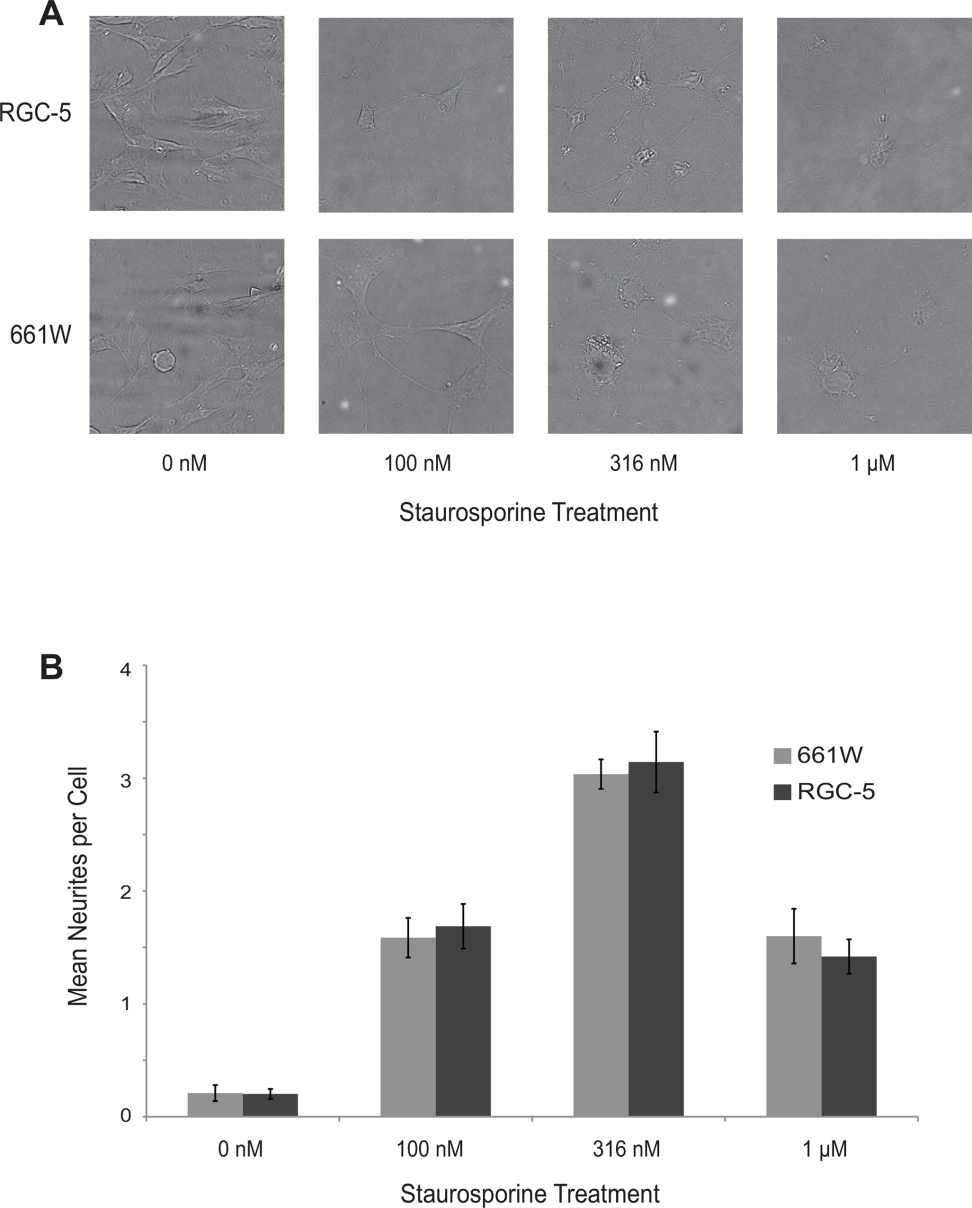
661W and RGC-5 cells differentiated with staurosporine have similar numbers of induced neurites. Cells were incubated in the indicated concentrations of staurosporine for 24 hr, fixed, photographed, and the number of neurites longer than twice the cell soma counted. **(A)** Microphotographs of sample fields of treated cells. **(B)** Mean ± SEM number of neurites per cell. Comparisons between 661W and RGC-5 were not significantly different at every concentration studied.

Treatment with staurosporine for 24 hr induced similar-appearing morphological differentiation in 661W and RGC-5 cells. Cells developed round, raised somas and extended neurites (Fig 1). Untreated 661W and RGC-5 cells extended 0.21 ± 0.07 and 0.20 ± 0.04 neurites per cell, respectively. Treatment with 100 nM, 316 nM, or 1 μM staurosporine significantly increased the number of neurites observed per cell for both cell types (p < 0.00001 for all comparisons to untreated cells). The greatest number of neurites per cell was observed with 316 nM staurosporine. 661W and RGC-5 cells treated with this concentration expressed an average of 3.04 ± 0.13 and 3.14 ± 0.27 primary neurites per cell, respectively. The number of neurites extended after staurosporine treatment did not significantly differ between 661W and RGC-5 cells at any concentration tested (Fig 1).

### Differentiated 661W and RGC-5 cells have quantitative morphological similarities

**Fig 2:**
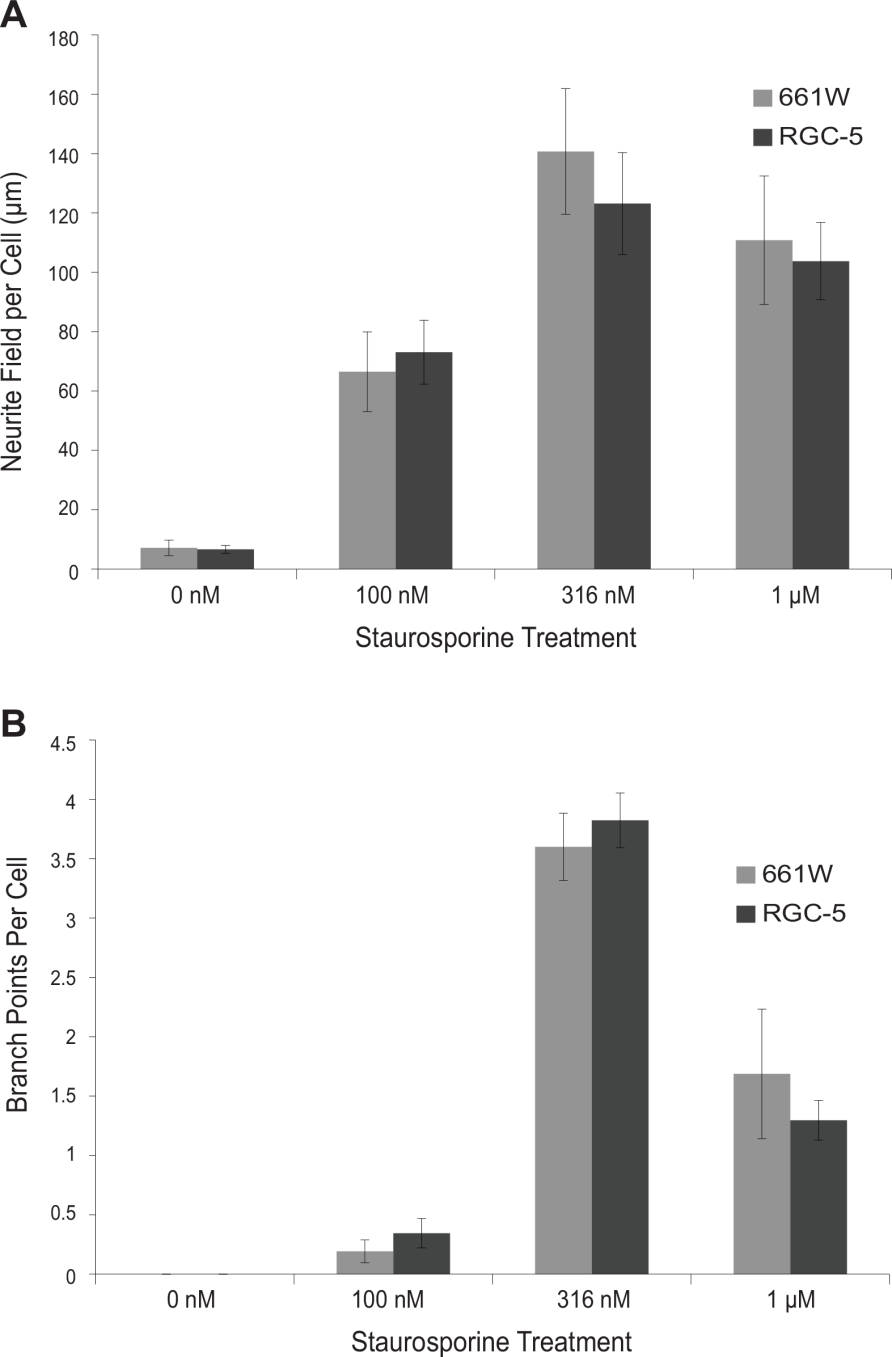
661W and RGC-5 cells differentiated with staurosporine have similar total neurite lengths and numbers of neurite branch points. Cells were incubated in the indicated concentrations of staurosporine for 24 hr, fixed, photographed, and neurites traced with NeuronJ. **(A)** Mean ± SEM of the total neurite length per cell. **(B)** Mean ± SEM of the number of neurite branch points per cell. Comparisons between 661W and RGC-5 were not significantly different at every concentration studied.

To more precisely quantify the nature of the differentiation response seen in 661W and RGC-5 cells following staurosporine treatment, the NeuronJ plugin for ImageJ was used to trace and label neurites. Neurites were measured and classified as primary, secondary, quaternary, or quinary. This data was used to assess total neurite field length and number of neurite branch points, a measure of neurite field complexity, for each condition. For both of these measures, the differentiation response of 661W and RGC-5 cells did not significantly differ. For both cell types, maximum neurite field length and maximum neurite branch points were observed after treatment with 316 nM staurosporine. In 661W cells, 316 nM staurosporine treatment resulted in 140.7 ± 21.2 μm average neurite field length and 3.60 ± 0.28 branch points per cell. Similarly, 316 nM staurosporine treatment of RGC-5 cells resulted in an average total neurite field per cell of 123.2 ± 17.2 μm and an average of 3.82 ± 0.23 branch points per cell (Fig 2).

### Effect of staurosporine on cell viability

**Fig 3:**
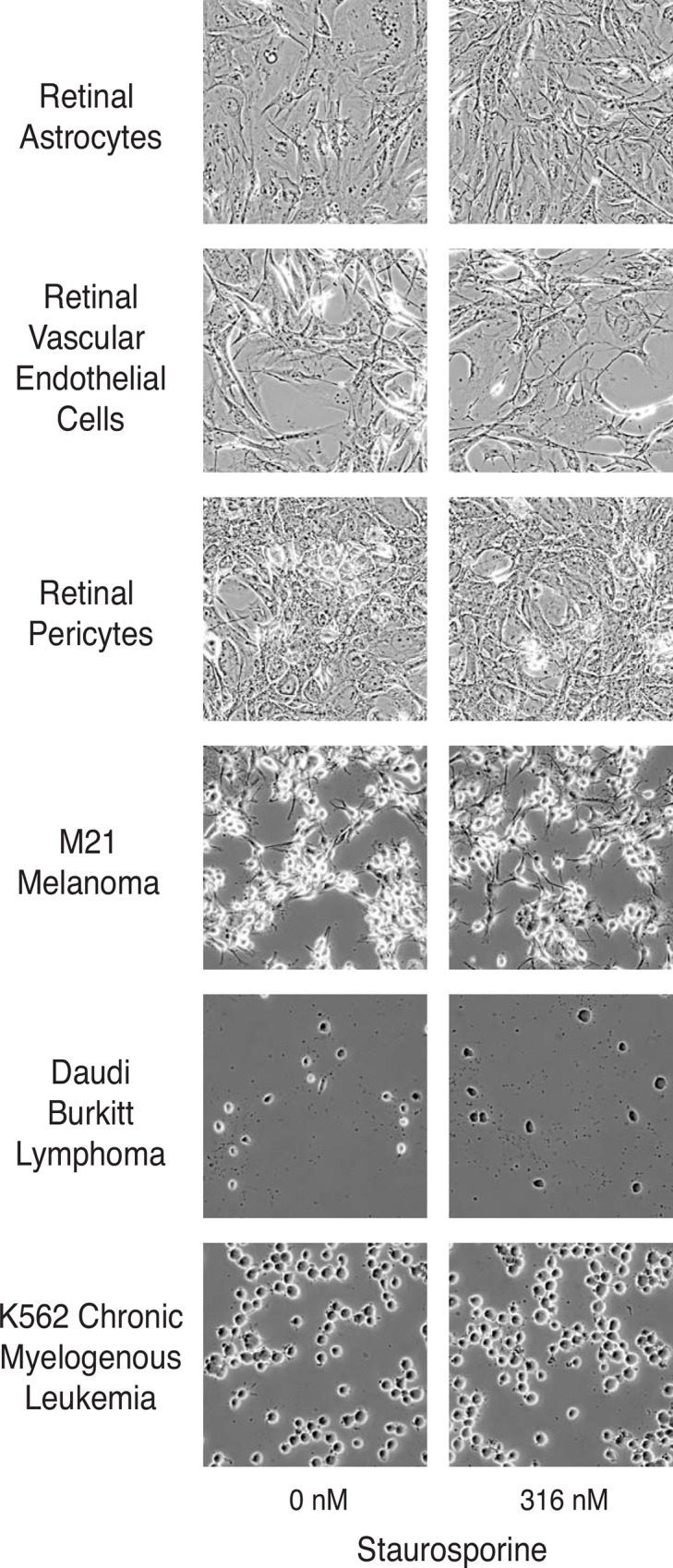
Staurosporine does not induce neurite formation in other retinal and non-retinal cell lines. Cells were incubated with vehicle control or staurosporine (316 nM) for 24 hr, fixed, and photographed. Neurites were not induced in the cell lines studied.

Staurosporine is a known inducer of apoptosis in numerous cell lines (Bertrand 1994), although RGC-5 cells are less sensitive to it [1]. To test if this were the case in 661W cells, the effect of staurosporine treatment on 661W and RGC-5 cell viability was compared. Treatment of 661W cells with 1 μM staurosporine, the highest concentration used in differentiation studies, results in a small but statistically significant decrease in number of viable cells, as measured by calcein-AM staining. In wells treated with vehicle control, 96.9 ± 0.3% of untreated RGC-5 cells were viable after 24 hours, compared to 92.5 ± 0.6% of cells treated with 1 μM staurosporine (p < 0.01). An identical response was seen in RGC-5 cells, with 97.7 ± 0.3% of 661W cells viable after 24 hours, compared to 92.7 ± 1.5% of cells treated with 1 μM staurosporine (p < 0.001) (Fig 3).

### Response of other cell lines to staurosporine

The nearly identical differentiation response of 661W and RGC-5 cells when treated with staurosporine could could reflect a generic response of retinal and other cells to low concentrations of this broad-spectrum kinase inhibitor. To test this hypothesis, cell lines of diverse origin (including three other retinal cell lines) were treated with 316 nM staurosporine, the concentration that most effectively induced differentiation in 661W and RGC-5 cells. Treatment of retinal pericytes, retinal endothelial cells, or retinal astrocytes with staurosporine did not result in morphological differentiation. To test non-retinal continuous cell lines, the response of the M21 melanoma, K562 chronic myelogenous leukemia, and Daudi Burkitt lymphoma cells to staurosporine was tested. None of these cell lines exhibited morphological differentiation after treatment with 316 nM staurosporine (Fig 3).

## Discussion

The 661W cone photoreceptor cell line underwent differentiation to a neuronal-like morphology when exposed to low concentrations of staurosporine. The responses of 661W and RGC-5 cells to treatment with low-dose staurosporine were strikingly similar. A dose-response relationship between staurosporine and degree of differentiation was seen with both cell types, with both 661W and RGC-5 cells extending approximately the same numbers of neurites when treated with the same concentrations of staurosporine. Optimal differentiation of both cell types occurred with 316 nM staurosporine treatment. Staurosporine treatment did not induce significant apoptosis in either of the cell lines observed. Instead, cells from both lines stopped dividing and displayed a neuronal phenotype after staurosporine treatment.

This identical dose-response of differentiation to staurosporine seems to be distinctive for 661W and RGC-5 cells. The induction of differentiation by staurosporine is unlikely to be a universal cellular process because staurosporine did not induce a neuronal morphology in retinal pericytes, retinal endothelial cells, or retinal astrocytes, nor in melanoma, lymphoma, or leukemia cells. A past study [6], in which the PC12 and PC6-3 neuronal precursor cell lines were differentiated with staurosporine, showed less neurite production and other neuronal features that did not match the dose-response relationship seen with 661W and RGC-5 cells. In that study, PC-12 cells showed greatest neuronal differentiation at 100 nM staurosporine, with 1.7 ± 0.3 primary neurites per cell. PC6-3 cells showed greatest neuronal differentiation at 1 μM staurosporine, with 1.7 ± 0.2 primary neurites per cell. This is different from the present study, in which 661W and RGC-5 cells developed an average of 3.0 ± 0.1 and 3.1 ± 0.3 primary neurites per cell, respectively, when exposed to 316 nM staurosporine, the concentration which was optimal for differentiation. [17]. It should be also noted that that study and the present study only assessed morphological features of neuronal differentiation, and a more extensive study of cell-surface markers and electrophysiological activity would be needed to confirm a neuronal phenotype, as was done previously [1].

The nature of the neuronal differentiation signal for 661W cells is unclear. Sheedlo et al showed that 661W cells exposed to fibroblast growth factor-2 underwent some morphological changes similar to differentiation into neuronal morphology, including extension of processes and aggregation into small clusters [24]. Staurosporine is an extremely broad-spectrum kinase inhibitor, with apoptosis-inducing effects as well, and the mechanism by which it results in a differentiated phenotype is still unclear [1, 6]. The fact that similar neuronal differentiation occurs with 7-hydroxystaurosporine (UCN-01) but not other structurally related compounds (e.g. 9,12-epoxy-1H- diindolo[1,2,3-fg:30, 20, 10 -kl]pyrrolo[3,4-i][1,6]benzodiazocine-10-carboxylic acid, 2,3,9,10,11,12-hexahy- dro-10-hydroxy-9-methyl-1-oxo-, methyl ester, (9S,10R,12R)-(K252a), (5R,6S,8S)-6-hydroxy-5-methyl- 13-oxo-6,7,8,13,14,15-hexahydro-5H-16-oxa-4b,8a,14-triaza-5,8-methanodibenzo[b,h]cycloo- cta[jkl]cyclopenta[e]-as-indacene-6-carboxylic acid (K252b), staurosporine aglycone (K252c), or 40-N-benzoylstaurosporine (PKC-412)) is an argument that one of the critical factors is the presence of a basic amine adjacent to an accessible methoxy group at the 3’ carbon [6]. This does not explain the relative cellular specificity of the differentiating signal.

Schultheiss et al [25] found that 50nM or higher concentrations of staurosporine induced high levels of apoptosis in RGC-5 cells, a concentration less than was used in the present study, where significant apoptosis was not found in either 661W or RGC-5 cells at 316 nM. However, the Schultheiss study described RGC-5 tissue culture using 5% FBS, which is half the normal concentration (10% FBS) needed for RGC-5 long-term survival [8, 10, 13, 26, 27]. It is therefore likely that inadequate serum concentrations contributed to an increased sensitivity to the apoptotic effects of staurosporine in these cells. It should also be recognized that the similarity of differentiation of 661W and RGC-5 cells with staurosporine does not imply that the cell lines are the same, as also suggested by a variety of minor differences between the two lines [20, 21].

In conclusion, these data demonstrate that a retinal neuronal precursor cell differentiated along a cone photoreceptor lineage can be redifferentiated along a classic neuronal lineage with respect to morphology, and in the case of RGC-5 cells, to express axons and dendrites [2]. Cones and RGCs are both early-appearing cells in the retinal developmental lineage [28], and the ability to switch from one cell lineage to the other could be explained by some common differentiation pathways. In addition, the remarkably similar differentiation responses to low concentrations of staurosporine also support the evidence [8] that RGC-5 cells are related (but not necessarily identical [21]) to 661W cells, a conclusion also supported by the fact that RGC-5 cells express cone opsins [8, 27]. The fact that a photoreceptor precursor cell line can shift to a neuronal morphology may add a new strategy for retinal ganglion cell replacement for regenerative purposes [29].

## Supporting Information

S1 File Supporting information for experiments in the study. (PDF)
