# Supplementary material for: Induction of Neuronal Morphology in the 661W Cone Photoreceptor Cell Line with Staurosporine
Source: PLoS One. 2015 Dec 18;10(12):e0145270. doi: 10.1371/journal.pone.0145270 (PMC4684327; doi:10.1371/journal.pone.0145270)
Supplement: S1 File — (PDF) [file pone.0145270.s001.pdf]

**1. Mouse mitochondrial DNA is 82.5% identical to rat**

Cell Biology Unit Web Server.
Bioinformatics:

# align results

**Please cite:** *Pearson, W.R., Wood, T., Zhang, Z., and Miller, W. (1997) Comparision of DNA sequences with protein sequences, Genomics 46: 24-36*

---

[align Search Help]

---

```
>_ AY769440                                        16309 nt vs.
>_ EF108336                                        16299 nt
scoring matrix: , gap penalties: -12/-2
82.5% identity;         Global alignment score: 44838

               10         20        30        40        50
974467 GTTAATGTAGCTTA-TAATAAAGCAAAGCACTGAAAATGCTTAGATGGATTCAAAAATCC
       :::::::::::::: ::: :::::::::::::::::::::::::::::::      ::::
_      GTTAATGTAGCTTAATAACAAAGCAAAGCACTGAAAATGCTTAGATGGATAATTGTATCC
               10        20        30        40        50        60

      60        70        80        90       100       110
974467 CATAAACACAAAGGTTTGGTCCTGGCCTTATAATTAATTGGAGGTAAGATTACACATGCA
       ::::::::::::::::::::::::::::::::::::::: ::::::: ::::::::::::
_      CATAAACACAAAGGTTTGGTCCTGGCCTTATAATTAATTAGAGGTAAAATTACACATGCA
               70        80        90       100       110       120

     120       130       140       150       160       170
974467 AACATCCATAAACCGGTGTAAAATCCCTTAAACATTTGCCTAAAACTTAAGGAGAGGGCA
       ::: :::::: :::::::::::::::::::::::::: : ::::: :::::::::::: :
_      AACCTCCATAGACCGGTGTAAAATCCCTTAAACATTTACTTAAAATTTAAGGAGAGGGTA
              130       140       150       160       170       180

     180        190       200       210       220       230
974467 TCAAGCACAT-AATATAGCTCAAGACGCCTTGCCTAGCCACACCCCCACGGGACTCAGCA
       :::::::::: :: :::::: ::::: :::::::::::::::::::::::::::::::::
_      TCAAGCACATTAAAATAGCTTAAGACACCTTGCCTAGCCACACCCCCACGGGACTCAGCA
              190       200       210       220       230       240

      240       250       260       270       280       290
974467 GTGATAAATATTAAGCAATGAACGAAAGTTTGACTAAGCTATACCTCTCAGGGTTGGTAA
       ::::::::::::::::::: :::::::::::::::::: ::::::::: :::::::::::
_      GTGATAAATATTAAGCAATAAACGAAAGTTTGACTAAGTTATACCTCTTAGGGTTGGTAA
              250       260       270       280       290       300

      300       310       320       330       340       350
974467 ATTTCGTGCCAGCCACCGCGGTCATACGATTAACCCAAACTAATTATTTTCGGCGTAAAA
       ::::::::::::::::::::::::::::::::::::::::::::::: ::::::::::::
_      ATTTCGTGCCAGCCACCGCGGTCATACGATTAACCCAAACTAATTATCTTCGGCGTAAAA
              310       320       330       340       350       360

      360       370       380       390       400       410
974467 CGTGCCAACTATAAATCTCATAATAGAATTAAAATCCAACTTATATGTGAAAATTCATTG
       :::: :::::::::::     :::::::::::::::::::::::::::::::::::::::
_      CGTGTCAACTATAAATAAATAAATAGAATTAAAATCCAACTTATATGTGAAAATTCATTG
              370       380       390       400       410       420

      420       430       440       450       460       470
974467 TTAGGACCTAAGCCCAATAACGAAAGTAATTCTAATCATTTATATAATGCACGATAGCTA
       ::::::::::: : :::::::::::::::::::: ::::::::  ::: ::::: :::::
_      TTAGGACCTAAACTCAATAACGAAAGTAATTCTAGTCATTTAT--AATACACGACAGCTA
              430       440       450       460         470

      480       490       500       510       520       530
974467 AGACCCAAACTGGGATTAGATACCCCACTATGCTTAGCCCTAAACCTTAATAATTAAACC
       ::::::::::::::::::::::::::::::::::::::: ::::::: ::::::::::
_      AGACCCAAACTGGGATTAGATACCCCACTATGCTTAGCCATAAACCTAAATAATTAAATT
      480       490       500       510       520       530

      540        550       560       570       580       590
974467 TA-CAAAATTATTTGCCAGAGAACTACTAGCTACAGCTTAAAACTCAAAGGACTTGGCGG
       :: ::::: :::::::::::::::::::::: : ::::::::::::::::::::::::::
_      TAACAAAACTATTTGCCAGAGAACTACTAGCCATAGCTTAAAACTCAAAGGACTTGGCGG
      540       550       560       570       580       590

       600       610       620       630       640       650
974467 TACTTTATATCCATCTAGAGGAGCCTGTTCTATAATCGATAAACCCCGTTCTACCTTACC
       :::::::::::::::::::::::::::::::::::::::::::::::: ::::::: :::
```

```
_       TACTTTATATCCATCTAGAGGAGCCTGTTCTATAATCGATAAACCCCGCTCTACCTCACC
      600       610       620       630       640       650

        660       670       680       690       700       710
974467 CCTTCTCGCTAATTCAGCCTATATACCGCCATCTTCAGCAAACCCTAAAAAGGCACTAAA
          ::: :::::::::::::::::::::::::::::::::::::::::::::: : ::::
_       ATCTCTTGCTAATTCAGCCTATATACCGCCATCTTCAGCAAACCCTAAAAAGGTATTAAA
      660       670       680       690       700       710

        720       730       740       750       760       770
974467 GTAAGCACAAGAA-CAAACATAAAAACGTTAGGTCAAGGTGTAGCCAATGAAGCGGAAAG
       ::::::: ::::: ::::::::::::::::::::::::::::::::::::::  :: :::
_       GTAAGCAAAAGAATCAAACATAAAAACGTTAGGTCAAGGTGTAGCCAATGAAATGGGAAG
      720       730       740       750       760       770

         780       790       800       810       820       830
974467 AAATGGGCTACATTTTCTTTTCCCAGAGAACATTACGAAACCCTTTATGAAACTAAAGGA
       ::::::::::::::::::: :   : :::::::::: : :::::::::::::::::::::
_       AAATGGGCTACATTTTCTTAT--AAAAGAACATTACTATACCCTTTATGAAACTAAAGGA
      780       790       800       810       820       830

         840       850       860       870       880       890
974467 CAAAGGAGGATTTAGTAGTAAATTAAGAATAGAGAGCTTAATTGAATAGAGCAATGAAGT
       : ::::::::::::::::::::::::::::::::::::::::::::: ::::::::::::
_       CTAAGGAGGATTTAGTAGTAAATTAAGAATAGAGAGCTTAATTGAATTGAGCAATGAAGT
         840       850       860       870       880       890

         900       910       920       930       940       950
974467 ACGCACACACCGCCCGTCACCCTCCTCAAATTAGATTGACATTCACATATACATAATTTC
       ::::::::::::::::::::::::::::::::: ::: :  :: :::::    :::::::
_       ACGCACACACCGCCCGTCACCCTCCTCAAATTAAATTAAACTTAACATAAT--TAATTTC
         900       910       920       930       940        950

         960        970       980       990      1000      1010
974467 ACTA-ACAAATTTATGAGAGGAGATAAGTCGTAACAAGGTAAGCATACTGGAAAGTGTGC
          : :    ::::::::::::::::::::::::::::::::::::::::::::::::::
_       TAGACATCCGTTTATGAGAGGAGATAAGTCGTAACAAGGTAAGCATACTGGAAAGTGTGC
           960       970       980       990      1000      1010

         1020      1030      1040      1050      1060      1070
974467 TTGGAATAATCACAGTGTAGCTTAATCACAAAGCATCTGGCCTACACCCAGAAGAATTCA
       :::::::::::: :::::::::::::   :::::::::::::::::::::::::: ::::
_       TTGGAATAATCATAGTGTAGCTTAATATTAAAGCATCTGGCCTACACCCAGAAGATTTCA
          1020      1030      1040      1050      1060      1070

          1080      1090      1100      1110       1120      1130
974467 TAAA-AATGAACACTTTGAACTAACCCTAGCCCTACAACCA-ACCAACATAACTAAACCC
       : :  :::::::::: :::::::: ::::::::::   : : :: :: :::: :: ::  
_       TGACCAATGAACACTCTGAACTAATCCTAGCCCTAGCCCTACACAAATATAATTATACTA
          1080      1090      1100      1110      1120      1130

           1140      1150        1160      1170      1180
974467 CCACATAAACTAAAACATTTAACTCA--AAAAGTATTGGAGAAAGAAATT--TACTTACC
         : :::::  :::::::::: :  :  ::::::::::::::::::::::  ::: : : 
_       TTATATAAATCAAAACATTTATCCTACTAAAAGTATTGGAGAAAGAAATTCGTACAT-CT
          1140      1150      1160      1170      1180      1190

    1190      1200      1210      1220      1230      1240
974467 AGGAGCTATAGAGAAAGTACCGCAAGGGAATGATGAAAGACTAATTTAAAGTAAAAACAA
       ::::::::::::   ::::::::::::::: ::::::::::::::: ::::::: :::::
_       AGGAGCTATAGAACTAGTACCGCAAGGGAAAGATGAAAGACTAATTAAAAGTAAGAACAA
           1200      1210      1220      1230      1240      1250

    1250      1260      1270      1280      1290      1300
974467 GCAAAGATTAAACCTTGTACCTTTTGCATAATGAATTAACTAGAAAATCCTTAACAAAAA
       ::::::::::::::::::::::::::::::::::: :::::::::::    :::: ::::
_       GCAAAGATTAAACCTTGTACCTTTTGCATAATGAACTAACTAGAAAACTTCTAACTAAAA
           1260      1270      1280      1290      1300      1310

    1310      1320      1330      1340      1350      1360
974467 GAATTTAAGCTAAGAACCCCGAAACCAAACGAGCTACCTAAAAACAATTTCATGAATCAA
       :::::  :::::  :::::::::::::::::::::::::::::::::::: :::::::::
_       GAATTACAGCTAGAAACCCCGAAACCAAACGAGCTACCTAAAAACAATTTTATGAATCAA
           1320      1330      1340      1350      1360      1370

    1370      1380      1390      1400      1410      1420
974467 CCCGTCTATGTAGCAAAATAGTGGGAAGATTTTTAGGTAGAGGTGAAAAGCCTATCGAGC
       : ::::::::: ::::::::::: :::::::::::::::::::::::::::::: :::::
_       CTCGTCTATGTGGCAAAATAGTGAGAAGATTTTTAGGTAGAGGTGAAAAGCCTAACGAGC
           1380      1390      1400      1410      1420      1430

    1430      1440      1450       1460      1470      1480
974467 TTGGTGATAGCTGGTTGCCCAAAAAA-GAATTTCAGTTCAACTTTAAGCTTACCATCAGA
       :::::::::::::::: ::::::::: :::::: ::::::: ::::: ::: : :  : :
_       TTGGTGATAGCTGGTTACCCAAAAAATGAATTTAAGTTCAATTTTAAACTTGCTAAAAAA
           1440      1450      1460      1470      1480      1490

     1490      1500      1510      1520      1530      1540
974467 ACAACAAA-TCAAAATGTAAACTTAAAATATAGCCAAAAGAGGGACAGCTCTTTAGGAAA
       :::::::: :::::: ::::  ::: : :::::::::::::::::::::::::  :::: 
_       ACAACAAAATCAAAAAGTAAGTTTAGATTATAGCCAAAAGAGGGACAGCTCTTCTGGAAC
           1500      1510      1520      1530      1540      1550
```

```
      1550      1560      1570      1580       1590      1600
974467 AGGAAAAAACCTTAAATAGTGAATAAACAACTACA-ATCACTTAACCATTGTAGGCTTAA
        :::::::::::: ::::::::::::  ::: : : : :  ::::::::::::::: :::
_      -GGAAAAAACCTTTAATAGTGAATAATTAACAAAACAGCTTTTAACCATTGTAGGCCTAA
           1560      1570      1580      1590      1600      1610

       1610      1620      1630      1640      1650      1660
974467 AAGCAGCCATCAATAAAGAAAGCGTTCAAGCTCAACATACATACTTACACACACACTAAT
       ::::::::: ::::::::::::::::::::::::::::: :   :: ::     : ::::
_      AAGCAGCCACCAATAAAGAAAGCGTTCAAGCTCAACATAAA--ATTTCA-----ATTAAT
           1620      1630      1640      1650             1660

       1670      1680      1690      1700      1710      1720
974467 TCCACAAACCTCAATAAATTCCTATATTACAAATTGGGCTAATCTATAGATCCATAGATG
       :::: ::    ::  :: :::::: : :  :::::::: :::::::::  :  :::::::
_      TCCATAATTTACACCAACTTCCTAAACTTAAAATTGGGTTAATCTATAACTTTATAGATG
        1670      1680      1690      1700      1710      1720

       1730      1740      1750        1760      1770      1780
974467 AAATACTGTTAATATGAGTAACAAGAA--CCAATTCTCCTAGCACAAGTGTATGACAACC
        :: ::::::: :::::::::::::::  ::::::::::  ::: : : :::: :::::
_      CAACACTGTTAGTATGAGTAACAAGAATTCCAATTCTCCAGGCATACGCGTATAACAACT
        1730      1740      1750      1760      1770      1780

         1790      1800       1810      1820      1830      1840
974467 CGGATAACCATTGTCAATTA-TCGAATCATAGGTACTAACCCAACAATAGAATTACCTAT
       :::::::::::::: : ::: ::  :  ::::: : ::: :  :: ::: ::: : : :
_      CGGATAACCATTGTTAGTTAATCAGACTATAGGCAATAATCACACTATA-AATAATCCAC
        1790      1800      1810      1820      1830       1840

          1850          1860      1870      1880      1890
974467 CCCTAACTC----GTTAGCCCAACACAGGCGTGCTTTAAGGAAAGATTAAAAAAAGTAAA
       :  :::::     :::: :::::::: ::  ::: : ::::::::::  :::::  ::::
_      CTATAACTTCTCTGTTAACCCAACACCGGAATGCCTAAAGGAAAGATCCAAAAAGATAAA
         1850      1860      1870      1880      1890      1900

    1900      1910      1920      1930      1940      1950
974467 AGGAACTCGGCAAACACGAACCCCGCCTGTTTACCAAAAACATCACCTCTAGCATAACAA
       :::::::::::::::: :::::::::::::::::::::::::::::::::::::: ::::
_      AGGAACTCGGCAAACAAGAACCCCGCCTGTTTACCAAAAACATCACCTCTAGCATTACAA
         1910      1920      1930      1940      1950      1960

    1960      1970      1980      1990      2000      2010
974467 GTATTAGAGGCATTGCCTGCCCAGTGACTAAAGTTAAACGGCCGCGGTATCCTGACCGTG
       :::::::::::: :::::::::::::::::::::: ::::::::::::::::::::::::
_      GTATTAGAGGCACTGCCTGCCCAGTGACTAAAGTTTAACGGCCGCGGTATCCTGACCGTG
         1970      1980      1990      2000      2010      2020

    2020      2030      2040      2050      2060      2070
974467 CAAAGGTAGCATAATCACTTGTTCCTTAATTAGGGACTAGAATGAATGGCTAAACGAGGG
       :::::::::::::::::::::::::::::::::::::::: ::::: :::::::::::::
_      CAAAGGTAGCATAATCACTTGTTCCTTAATTAGGGACTAGCATGAACGGCTAAACGAGGG
         2030      2040      2050      2060      2070      2080

    2080      2090      2100      2110      2120      2130
974467 TTCAACTGTCTCTTACTTTCAATCAGTGAAATTGACCTTCCAGTGAAGAGGCTGGAATCT
       : :::::::::::::  :: ::::::::::::::::::: :::::::::::::: ::: :
_      TCCAACTGTCTCTTATCTTTAATCAGTGAAATTGACCTTTCAGTGAAGAGGCTGAAATAT
         2090      2100      2110      2120      2130      2140

    2140      2150      2160      2170      2180      2190
974467 CCCAATAAGACGAGAAGACCCTATGGAGCTTTAATTTACTAGTTCAACTTATATAA---A
          :::::::::::::::::::::::::::: ::::   ::  : : :: :: :::   :
_      AATAATAAGACGAGAAGACCCTATGGAGCTTAAATTATATAACTTATCT-ATTTAATTTA
         2150      2160      2170      2180      2190       2200

       2200      2210      2220      2230      2240      2250
974467 AACAACCTAATGGGCTAAAACAAAATAAATATGAACTAAAAAATTTCGGTTGGGGTGACC
          :::::::::: : :::: :  :::  ::: :: :   ::::::::::::::::::::
_      TTAAACCTAATGGCCCAAAA-ACTATAG-TAT-AAGTTTGAAATTTCGGTTGGGGTGACC
          2210      2220       2230        2240      2250      2260

       2260      2270      2280      2290      2300      2310
974467 TCGGAGAATAAAAAATCCTCCGAATGATTTTAACCTAGACTCACAAGTCAAAGTAATACT
       ::::::::::::::::::::::::::::: ::::::::::: :::::::::::::: :
_      TCGGAGAATAAAAAATCCTCCGAATGATTATAACCTAGACTTACAAGTCAAAGTAAAATC
             2270      2280      2290      2300      2310      2320

         2320      2330          2340      2350      2360      2370
974467 AA--TATCTTATTGACCCAATTAT----TGATCAACGGACCAAGTTACCCTAGGGATAAC
       ::  :::::::::::::::  :::    ::::::::::::::::::::::::::::::::
_      AACATATCTTATTGACCCAGATATATTTTGATCAACGGACCAAGTTACCCTAGGGATAAC
             2330      2340      2350      2360      2370      2380

             2380      2390      2400      2410      2420      2430
974467 AGCGCAATCCTATTTAAGAGTTCATATCGACAATTAGGGTTTACGACCTCGATGTTGGAT
       ::::::::::::::::::::::::::::::::::::::::::::::::::::::::::::
_      AGCGCAATCCTATTTAAGAGTTCATATCGACAATTAGGGTTTACGACCTCGATGTTGGAT
             2390      2400      2410      2420      2430      2440

             2440      2450      2460      2470      2480      2490
974467 CAGGACATCCCAATGGTGCAGAAGCTATTAATGGTTCGTTTGTTCAACGATTAAAGTCCT
       :::::::::::::::::: :::::::::::::::::::::::::::::::::::::::::
```

```
_      CAGGACATCCCAATGGTGTAGAAGCTATTAATGGTTCGTTTGTTCAACGATTAAAGTCCT
             2450      2460      2470      2480      2490      2500

             2500      2510      2520      2530      2540      2550
974467 ACGTGATCTGAGTTCAGACCGGAGCAATCCAGGTCGGTTTCTATCTATTTACAATTTCTC
       :::::::::::::::::::::::::::::::::::::::::::::::::::: :::::::
_      ACGTGATCTGAGTTCAGACCGGAGCAATCCAGGTCGGTTTCTATCTATTTACGATTTCTC
             2510      2520      2530      2540      2550      2560

             2560      2570      2580       2590      2600
974467 CCAGTACGAAAGGACAAGAGAAATGGAGCCTCCTTACCA-TAAGTGCTCCCAACC-AATT
       :::::::::::::::::::::::: ::::: :::::: : :::: :::: ::::  ::::
_      CCAGTACGAAAGGACAAGAGAAATAGAGCCACCTTACAAATAAGCGCTCTCAACTTAATT
             2570      2580      2590      2600      2610      2620

     2610      2620      2630      2640      2650      2660
974467 TATGAAAAAAATCTCAATAAAGTATATATGTACAATAAATAAACCTAGCCCAGGTTATTA
       :::::: ::::::: :::::: :::::: :::::     ::: :::::   :::::::::
_      TATGAATAAAATCTAAATAAAATATATACGTACACCCTCTAA-CCTAGAGAAGGTTATTA
             2630      2640      2650      2660       2670

     2670      2680      2690      2700      2710      2720
974467 GGGTGGCAGAGCCAGGTAATTGCGTAAGACTTAAAACCTTGTTCCCAGAGGTTCAAATCC
       :::::::::::::::: :::::::::::::::::::::::::::::::::::::::::::
_      GGGTGGCAGAGCCAGGAAATTGCGTAAGACTTAAAACCTTGTTCCCAGAGGTTCAAATCC
    2680      2690      2700      2710      2720      2730

     2730      2740      2750      2760      2770      2780
974467 TCTCCCTAATAGTGTACTTTATTAATATCCTAACACTCCTAATCCCAATCTTAATTGCCA
       ::::::::::::::: ::::::::::::::::::::::::  :::: ::  :::: ::::
_      TCTCCCTAATAGTGTTCTTTATTAATATCCTAACACTCCTCGTCCCCATTCTAATCGCCA
    2740      2750      2760      2770      2780      2790

     2790      2800      2810      2820      2830      2840
974467 TGGCCTTCCTCACCCTAGTAGAACGGAAAATCCTAGGCTACATACAATTACGCAAAGGCC
       : :::::::: ::  :::::::::: :::::: :::: ::::::::: :::: :::::::
_      TAGCCTTCCTAACATTAGTAGAACGCAAAATCTTAGGGTACATACAACTACGAAAAGGCC
    2800      2810      2820      2830      2840      2850

     2850      2860      2870      2880      2890      2900
974467 CCAACATCGTAGGCCCATATGGTATTCTACAACCATTTGCAGATGCCATAAAACTATTCA
       : ::::: :: :: ::::: :: ::: :::::::::::::::: ::::::::: :::: :
_      CTAACATTGTTGGTCCATACGGCATTTTACAACCATTTGCAGACGCCATAAAATTATTTA
    2860      2870      2880      2890      2900      2910

     2910      2920      2930      2940      2950      2960
974467 TAAAAGAACCCATACGCCCTCTAACCACCTCAATATCACTATTTATTATCGCCCCAACCC
       :::::::::: ::::::::: :::: ::::: :::::  :::::::::: :: :: ::::
_      TAAAAGAACCAATACGCCCTTTAACAACCTCTATATCCTTATTTATTATTGCACCTACCC
    2920      2930      2940      2950      2960      2970

     2970      2980      2990      3000      3010      3020
974467 TCTCCCTTACACTAGCTCTAAGCCTATGAATTCCCTTACCAATACCTCACCCCCTTATCA
       : :: :: ::::::::  :::: :::::: ::::: :::::::::: :::::  : :: :
_      TATCACTCACACTAGCATTAAGTCTATGAGTTCCCCTACCAATACCACACCCATTAATTA
    2980      2990      3000      3010      3020      3030

     3030      3040      3050      3060      3070      3080
974467 ACCTCAACCTAGGCATACTATTTATTCTAGCCACATCAAGTCTTTCAGTCTACTCCATTC
       :  : :::::::: ::  :::::::: :::: ::::: :: :: ::::: ::::::::::
_      ATTTAAACCTAGGGATTTTATTTATTTTAGCAACATCTAGCCTATCAGTTTACTCCATTC
    3040      3050      3060      3070      3080      3090

     3090      3100      3110      3120      3130      3140
974467 TATGATCAGGATGAGCATCAAATTCAAAATACTCCCTATTCGGAGCCCTACGAGCCGTTG
       :::::::::::::::: ::::: :: :::::::: :::::::::::  :::::::::: :
_      TATGATCAGGATGAGCCTCAAACTCCAAATACTCACTATTCGGAGCTTTACGAGCCGTAG
    3100      3110      3120      3130      3140      3150

     3150      3160      3170      3180      3190      3200
974467 CCCAAACCATCTCTTACGAAGTCACAATAGCCATTATCCTCTTATCCGTCCTCCTAATAA
       ::::::: :: :: :: ::::: :: ::::: :::::::: ::::: :: ::  ::::::
_      CCCAAACAATTTCATATGAAGTAACCATAGCTATTATCCTTTTATCAGTTCTATTAATAA
    3160      3170      3180      3190      3200      3210

     3210      3220      3230      3240      3250      3260
974467 GCGGCTCCTTCTCCCTACAAATACTTATCACTACACAAGAACATATCTGACTATTAATCC
         :: :::: ::: ::::::: :::::: :: :: :::::::: :: ::: :: :  : :
_      ATGGATCCTACTCTCTACAAACACTTATTACAACCCAAGAACACATATGATTACTTCTGC
    3220      3230      3240      3250      3260      3270

     3270      3280      3290      3300      3310      3320
974467 CCGCCTGACCAATAGCCATAATATGATACATTTCAACCCTAGCAGAAACAAATCGAGCTC
       : :::::::: ::::::::::::::::  :: :::::::::::::::::::: :: :: :
_      CAGCCTGACCCATAGCCATAATATGATTTATCTCAACCCTAGCAGAAACAAACCGGGCCC
    3280      3290      3300      3310      3320      3330

     3330      3340      3350      3360      3370      3380
974467 CCTTCGACTTAACAGAAGGAGAATCAGAATTAGTCTCAGGCTTTAACGTCGAATACGCCG
       :::::::: : ::::::::::::::::::::::: ::::: :::::::: :::::::: :
_      CCTTCGACCTGACAGAAGGAGAATCAGAATTAGTATCAGGGTTTAACGTAGAATACGCAG
    3340      3350      3360      3370      3380      3390
```

```
     3390      3400      3410      3420      3430      3440
974467 CAGGACCATTCGCCCTATTCTTCATAGCCGAGTACACCAACATTATTCTAATAAACGCCC
       : :: ::::::::  ::::::: ::::: :::::::: ::::::::::::::::::::::
_      CCGGCCCATTCGCGTTATTCTTTATAGCAGAGTACACTAACATTATTCTAATAAACGCCC
    3400      3410      3420      3430      3440      3450

     3450      3460      3470      3480      3490      3500
974467 TAACATCAATTGTATTCCTAGGCCCCTTATATCATATCAATTACCCTGAATTATACTCAA
       ::::: : ::: : :::::::: ::: ::::  :::::::::  :: ::: : :::::::
_      TAACAACTATTATCTTCCTAGGACCCCTATACTATATCAATTTACCAGAACTCTACTCAA
    3460      3470      3480      3490      3500      3510

     3510      3520      3530      3540      3550      3560
974467 CCAGCTTCATAACAGAAACACTACTTCTATCCACAACTTTCCTATGAATCCGAGCATCCT
       : : :::::::: :::: : ::::: :::::  :::: :::::::: ::::::::::: :
_      CTAACTTCATAATAGAAGCTCTACTACTATCATCAACATTCCTATGGATCCGAGCATCTT
    3520      3530      3540      3550      3560      3570

     3570      3580      3590      3600      3610      3620
974467 ACCCCCGTTTTCGATATGACCAACTAATGCACCTCCTATGAAAAAATTTCCTCCCACTAA
       : :: :: :: :: :: :: ::::: :: :: :: ::::::::::: :: :: :: ::::
_      ATCCACGCTTCCGTTACGATCAACTTATACATCTTCTATGAAAAAACTTTCTACCCCTAA
    3580      3590      3600      3610      3620      3630

     3630      3640      3650      3660      3670      3680
974467 CACTAGCATTCTGCATATGATACATTTCCCTGCCAATTTTCCTAGCAGGAATTCCACCCT
       :::::::::: :: :: ::: : :::::  : ::::::::   ::: ::: : ::::: :
_      CACTAGCATTATGTATGTGACATATTTCTTTACCAATTTTTACAGCGGGAGTACCACCAT
    3640      3650      3660      3670      3680      3690

     3690      3700      3710      3720      3730      3740
974467 ACACATAGAAATATGTCTGACAAAAGAGTTACTTTGATAGAGTAAATAATAGAGGTTTAA
       ::: :::::::::::::::: :::::: :::::::::::::::::::: :::::::: ::
_      ACATATAGAAATATGTCTGATAAAAGAATTACTTTGATAGAGTAAATTATAGAGGTTCAA
    3700      3710      3720      3730      3740      3750

     3750      3760      3770      3780      3790      3800
974467 ATCCTCTTATTTCTAGGACAATAGGAATTGAACCTACACCTAAGAATTCAAAATTCTCCG
         ::::::::::::::::::::::::::::::::::::: ::::::::::::::::::::
_      GCCCTCTTATTTCTAGGACAATAGGAATTGAACCTACACTTAAGAATTCAAAATTCTCCG
    3760      3770      3780      3790      3800      3810

     3810      3820      3830      3840      3850      3860
974467 TGCTACCAATACACCCTATCCTACATAGTAAGGTCAGCTAACTAAGCTATCGGGCCCATA
       ::::::: : ::::: ::::::: ::::::::::::::::: ::::::::::::::::::
_      TGCTACCTAAACACCTTATCCTA-ATAGTAAGGTCAGCTAATTAAGCTATCGGGCCCATA
    3820      3830      3840      3850      3860      3870

     3870      3880      3890      3900      3910      3920
974467 CCCCGAAAATGTTGGTCTAAACCCTTCCCGTACTAATAAACCCAATCACCCTAATCATTA
       ::::::::: :::::: :::: :::::::::::::::::: :: ::::::::   ::: :
_      CCCCGAAAACGTTGGTTTAAATCCTTCCCGTACTAATAAATCCTATCACCCTTGCCATCA
     3880      3890      3900      3910      3920      3930

     3930      3940      3950      3960      3970      3980
974467 TTTACTTTACTATCCTCATAGGGCCTGTAATCACTATATCTAGCTCCAACTTACTCCTAA
       : ::::: :: ::: :: :::: ::::::::::: ::::: ::: ::::: :: : ::::
_      TCTACTTCACAATCTTCTTAGGTCCTGTAATCACAATATCCAGCACCAACCTAATACTAA
     3940      3950      3960      3970      3980      3990

     3990      4000      4010      4020      4030      4040
974467 TATGAGTAGGATTGGAAATAAGCCTTTTAGCTATCATCCCACTTCTAGCCAACAAAAAAA
       ::::::::::  ::::: : :::::  :::: :: :::::  : :::  :::::::::::
_      TATGAGTAGGCCTGGAATTCAGCCTACTAGCAATTATCCCCATACTAATCAACAAAAAAA
     4000      4010      4020      4030      4040      4050

     4050      4060      4070      4080      4090      4100
974467 GCCCACGATCAACTGAAGCAGCAACAAAATATTTTCTAACCCAAGCTACAGCCTCAATAA
        :::::::::::::::::::::::::::::: ::  : :: ::::: :::::::::::::
_      ACCCACGATCAACTGAAGCAGCAACAAAATACTTCGTCACACAAGCAACAGCCTCAATAA
     4060      4070      4080      4090      4100      4110

     4110      4120      4130      4140      4150      4160
974467 TTATCCTACTAGTCATCATCCTCAACTACAAACAATCAGGAATATGAACCCTCCAACAAC
       ::::::: :: : :::: : :::::::: ::::::  ::::: :::::   : :::::::
_      TTATCCTCCTGGCCATCGTACTCAACTATAAACAACTAGGAACATGAATATTTCAACAAC
     4120      4130      4140      4150      4160      4170

     4170      4180      4190      4200      4210      4220
974467 AAACCAATAACATACTACTCAACATAATACTCATTTCACTAGCCATAAAACTTGGACTAG
       :::: ::     :  : :: ::::::: : : ::  : ::: :::::::::: :: :: :
_      AAACAAACGGTCTTATCCTTAACATAACATTAATAGCCCTATCCATAAAACTAGGCCTCG
     4180      4190      4200      4210      4220      4230

     4230      4240      4250      4260      4270      4280
974467 CCCCATTCCACTACTGACTACCCGAAGTCACCCAAGGAATTCCCCTACACATTGGATTAA
       :::::::::::: :::: :::: ::::: :: ::::: :: :: :: ::::: ::: : :
_      CCCCATTCCACTTCTGATTACCAGAAGTAACTCAAGGGATCCCACTGCACATAGGACTTA
     4240      4250      4260      4270      4280      4290

     4290      4300      4310      4320      4330      4340
974467 TCTTACTAACATGACAAAAAAATTGCTCCACTATCAATTCTATACCAATTTTATCAACTCC
       :  : :: :::::::::::::::::::: ::::::::: ::   ::: :::: :  :: :
```

```
_      TTCTTCTTACATGACAAAAAATTGCTCCCCTATCAATTTTAATTCAAATTTACCCGCTAC
     4300      4310      4320      4330      4340      4350

     4350      4360      4370      4380      4390      4400
974467 TAAACCCAACTATCACCACCATTCTCGCAATTTCATCAGTCTTTGTTGGCGCCTGAGGAG
       : ::: : :::::::     :: :: :::::: : ::  : ::  : :: :: :::::::
_      TCAACTCTACTATCATTTTAATACTAGCAATTACTTCTATTTTCATAGGGGCATGAGGAG
     4360      4370      4380      4390      4400      4410

     4410      4420      4430      4440      4450      4460
974467 GACTTAACCAGACCCAAACACGAAAAATCATAGCATACTCATCAATTGCCCACATAGGAT
       :::::::::: :: :::: ::::::::: ::::: :: ::::::::::::::::::::::
_      GACTTAACCAAACACAAATACGAAAAATTATAGCCTATTCATCAATTGCCCACATAGGAT
     4420      4430      4440      4450      4460      4470

     4470      4480      4490      4500      4510      4520
974467 GAATAACAGCAATCCTTCCATACAACCCTAACTTAACCCTCCTAAACTTAACAATTTACA
       :::::  :::::: ::::: ::::::::   : : :: :: :: ::: : : ::: :: :
_      GAATATTAGCAATTCTTCCTTACAACCCATCCCTCACTCTACTCAACCTCATAATCTATA
     4480      4490      4500      4510      4520      4530

     4530      4540      4550      4560      4570      4580
974467 TCCTACTTACTGTTCCAATATTCATTACACTCATAACAAACTCAGCAACAACAATCAACA
       :  : ::::: :  :: ::::::::  :::: :::  :::     : : ::: ::::::
_      TTATTCTTACAGCCCCTATATTCATAGCACTTATACTAAATAACTCTATAACCATCAACT
     4540      4550      4560      4570      4580      4590

     4590      4600      4610      4620      4630      4640
974467 CACTCTCACTCGCATGAAATAAAACTCCCATAATCCTAACCATAGCATCCATCATCCTCC
       :: :::::::   :::::::::::::::   ::: ::::: :::   ::  : ::  : :
_      CAATCTCACTTCTATGAAATAAAACTCCAGCAATACTAACTATAATCTCACTGATATTAC
     4600      4610      4620      4630      4640      4650

     4650      4660      4670      4680      4690      4700
974467 TATCACTAGGAGGACTCCCCCCTCTCACAGGATTTTTACCAAAATGAGCAATTATCTCCG
       :::: :::::::: :: :: :: :: :::::::: ::::::::::::   :: ::: : :
_      TATCCCTAGGAGGCCTTCCACCACTAACAGGATTCTTACCAAAATGAATTATCATCACAG
     4660      4670      4680      4690      4700      4710

     4710      4720      4730      4740      4750      4760
974467 AGCTTCTAAAAAACAACTGCTCAACCCTATCAACACTAATAGCTATCATAGCCCTATTAA
       : ::: :::::::::::::   ::   :: ::::::: ::::: :: ::::: ::: :::
_      AACTTATAAAAAACAACTGTCTAATTATAGCAACACTCATAGCAATAATAGCTCTACTAA
     4720      4730      4740      4750      4760      4770

     4770      4780      4790      4800      4810      4820
974467 GCCTATTCTTCTATACTCGACTAATTTACTCTATATCCCTCACCACATTCCCAACCAACA
        ::::::::: :::::::: :::::::: :: :  :: :: :: : ::: ::::::::::
_      ACCTATTCTTTTATACTCGCCTAATTTATTCCACTTCACTAACAATATTTCCAACCAACA
     4780      4790      4800      4810      4820      4830

     4830      4840      4850      4860      4870      4880
974467 ACAACTCCAAAATAATCTCCCACCACCAAAACCCAAAACATAATTTTATCCTCCCAACCC
       : ::::: ::::::::  : :::::   :::  : ::::  ::  : ::  :  : ::::
_      ATAACTCAAAAATAATAACTCACCAAACAAAAACTAAACCCAACCTAATATTTTCCACCC
     4840      4850      4860      4870      4880      4890

     4890      4900      4910      4920      4930      4940
974467 TCACAGTATTAAGTACCCTTACCCTACCGCTTTCCTCCCAACTAATCACATAGAAGTTTA
       :  :  :  :::: ::  : :::::::: ::  :: :::::::::: :: ::::::::::
_      TAGCTATCATAAGCACAATAACCCTACCCCTAGCCCCCCAACTAATTACCTAGAAGTTTA
     4900      4910      4920      4930      4940      4950

     4950       4960      4970      4980      4990      5000
974467 GGATATAC-AGTCCAAGAGCCTTCAAAGCCCTTAGAAAACAAACAAGTTTAACTTCTGAT
       :::::::: :::::  :::::::::::::::: :::::::: ::::::::::::::::::
_      GGATATACTAGTCCGCGAGCCTTCAAAGCCCTAAGAAAACACACAAGTTTAACTTCTGAT
     4960      4970      4980      4990      5000      5010

      5010      5020      5030      5040      5050      5060
974467 AAGGACTGTAAGACTATATCTTACATCTGTTAAATGCAAATCAACTGCTTTAATTAAGCT
       :::::::::::::::  ::: ::::::: :: :::::::::::: :::::::::::::::
_      AAGGACTGTAAGACTTCATCCTACATCTATTGAATGCAAATCAATTGCTTTAATTAAGCT
     5020      5030      5040      5050      5060      5070

      5070      5080      5090      5100      5110      5120
974467 AAATCCTCAACTAGATTGGAAGGATTCAAACCTACGAAAATTTAGTTAACAGCTAAATAC
       ::  :::::::::::::::: :::: : ::::::::::::::::::::::::::::::::
_      AAGACCTCAACTAGATTGGCAGGAATTAAACCTACGAAAATTTAGTTAACAGCTAAATAC
     5080      5090      5100      5110      5120      5130

      5130      5140      5150      5160      5170       5180
974467 CCTACTTACTGGCTTCAATCTACTTCTCCCGCCTATCAGAAAAGA-GGCGGGAGAAGCCT
       :::: :::::::::::::::::::::: ::::: :  : :::: : ::::: ::::: ::
_      CCTA-TTACTGGCTTCAATCTACTTCTACCGCCGAAAAAAAAAAATGGCGGTAGAAGTCT
     5140       5150      5160      5170      5180      5190

       5190      5200      5210      5220      5230      5240
974467 TAGTAGAGGAGATTCTCTACACCTTCGAATTTGCAATTCGACATGATAATCACCTTAAGG
       ::::::::    ::::::::::::::::::::::::::::::::::  :::::::::::
_      TAGTAGAGA--TTTCTCTACACCTTCGAATTTGCAATTCGACATGAATATCACCTTAAGA
      5200        5210      5220      5230      5240      5250
```

```
        5250      5260      5270       5280      5290      5300
974467 CTTTTTGGTAAAAAGGGGGCTCAACCC-CTGTCTTTAGATTTACAGTCTAATGCTTACTC
       : : : ::::::::: ::  : :: :: :::: :::::::::::::::::::::::::::
_      CCTCT-GGTAAAAAGAGGATTTAAACCTCTGTGTTTAGATTTACAGTCTAATGCTTACTC
         5260       5270      5280      5290      5300      5310

         5310      5320      5330      5340      5350      5360
974467 AGCCATTTTACCTATGTTCGTAAACCGTTGACTCTTTTCAACTAACCACAAAGATATCGG
       ::::::::::::::::::: : :: :::::: : :: ::::: :: ::::::::::::::
_      AGCCATTTTACCTATGTTCATTAATCGTTGATTATTCTCAACCAATCACAAAGATATCGG
          5320      5330      5340      5350      5360      5370

         5370      5380      5390      5400      5410      5420
974467 AACCCTCTACCTATTATTTGGAGCCTGAGCAGGAATAGTAGGGACAGCTTTAAGTATTCT
       ::::::::: ::: :::: ::::::::::: :::::::: :: :: ::  :::::::: :
_      AACCCTCTATCTACTATTCGGAGCCTGAGCGGGAATAGTGGGTACTGCACTAAGTATTTT
          5380      5390      5400      5410      5420      5430

         5430      5440      5450      5460      5470      5480
974467 AATTCGAGCTGAACTAGGACAGCCAGGCGCACTCCTAGGAGATGACCAAATCTATAATGT
       ::::::::: ::: :::: :: ::::: :::::  :::::::::::::::: :: :::::
_      AATTCGAGCAGAATTAGGTCAACCAGGTGCACTTTTAGGAGATGACCAAATTTACAATGT
          5440      5450      5460      5470      5480      5490

         5490      5500      5510      5520      5530      5540
974467 CATCGTCACAGCCCATGCATTCGTAATAATTTTCTTTATAGTAATACCTATAATAATTGG
        ::::: :: :::::::: :: :: ::::::::::: ::::::::::: :::::::::::
_      TATCGTAACTGCCCATGCTTTTGTTATAATTTTCTTCATAGTAATACCAATAATAATTGG
          5500      5510      5520      5530      5540      5550

         5550      5560      5570      5580      5590      5600
974467 AGGCTTCGGAAACTGACTTGTACCACTAATAATTGGAGCCCCTGATATAGCATTCCCACG
       :::::: :::::::::::::: ::::::::::: ::::::::: ::::::::::::::::
_      AGGCTTTGGAAACTGACTTGTCCCACTAATAATCGGAGCCCCAGATATAGCATTCCCACG
          5560      5570      5580      5590      5600      5610

         5610      5620      5630      5640      5650      5660
974467 AATAAATAACATAAGCTTTTGACTGCTTCCTCCATCATTTCTACTCCTTTTAGCATCCTC
       ::::::::: ::::: :::::::: :: :: ::::::::::: :: ::  ::::::: ::
_      AATAAATAATATAAGTTTTTGACTCCTACCACCATCATTTCTCCTTCTCCTAGCATCATC
          5620      5630      5640      5650      5660      5670

         5670      5680      5690      5700      5710      5720
974467 CATAGTAGAAGCTGGAGCTGGAACAGGATGAACAGTATATCCCCCCTTAGCCGGAAACCT
        ::::::::::: ::::: ::::::::::::::::: :: :: ::  :::::::::: ::
_      AATAGTAGAAGCAGGAGCAGGAACAGGATGAACAGTCTACCCACCTCTAGCCGGAAATCT
          5680      5690      5700      5710      5720      5730

         5730      5740      5750      5760      5770      5780
974467 AGCCCATGCTGGGGCATCCGTAGATTTAACTATTTTTTCCCTCCACCTAGCCGGGGTGTC
       ::::::::: :: ::::: :::::  :::: ::::: ::::: ::  :::: :: :::::
_      AGCCCATGCAGGAGCATCAGTAGACCTAACAATTTTCTCCCTTCATTTAGCTGGAGTGTC
          5740      5750      5760      5770      5780      5790

         5790      5800      5810      5820      5830      5840
974467 TTCTATCTTAGGAGCTATCAACTTTATCACCACTATCATTAATATAAAACCCCCTGCTAT
        ::::: ::::: :: :: :: ::::: :::::::: :: :: :: :::::::: :: ::
_      ATCTATTTTAGGTGCAATTAATTTTATTACCACTATTATCAACATGAAACCCCCAGCCAT
          5800      5810      5820      5830      5840      5850

         5850      5860      5870      5880      5890      5900
974467 AACCCAATATCAGACACCTCTCTTTGTATGATCCGTACTAATTACAGCCGTCCTACTACT
       ::: :: ::::: :: :: :: ::::: ::::::::::: ::::::::::: :: :: ::
_      AACACAGTATCAAACTCCACTATTTGTCTGATCCGTACTTATTACAGCCGTACTGCTCCT
          5860      5870      5880      5890      5900      5910

         5910      5920      5930      5940      5950      5960
974467 TCTCTCACTGCCAGTATTAGCAGCAGGTATCACTATACTCCTTACAGACCGAAATCTAAA
         : ::::: :::::  :::: ::::: :: :::::::: :: :::::::: :: :::::
_      ATTATCACTACCAGTGCTAGCCGCAGGCATTACTATACTACTAACAGACCGCAACCTAAA
          5920      5930      5940      5950      5960      5970

         5970      5980      5990      6000      6010      6020
974467 TACTACTTTCTTCGACCCCGCTGGAGGTGGAGACCCAATTCTTTATCAACACCTATTCTG
        :: :::::::: :: ::::::::::: :: ::::::::::: :: :: :: :: :::::
_      CACAACTTTCTTTGATCCCGCTGGAGGAGGGGACCCAATTCTCTACCAGCATCTGTTCTG
          5980      5990      6000      6010      6020      6030

         6030      6040      6050      6060      6070      6080
974467 ATTCTTCGGCCACCCAGAAGTGTACATCTTAATTCTTCCAGGGTTTGGAATTATTTCACA
       :::::: :: ::::::::::: :: ::  : :: :: ::::: :::::::::::::::::
_      ATTCTTTGGGCACCCAGAAGTTTATATTCTTATCCTCCCAGGATTTGGAATTATTTCACA
          6040      6050      6060      6070      6080      6090

         6090      6100      6110      6120      6130      6140
974467 TGTAGTTACCTATTACTCTGGAAAAAAAGAACCCTTCGGATATATAGGTATGGTATGAGC
       ::::::::: :: ::::: :::::::::::::: ::::: :::::::: :: ::::::::
_      TGTAGTTACTTACTACTCCGGAAAAAAAGAACCTTTCGGCTATATAGGAATAGTATGAGC
          6100      6110      6120      6130      6140      6150

         6150      6160      6170      6180      6190      6200
974467 CATAATATCTATTGGCTTCCTAGGATTTATTGTATGAGCACATCACATATTCACAGTAGG
        ::::: ::::::::::: ::::: :::::::::::::: :: :::::::::::::::::
```

```
_      AATAATGTCTATTGGCTTTCTAGGCTTTATTGTATGAGCCCACCACATATTCACAGTAGG
         6160      6170      6180      6190      6200      6210

        6210      6220      6230      6240      6250      6260
974467 CCTAGATGTAGACACCCGAGCCTACTTTACATCTGCCACTATAATTATCGCAATTCCTAC
         ::::::::::::: ::::: ::::::::::: ::::::::::::::::::::::::::
_      ATTAGATGTAGACACACGAGCTTACTTTACATCAGCCACTATAATTATCGCAATTCCTAC
         6220      6230      6240      6250      6260      6270

        6270      6280      6290      6300      6310      6320
974467 AGGCGTAAAAGTATTCAGCTGACTCGCTACACTACATGGAGGAAATATCAAATGATCCCC
        :: :: :::::::: :::::::: :: :: ::::: ::::: ::::: :::::::: ::
_      CGGTGTCAAAGTATTTAGCTGACTTGCAACCCTACACGGAGGTAATATTAAATGATCTCC
         6280      6290      6300      6310      6320      6330

        6330      6340      6350      6360      6370      6380
974467 CGCCATATTATGAGCCTTAGGGTTTATCTTCTTATTCACAGTAGGGGGCCTAACAGGGAT
        :: ::: ::::::::::::: ::::: :::::::: ::::: :: :: ::::: :: ::
_      AGCTATACTATGAGCCTTAGGCTTTATTTTCTTATTTACAGTTGGTGGTCTAACCGGAAT
         6340      6350      6360      6370      6380      6390

        6390      6400      6410      6420      6430      6440
974467 CGTACTATCTAACTCATCCCTTGACATTGTACTTCATGATACATACTACGTAGTAGCTCA
        ::  :::: ::::::::::::::::: :: ::::: ::::::::::: :::::::: ::
_      TGTTTTATCCAACTCATCCCTTGACATCGTGCTTCACGATACATACTATGTAGTAGCCCA
         6400      6410      6420      6430      6440      6450

        6450      6460      6470      6480      6490      6500
974467 CTTCCACTATGTCTTATCTATAGGAGCAGTATTCGCCATCATAGCTGGCTTCGTCCACTG
        :::::::::::  :::: :: :::::::: :: :: :::::::: :: :: :: :::::
_      TTTCCACTATGTTCTATCAATGGGAGCAGTGTTTGCTATCATAGCAGGATTTGTTCACTG
         6460      6470      6480      6490      6500      6510

        6510      6520      6530      6540      6550      6560
974467 ATTCCCACTATTCTCAGGCTATACCCTAAATGACACATGAGCAAAAGCCCACTTTGCCAT
       ::::::: :::: :::::::  :::::: ::::::::::::::::::::::::: :::::
_      ATTCCCATTATTTTCAGGCTTCACCCTAGATGACACATGAGCAAAAGCCCACTTCGCCAT
         6520      6530      6540      6550      6560      6570

        6570      6580      6590      6600      6610      6620
974467 TATATTTGTAGGTGTAAACATAACATTTTTTCCTCAACACTTCCTAGGATTAGCAGGGAT
        ::::: ::::: :::::::::::::: :: :::::::: ::::: ::  :  :::: ::
_      CATATTCGTAGGAGTAAACATAACATTCTTCCCTCAACATTTCCTGGGCCTTTCAGGAAT
         6580      6590      6600      6610      6620      6630

        6630      6640      6650      6660      6670      6680
974467 ACCTCGTCGTTACTCTGATTATCCAGATGCTTATACCACATGAAATACAGTCTCCTCTAT
       ::: :: :: ::::: :: :: ::::::::::: ::::::::::: :: ::::: :::::
_      ACCACGACGCTACTCAGACTACCCAGATGCTTACACCACATGAAACACTGTCTCTTCTAT
         6640      6650      6660      6670      6680      6690

        6690      6700      6710      6720      6730      6740
974467 AGGCTCATTCATCTCACTTACGGCCGTCCTTGTAATGATCTTCATGATTTGAGAAGCCTT
       ::: ::::: :: ::::: :: :: :: ::  : :::::::: :: :::::::: :::::
_      AGGATCATTTATTTCACTAACAGCTGTTCTCATCATGATCTTTATAATTTGAGAGGCCTT
         6700      6710      6720      6730      6740      6750

        6750      6760      6770      6780      6790      6800
974467 CGCATCAAAACGAGAAGTGCTCTCAATTTCCTACTCTTCAACTAACCTAGAATGACTGCA
        :: ::::::::::::::  : ::: : :: ::  ::::::: ::  :::::::::: ::
_      TGCTTCAAAACGAGAAGTAATATCAGTATCGTATGCTTCAACAAATTTAGAATGACTTCA
         6760      6770      6780      6790      6800      6810

        6810      6820      6830      6840      6850      6860
974467 TGGATGCCCCCCACCTTACCACACATTCGAAGAACCTTCCTATGTAAAAGTTAAATAAGA
       ::: ::::: ::::: :: ::::::::::: :::::  ::::::::::::: ::::::::
_      TGGCTGCCCTCCACCATATCACACATTCGAGGAACCAACCTATGTAAAAGTAAAATAAGA
         6820      6830      6840      6850      6860      6870

        6870      6880      6890      6900      6910      6920
974467 AAGGAAGGATTCGAACCCCCTACAACTGGTTTCAAGCCAATTTCATAACCATTATGTCTT
       ::::::::: :::::::::::: :: ::::::::::::::: ::::: ::  ::::::::
_      AAGGAAGGAATCGAACCCCCTAAAATTGGTTTCAAGCCAATCTCATATCCTATATGTCTT
         6880      6890      6900      6910      6920      6930

        6930      6940      6950      6960      6970      6980
974467 TCTCAATGAGATATTAGTAAAAT-AATTACATAACCTTGTCAAGGTTAAGTTATAGACTT
       ::::::: ::::::::::::::: ::::::::::: ::::::: ::::: :::::::
_      TCTCAATAAGATATTAGTAAAATCAATTACATAACTTTGTCAAAGTTAAATTATAGATCA
         6940      6950      6960      6970      6980      6990

          6990      7000      7010      7020      7030      7040
974467 A-AATCTATATATCTTACATGGCTTACCCATTTCAACTTGGCTTACAAGACGCCACATCA
       : ::::::::::::::: ::::: :::::::: ::::::::  ::::::::::::::::
_      ATAATCTATATATCTTATATGGCCTACCCATTCCAACTTGGTCTACAAGACGCCACATCC
         7000      7010      7020      7030      7040      7050

          7050      7060      7070      7080      7090      7100
974467 CCTATCATAGAAGAACTTACAAACTTTCATGACCACACCCTAATAATTGTATTCCTCATC
       ::::: :::::::: :: : ::: :: ::::: ::::: ::::::::::: ::::: ::
_      CCTATTATAGAAGAGCTAATAAATTTCCATGATCACACACTAATAATTGTTTTCCTAATT
         7060      7070      7080      7090      7100      7110
```

```
          7110      7120      7130      7140      7150      7160
974467 AGCTCCCTAGTACTTTATATTATTTCACTAATACTAACAACAAAACTAACACACACAAGC
       :::::: :::: :: ::::: :: :: :::::: ::::::::::::::::::: ::::::
_      AGCTCCTTAGTCCTCTATATCATCTCGCTAATATTAACAACAAAACTAACACATACAAGC
         7120      7130      7140      7150      7160      7170

          7170      7180      7190      7200      7210      7220
974467 ACAATAGACGCCCAAGAAGTAGAAACAATTTGAACAATTCTCCCAGCTGTCATTCTTATT
       :::::::: :: :::::::: ::::: :::::::: ::::: :::::::: :: ::::: 
_      ACAATAGATGCACAAGAAGTTGAAACCATTTGAACTATTCTACCAGCTGTAATCCTTATC
         7180      7190      7200      7210      7220      7230

          7230      7240      7250      7260      7270      7280
974467 CTAATTGCCCTTCCCTCCCTACGAATTCTATACATAATAGACGAGATTAATAACCCAGTT
        ::::::: :: ::::: ::::: :::::::: ::::::::::: :: :: ::::: :: 
_      ATAATTGCTCTCCCCTCTCTACGCATTCTATATATAATAGACGAAATCAACAACCCCGTA
         7240      7250      7260      7270      7280      7290

          7290      7300      7310      7320      7330      7340
974467 CTAACAGTAAAAACTATAGGACACCAATGATACTGAAGCTATGAATATACTGACTATGAA
        :::: :: ::::: ::::: :::::::::::::::::::: ::::::::::::::::::
_      TTAACCGTTAAAACCATAGGGCACCAATGATACTGAAGCTACGAATATACTGACTATGAA
         7300      7310      7320      7330      7340      7350

          7350      7360      7370      7380      7390      7400
974467 GACCTATGCTTTGACTCCTACATAATCCCAACCAATGACCTAAAACCAGGTGAACTTCGT
       :::::::::::::: :: :: ::::::::::: :: ::::::::::: :::::::: :: 
_      GACCTATGCTTTGATTCATATATAATCCCAACAAACGACCTAAAACCTGGTGAACTACGA
         7360      7370      7380      7390      7400      7410

          7410      7420      7430      7440      7450      7460
974467 CTATTAGAAGTTGATAATCGGGTAGTCTTACCAATAGAACTTCCAATTCGTATACTAATC
       ::  ::::::::::::: :: :: ::  : ::::::::::::::::: :::::: :::: 
_      CTGCTAGAAGTTGATAACCGAGTCGTTCTGCCAATAGAACTTCCAATCCGTATATTAATT
         7420      7430      7440      7450      7460      7470

          7470      7480      7490      7500      7510      7520
974467 TCATCCGAAGACGTCCTGCACTCATGAGCCATCCCTTCACTAGGGTTAAAAACCGACGCA
       ::::: ::::::::::: :::::::::::  :::: :: :::::  : ::::: :: :: 
_      TCATCTGAAGACGTCCTCCACTCATGAGCAGTCCCCTCCCTAGGACTTAAAACTGATGCC
         7480      7490      7500      7510      7520      7530

          7530      7540      7550      7560      7570      7580
974467 ATCCCCGGCCGCCTAAACCAAGCTACAGTCACATCAAACCGACCAGGTCTATTCTATGGC
       ::::: ::::: ::::: ::::: ::::: :::::::::::::::::  :::::::::::
_      ATCCCAGGCCGACTAAATCAAGCAACAGTAACATCAAACCGACCAGGGTTATTCTATGGC
         7540      7550      7560      7570      7580      7590

          7590      7600      7610      7620      7630      7640
974467 CAATGCTCTGAAATTTGCGGCTCAAATCACAGCTTCATACCCATTGTACTAGAAATAGTG
       ::::::::::::::::: :: :: :: :: ::::: :: :::::::: :::::::: :: 
_      CAATGCTCTGAAATTTGTGGATCTAACCATAGCTTTATGCCCATTGTCCTAGAAATGGTT
         7600      7610      7620      7630      7640      7650

          7650      7660      7670      7680      7690      7700
974467 CCTCTAAAATATTTCGAAAACTGATCAGCTTCTATAATTTAAACTCATTGCGAAGCTTAG
       :: ::::::::::::::::::::::: ::::: :::::::::  ::: :  :::::: ::
_      CCACTAAAATATTTCGAAAACTGATCTGCTTCAATAATTTAATTTCACTATGAAGCTAAG
         7660      7670      7680      7690      7700      7710

          7710      7720      7730      7740       7750      7760
974467 AGCGTTAACCTTTTAAGTTAAAGTTAGAGACAACAAA-TCTCCACAATGACATGCCACAA
       :::::::::::::::::::::::::::::::   ::: :::::: : ::: :::::::::
_      AGCGTTAACCTTTTAAGTTAAAGTTAGAGACCTTAAAATCTCCATAGTGATATGCCACAA
         7720      7730      7740      7750      7760      7770

           7770      7780      7790      7800      7810      7820
974467 CTAGACACATCCACATGATTTATTACAATCATCTCCTCAATAGCCACACTATTTATTTTA
       ::::: ::::: ::::::::::: ::::: ::::: ::::::   :: :::::::: :::
_      CTAGATACATCAACATGATTTATCACAATTATCTCATCAATAATTACCCTATTTATCTTA
         7780      7790      7800      7810      7820      7830

           7830      7840      7850      7860      7870      7880
974467 TTTCAATTAAAAATTTCTTCCCAAACCTTTCCTGCACCTCCCTCACCCAAAACTATAGCC
       :::::: ::::: : :: :: ::::: :: ::     : :: ::::: ::: :  :: : 
_      TTTCAACTAAAAGTCTCATCACAAACATTCCCACTGGCACCTTCACCAAAATCACTAACA
         7840      7850      7860      7870      7880      7890

           7890      7900      7910      7920      7930      7940
974467 ACAGAAAAAACGAATAACCCTTGAGAATCAAAATGAACGAAAATCTATTTGCCTCTTTCA
       ::   ::::   :: : ::::::::::: :::::::::::::::::::::::::: ::::
_      ACCATAAAAGTAAAAACCCCTTGAGAATTAAAATGAACGAAAATCTATTTGCCTCATTCA
         7900      7910      7920      7930      7940      7950

           7950      7960      7970      7980      7990      8000
974467 TTACCCCCACAATAATAGGTCTACCAATTGTTGTGACCATTATTATGTTCCCATCAATTC
       ::::::: :::::::::::  : ::::: :::::  :::: ::::: :: :: ::::: :
_      TTACCCCAACAATAATAGGATTCCCAATCGTTGTAGCCATCATTATATTTCCTTCAATCC
         7960      7970      7980      7990      8000      8010

           8010      8020      8030      8040      8050      8060
974467 TATTCCCATCATCAGAACGCCTAATCAGCAACCGACTACACTCATTTCAACACTGACTAA
       :::::::::: ::: :::::::::::: :::::: :: :: :: :: :::::::::::: 
```

```
_       TATTCCCATCCTCAAAACGCCTAATCAACAACCGTCTCCATTCTTTCCAACACTGACTAG
          8020      8030      8040      8050      8060      8070

            8070      8080      8090      8100      8110      8120
974467  TCAAACTTATCATCAAACAAATAATGTTAATCCACACACCAAAAGGACGAACCTGAGCCC
        : :::::::: ::::::::::::::: ::::::::::::::::::::::::: ::: :::
_       TTAAACTTATTATCAAACAAATAATGCTAATCCACACACCAAAAGGACGAACATGAACCC
          8080      8090      8100      8110      8120      8130

            8130      8140      8150      8160      8170      8180
974467  TAATAATTGTATCCCTAATTATATTTATCGGCTCAACCAACCTTCTAGGGCTTCTTCCCC
        :::::::::: :::::::: :::::::: :: ::::: :: :: ::::: ::: : :: :
_       TAATAATTGTTTCCCTAATCATATTTATTGGATCAACAAATCTCCTAGGCCTTTTACCAC
          8140      8150      8160      8170      8180      8190

            8190      8200      8210      8220      8230      8240
974467  ATACATTTACCCCTACCACTCAGCTATCTATAAACCTAAGCATAGCCATCCCCCTATGAG
        :::::::::: ::::: :: :: ::::: ::::: ::::: :::::::: :: :::::::
_       ATACATTTACACCTACTACCCAACTATCCATAAATCTAAGTATAGCCATTCCACTATGAG
          8200      8210      8220      8230      8240      8250

            8250      8260      8270      8280      8290      8300
974467  CAGGAGCCGTAATTCTAGGCTTCCGACACAAACTAAAAAATTCTTTAGCCCACTTCTTAC
        : ::::::::::::  :::::::::::::::::::::::  ::  : ::::::::: : :
_       CTGGAGCCGTAATTACAGGCTTCCGACACAAACTAAAAAGCTCACTTGCCCACTTCCTTC
          8260      8270      8280      8290      8300      8310

            8310      8320      8330      8340      8350      8360
974467  CGCAAGGAACCCCCATCTCACTAATTCCCATACTAATCATCATCGAAACTATCAGCCTAT
        : :::::::: :: :: ::::::::::: ::::: :: :: :: ::::: :: :::::::
_       CACAAGGAACTCCAATTTCACTAATTCCAATACTTATTATTATTGAAACAATTAGCCTAT
          8320      8330      8340      8350      8360      8370

            8370      8380      8390      8400      8410      8420
974467  TTATTCAACCGATAGCACTAGCAGTACGACTAACAGCAAACATTACAGCAGGCCATCTAT
        :::::::::: :: ::: ::::::: :: :: ::::: :::::::: ::::: ::  :::
_       TTATTCAACCAATGGCATTAGCAGTCCGGCTTACAGCTAACATTACTGCAGGACACTTAT
          8380      8390      8400      8410      8420      8430

            8430      8440      8450      8460      8470      8480
974467  TAATGCATCTAATCGGAGGAGCTACCCTAGTACTTATAGACATCAGCCCACCAACCGCTA
        :::: :: ::::::::::::::::: :::::: : ::: : :: ::::::::::: ::::
_       TAATACACCTAATCGGAGGAGCTACTCTAGTATTAATAAATATTAGCCCACCAACAGCTA
          8440      8450      8460      8470      8480      8490

            8490      8500      8510      8520      8530      8540
974467  CAATTACATTTATTATTCTACTTCTACTTACAGTACTTGAATTTGCCGTAGCCTTAATTC
        : ::::::::::::::: :::::::::: ::: : :: :::::::: ::::: :::::::
_       CCATTACATTTATTATTTTACTTCTACTCACAATTCTAGAATTTGCAGTAGCATTAATTC
          8500      8510      8520      8530      8540      8550

            8550      8560      8570      8580      8590      8600
974467  AAGCCTATGTATTCACCCTTCTAGTAAGCCTGTACCTACATGATAACACATAATGACCCA
        ::::::: ::::::::::: ::::::::::: :: ::::::::::: :::::::::::::
_       AAGCCTACGTATTCACCCTCCTAGTAAGCCTATATCTACATGATAATACATAATGACCCA
          8560      8570      8580      8590      8600      8610

            8610      8620      8630      8640      8650      8660
974467  CCAAACCCATGCATACCATATAGTAAACCCAAGCCCATGACCACTAACAGGAGCCCTATC
        :::::: :::::::: :: ::::: :: ::::: ::::::::: :::: :::::: : ::
_       CCAAACTCATGCATATCACATAGTTAATCCAAGTCCATGACCATTAACTGGAGCCTTTTC
          8620      8630      8640      8650      8660      8670

            8670      8680      8690      8700      8710      8720
974467  AGCTCTTCTACTCACATCCGGCTTAGTAATATGATTCCATTACAACTCCACAATTCTCCT
        ::: :: :: :: ::::: ::  ::::::::::::: :: :: :: :: :  :  ::  :
_       AGCCCTCCTTCTAACATCAGGTCTAGTAATATGATTTCACTATAATTCAATTACACTATT
          8680      8690      8700      8710      8720      8730

            8730      8740      8750      8760      8770      8780
974467  ATCATTAGGCCTCCTGACAAACATCCTAACTATATATCAATGATGACGAGATATCATCCG
        : :  : ::::: :: :: :: ::::: :: ::::::::::::::::::::  : :: ::
_       AACCCTTGGCCTACTCACCAATATCCTCACAATATATCAATGATGACGAGACGTAATTCG
          8740      8750      8760      8770      8780      8790

            8790      8800      8810      8820      8830      8840
974467  TGAAGGAACATACCAAGGCCACCACACCCCTATTGTACAAAAAGGCCTCCGATACGGAAT
        ::::::::: ::::::::::::::::: ::::::::::::::::: :: ::::: :: ::
_       TGAAGGAACCTACCAAGGCCACCACACTCCTATTGTACAAAAAGGACTACGATATGGTAT
          8800      8810      8820      8830      8840      8850

            8850      8860      8870      8880      8890      8900
974467  AATCCTGTTTATTGTCTCCGAAGTATTCTTCTTTGCCGGATTTTTCTGAGCATTTTATCA
        ::: :: :: :: ::::: :::::::: :::::::: ::::: :::::::: :: :::::
_       AATTCTATTCATCGTCTCGGAAGTATTTTTCTTTGCAGGATTCTTCTGAGCGTTCTATCA
          8860      8870      8880      8890      8900      8910

            8910      8920      8930      8940      8950      8960
974467  TTCCAGCCTAGTTCCTACCCACGACCTAGGCGGTTGCTGACCCCCAACAGGAATCACCCC
        ::: ::::: :: :: :: :: :: ::::: :: :::::::: :::::::::::  : ::
_       TTCTAGCCTCGTACCAACACATGATCTAGGAGGCTGCTGACCTCCAACAGGAATTTCACC
          8920      8930      8940      8950      8960      8970
```

```
            8970      8980      8990      9000      9010      9020
974467 TTTAAATCCCCTAGAAGTACCCCTTCTAAATACATCAGTCCTCTTAGCATCAGGAGTCTC
         : :: :: :::::::: :: :: :: ::::: ::::: ::  :::::::::: :: ::
_      ACTTAACCCTCTAGAAGTCCCACTACTTAATACTTCAGTACTTCTAGCATCAGGTGTTTC
          8980      8990      9000      9010      9020      9030

            9030      9040      9050      9060      9070      9080
974467 AATTACATGAGCCCATCACAGCCTAATAGAAGGCAACCGAAACCATATAAACCAAGCCCT
       ::::::::::: ::::: ::::: :::::::: :: :::::::: ::::: ::::::::
_      AATTACATGAGCTCATCATAGCCTTATAGAAGGTAAACGAAACCACATAAATCAAGCCCT
          9040      9050      9060      9070      9080      9090

            9090      9100      9110      9120      9130      9140
974467 ACTAATCACCATTCTCTTAGGATTATATTTCACTATCTTACAAGCCTCAGAGTATTTCGA
       :::::: :::::: :  ::::: : :: ::::: ::: : ::::: ::::: :: :: ::
_      ACTAATTACCATTATACTAGGACTTTACTTCACCATCCTCCAAGCTTCAGAATACTTTGA
          9100      9110      9120      9130      9140      9150

            9150      9160      9170      9180      9190      9200
974467 AACATCATTTTCTATCTCAGACGGAATTTACGGCTCAACATTCTTCATAGCAACGGGATT
       :::::::: :: :: ::::: :: :: :: :: :: :::::::::: :: :: :::::
_      AACATCATTCTCCATTTCAGATGGTATCTATGGTTCTACATTCTTCATGGCTACTGGATT
          9160      9170      9180      9190      9200      9210

            9210      9220      9230      9240      9250      9260
974467 TCATGGCCTCCACGTAATTATTGGCTCAACTTTCCTAATTGTCTGTCTACTACGACAACT
        ::::: ::::: :::::::::: ::::: ::::: ::::: :: :::::::::::::
_      CCATGGACTCCATGTAATTATTGGATCAACATTCCTTATTGTTTGCCTACTACGACAACT
          9220      9230      9240      9250      9260      9270

            9270      9280      9290      9300      9310      9320
974467 AAAATTCCACTTCACATCAAAACATCATTTCGGATTTGAAGCCGCAGCATGATACTGACA
       :::::: ::::::::::::::::::: :::::::::::::::::::::::::::::
_      AAAATTTCACTTCACATCAAAACATCACTTCGGATTTGAAGCCGCAGCATGATACTGACA
          9280      9290      9300      9310      9320      9330

            9330      9340      9350      9360      9370      9380
974467 CTTCGTAGATGTAGTTTGACTATTCCTATACGTTTCTATCTATTGATGAGGATCCTACTC
        :: ::::: :::: ::::: :::::::::: :: :: ::::::::::::: :::::
_      TTTTGTAGACGTAGTCTGACTTTTCCTATACGTCTCCATTTATTGATGAGGATCTTACTC
          9340      9350      9360      9370      9380      9390

            9390      9400      9410      9420      9430      9440
974467 CCTTAGTATAAACAATACAACTGACTTCCAATCAGTTAATTCTGAAAAAACTCAGAAGAG
       ::::::::::  :::: ::::::::::::: :::  :::::::: :::: ::::::::
_      CCTTAGTATAATTAATATAACTGACTTCCAATTAGTAGATTCTGAATAAACCCAGAAGAG
          9400      9410      9420      9430      9440      9450

            9450      9460      9470      9480      9490      9500
974467 AGTAATTAACCTACTTATTATCATCACAATTAACATCACCTTATCTTTTATCCTCATTTC
       ::::::::::::    : : : :::   ::::: ::    :::::  : :  :: :::
_      AGTAATTAACCTGTACACTGTTATCTTCATTAATATTTATTATATCCCTAACGCTAATTCT
          9460      9470      9480      9490      9500      9510

            9510      9520      9530      9540      9550      9560
974467 AATTGCATTCTGATTGCCTCAAATAAACTTATACTCCGAAAAAGCAAACCCATATGAATG
       : :::::::::: : :: ::::::::  : ::::: :::::::::: ::::::::::
_      AGTTGCATTCTGACTCCCCCAAATAAATCTGTACTCAGAAAAAGCAAATCCATATGAATG
          9520      9530      9540      9550      9560      9570

            9570      9580      9590      9600      9610      9620
974467 TGGCTTCGACCCAACAAGTTCTGCACGCCTTCCTTTTTCAATAAAATTTTTCTTAGTAGC
        :: :::::::: ::::: :::::::: :: :: :: :::::::::::::::: :::::
_      CGGATTCGACCCTACAAGCTCTGCACGTCTACCATTCTCAATAAAATTTTTCTTGGTAGC
          9580      9590      9600      9610      9620      9630

            9630      9640      9650      9660      9670      9680
974467 CATTACATTTCTACTATTCGACCTAGAAATCGCCTTACTACTTCCCCTCCCATGAGCGAT
        ::::::::::: :::: :::::::::: ::  : ::::::: :: :::::::: ::
_      AATTACATTTCTATTATTTGACCTAGAAATTGCTCTTCTACTTCCACTACCATGAGCAAT
          9640      9650      9660      9670      9680      9690

            9690      9700      9710      9720      9730      9740
974467 TCAAACAACCAATACCACTACAATAATAGCAACTGCCTTTATTTAGTCACTATTTTAGC
       :::::::  :: ::: :::: ::::::   :  ::::::::: ::::::: ::: :: :
_      TCAAACAATTAAAACCTCTACTATAATAATTATAGCCTTTATTCTAGTCACAATTCTATC
          9700      9710      9720      9730      9740      9750

            9750      9760      9770      9780      9790      9800
974467 TCTTGGCCTAAGCTACGAATGAACACAAAAAGGACTAGAATGAACAGAATAATTGGTAAT
       ::: ::::::   :: ::::::::::::::::: ::::::::::::: ::: :::::::
_      TCTAGGCCTAGCATATGAATGAACACAAAAAGGATTAGAATGAACAGAGTAAATGGTAAT
          9760      9770      9780      9790      9800      9810

            9810      9820      9830      9840      9850      9860
974467 TAGTTTAAATAAAATTAATGATTTCGACTCATTAGATTATGATAATAATCATAATTACCA
       ::::::::: ::::::::::::::::::::::::::::::::: ::  :::::::::::
_      TAGTTTAAAAAAAAATTAATGATTTCGACTCATTAGATTATGATGATGTTCATAATTACCA
          9820      9830      9840      9850      9860      9870

            9870      9880      9890      9900      9910      9920
974467 ACAATGACATCTGCTTTCCTAAATTTAACTATAGCCTTTACATTATCTCTACTAGGTACT
       : : :: ::::: : ::: : ::  : :: ::::::::  :: :::: :: ::::: ::
```

```
_      ATA-TGCCATCTACCTTCTTCAACCTCACCATAGCCTTCTCACTATCACTTCTAGGGACA
          9880      9890      9900      9910      9920      9930

           9930      9940      9950      9960      9970      9980
974467 TTTATATTTCGCTCCCACTTAATATCTACTCTCCCTCTGCCTAGAAGGAATAATACTATCA
        ::::::::::: ::: ::::::: ::  : :: ::::: ::::: ::: :: ::::
_      CTTATATTTCGCTCTCACCTAATATCCACATTACTATGCCTGGAAGGCATAGTATTATCC
          9940      9950      9960      9970      9980      9990

           9990     10000     10010     10020     10030     10040
974467 CTATTTGTCATAACTTCAACATCCACATTAAACTCCAACTCCATAATCTCCATAACCATC
        ::::: : ::::::::  : :  :  :::::::::::::::: ::::::: : :::
_      TTATTTATTATAACTTCAGTAACTTCCCTAAACTCCAACTCCATAAGCTCCATACCAATC
         10000     10010     10020     10030     10040     10050

          10050     10060     10070     10080     10090     10100
974467 CCAATTACCATTCTAGTTTTTGCAGCCTGCGAAGCAGCAGTAGGTTTAGCCTTACTAGTA
       :: :: :::::  ::::::: :::::::::::::::: :::::  ::::: ::::::::
_      CCCATCACCATCTTAGTTTTCGCAGCCTGCGAAGCAGCTGTAGGACTAGCCCTACTAGTA
         10060     10070     10080     10090     10100     10110

          10110     10120     10130     10140     10150     10160
974467 AAAATTTCAAATACTTACGGAACAGACTACGTACAAAACCTCAACCTTCTACAATGTTAA
       ::: ::::::: :: ::::::::::: ::::: ::::: :::::::: :::::::: :::
_      AAAGTTTCAAACACGTACGGAACAGATTACGTCCAAAATCTCAACCTACTACAATGCTAA
         10120     10130     10140     10150     10160     10170

          10170     10180     10190     10200     10210     10220
974467 AAATTATTTTCCCATCTATCATACTCCTCCCACTAACATGACTCTCAGCCAACAAAAAAA
       :::::::: : :: ::  : :: :: :: :::::::: ::::: :::  :   :::::::
_      AAATTATTCTTCCCTCACTAATGCTACTACCACTAACCTGACTATCAAGCCCTAAAAAAA
         10180     10190     10200     10210     10220     10230

          10230     10240     10250     10260     10270     10280
974467 TCTGAACCAATGTCACCTCCTACAGCTTTCTAGTGAGCCTATTAAGCTTATCACTCCTAT
        ::::: :: :: ::::: :: :: :::::: : ::  ::   ::: :: :::: ::::
_      CCTGAACAAACGTAACCTCATATAGTTTTCTAATTAGTTTAACCAGCCTAACACTTCTAT
         10240     10250     10260     10270     10280     10290

          10290     10300     10310     10320     10330     10340
974467 GACAAAATGACGAAAATTACCTAAATTTCTCAGTTATATTCTCCTCCGATCCATTATCCA
       ::::::  ::::::::::   ::: :: :::  ::::::::::: :: ::  ::::::
_      GACAAACCGACGAAAATTATAAAAACTTTCAAATATATTCTCCTCAGACCCCCTATCCA
         10300     10310     10320     10330     10340     10350

          10350     10360     10370     10380     10390     10400
974467 CCCCACTAATCATTCTAACAACTTGACTCCTCCCACTAATAATGCTCGCTAGCCAAAATC
       : ::: :::: ::: ::::: : ::: : :: ::::::::: :  : ::::::::::: :
_      CACCATTAATTATTTTAACAGCCTGATTACTGCCACTAATATTAATAGCTAGCCAAAACC
         10360     10370     10380     10390     10400     10410

          10410     10420     10430     10440     10450     10460
974467 ACATAAAAAAAGAAAATATAATGCATCAAAAACTTTACATCTCAATACTTATTAGCCTCC
       :: :::::::::: ::::   : :  :::::::: ::::::::::::: :: ::: : :
_      ACCTAAAAAAAGATAATAACGTACTACAAAAACTCTACATCTCAATACTAATCAGCTTAC
         10420     10430     10440     10450     10460     10470

          10470     10480     10490     10500     10510     10520
974467 AAATTTTACTCATCATAACATTCTCCGCAACAGAACTAATTTTATTTTATATCCTGTTCG
       ::::: : :: :::::::: :: :: ::::: ::::::::: ::::::::::  : :: :
_      AAATTCTCCTAATCATAACCTTTTCAGCAACTGAACTAATTATATTTTATATTTTATTTG
         10480     10490     10500     10510     10520     10530

          10530     10540     10550     10560     10570     10580
974467 AAGCCACTCTAATCCCAACACTAATTATCATTACACGATGAGGCAACCAAACAGAACGCT
       :::: ::  ::::::::::::: ::::: ::::: :::::::: :::::::: ::::::
_      AAGCAACCTTAATCCCAACACTTATTATTATTACCCGATGAGGGAACCAAACTGAACGCC
         10540     10550     10560     10570     10580     10590

          10590     10600     10610     10620     10630     10640
974467 TAAATGCAGGAATTTATTTCCTGTTTTATACACTAATTGGCTCCATCCCACTCTTAATTG
       :::: ::::: :::::::::: ::::::: ::::: :: :: :: :::::  ::::::
_      TAAACGCAGGGATTTATTTCCTATTTTATACCCTAATCGGTTCTATTCCACTGCTAATTG
         10600     10610     10620     10630     10640     10650

          10650     10660     10670     10680     10690     10700
974467 CCCTCATTTCAATCCAAAACTCAATAGGAACACTCAACTTCCTAATCCTTTCCCTCACAA
       ::::::: : ::::::::::    ::::::: :: ::: :: ::::  : ::  ::::::
_      CCCTCATCTTAATCCAAAACCATGTAGGAACCCTAAACCTCATAATTTTATCATTCACAA
         10660     10670     10680     10690     10700     10710

          10710     10720     10730     10740     10750     10760
974467 CACACCCCCTACCCTCAACATGATCCAACACCATTCTATGACTAGCATGTATAATAGCAT
       ::::: :: ::  : :  ::::::: :::: : : :::::  : ::::: ::::::::::
_      CACACACCTTAGACGCTTCATGATCTAACAACTTACTATGGTTGGCATGCATAATAGCAT
         10720     10730     10740     10750     10760     10770

          10770     10780     10790     10800     10810     10820
974467 TTATAATCAAAATACCATTATACGGAGTCCATCTATGATTACCAAAAGCCCACGTAGAAG
       :: : :: ::::::::::::: ::::: :: :::::: :::::::::::: :: ::::
_      TTCTTATTAAAATACCATTATATGGAGTTCACCTATGACTACCAAAAGCCCATGTTGAAG
         10780     10790     10800     10810     10820     10830
```

```
          10830     10840     10850     10860     10870     10880
974467 CTCCAATTGCAGGCTCTATAATTTTAGCAGCAATTCTCCTAAAGCTAGGGGGTTATGGGA
       :::::::::: :: :: :::::: ::::::: ::::: :::::  ::::  :::: :: :
_      CTCCAATTGCTGGGTCAATAATTCTAGCAGCTATTCTTCTAAAATTAGGTAGTTACGGAA
        10840     10850     10860     10870     10880     10890

          10890     10900     10910     10920     10930     10940
974467 TAATACGAGTTTCCATCATTCTAGACCCCCTAACAAAATCTTTAGCCTACCCATTCATCA
       :::: ::  : ::::: ::::::::::: :::::::::: : :::: ::::: ::::::
_      TAATTCGCATCTCCATTATTCTAGACCCACTAACAAAATATATAGCATACCCCTTCATCC
        10900     10910     10920     10930     10940     10950

          10950     10960     10970     10980     10990     11000
974467 TCCTCTCATTATGAGGCATAATTATAACTAGCTCAATCTGCCTACGCCAAACAGATCTAA
       : :::::  ::::::: :::::::::::::::::::::::: :::::::::::::: :::
_      TTCTCTCCCTATGAGGAATAATTATAACTAGCTCAATCTGCTTACGCCAAACAGATTTAA
        10960     10970     10980     10990     11000     11010

          11010     11020     11030     11040     11050     11060
974467 AATCATTAATTGCTTACTCATCAGTAAGCCATATAGCCCTAGTCATCACAGCCATTATAA
       ::::: :::: :: ::::: ::::: ::::: ::::: :: :: ::  :: : :: ::::
_      AATCACTAATCGCCTACTCCTCAGTTAGCCACATAGCACTTGTTATTGCATCAATCATAA
        11020     11030     11040     11050     11060     11070

          11070     11080     11090     11100     11110     11120
974467 TCCAGACACCATGAAGCTTCATGGGAGCTACAATACTAATAATCGCCCACGGCTTAACCT
       :::: :: :::::::::::::: ::::: ::::::::::::::::::: :: ::: : :: :
_      TCCAAACTCCATGAAGCTTCATAGGAGCAACAATACTAATAATCGCACATGGCCTCACAT
        11080     11090     11100     11110     11120     11130

          11130     11140     11150     11160     11170     11180
974467 CATCACTCTTATTCTGCCTAGCAAACACCAACTACGAACGAATTCACAGCCGAACTATAA
       :::::::: :::::::::::::::::: ::::::::::::: :: :::::::: :::::::
_      CATCACTCCTATTCTGCCTAGCAAACTCCAACTACGAACGGATCCACAGCCGTACTATAA
        11140     11150     11160     11170     11180     11190

          11190     11200     11210     11220     11230     11240
974467 TTATAGCTCGAGGATTACAAATAATCTTTCCATTGATAGCAACATGATGACTATTAGCAA
       : :: :: :::::: : :::::  :::: ::: : ::::: :::::::::::  ::::::
_      TCATGGCCCGAGGACTTCAAATGGTCTTCCCACTTATAGCCACATGATGACTGATAGCAA
        11200     11210     11220     11230     11240     11250

          11250     11260     11270     11280     11290     11300
974467 GCTTAGCCAACCTAGCACTACCACCCCTAATTAACCTCATAGGCGAGTTATTCATTGTTA
       :  :::: :: ::::: ::::: ::   ::: :: :: ::::: :: :::::::::   :
_      GTCTAGCTAATCTAGCTCTACCCCCTTCAATCAATCTAATAGGAGAATTATTCATTACCA
        11260     11270     11280     11290     11300     11310

          11310     11320     11330     11340     11350     11360
974467 TAGCAACATTTTCCTGATCGAACCCCTCTATCATCCTTATAGCAACTAACATTGTCATCA
       :: ::  :::::: ::::: :::    : :: :: ::::::: :: ::::::: : :: :
_      TATCATTATTTTCTTGATCAAACTTTACCATTATTCTTATAGGAATTAACATTATTATTA
        11320     11330     11340     11350     11360     11370

          11370     11380     11390     11400     11410     11420
974467 CAGGAATATACTCAATATATATGATTATCACAACCCAACGAGGAAAACTAACCAGCCACA
       :::: :::::::::::::: :: ::::: :: :::::::: :: ::::::::::: ::: :
_      CAGGTATATACTCAATATACATAATTATTACCACCCAACGCGGCAAACTAACCAACCATA
        11380     11390     11400     11410     11420     11430

          11430     11440     11450     11460     11470     11480
974467 TAAACAACCTCCAACCTTCCCACACACGAGAATTAACACTCATAGCTCTACACATTATTC
       :::  ::::::::::: :: :::::::::::: :::::::: ::::: :: ::::: ::::
_      TAATTAACCTCCAACCCTCACACACACGAGAACTAACACTAATAGCCCTTCACATAATTC
        11440     11450     11460     11470     11480     11490

          11490     11500     11510     11520     11530     11540
974467 CCCTCATACTATTAACAATCAACCCTAAACTCATCACAGGCCTAACAATATGTAGATATA
       : :: :: ::  :::: : ::  :: ::::: :: :::::::: :::::::::  :::::
_      CACTTATTCTTCTAACTACCAGTCCAAAACTAATTACAGGCCTGACAATATGTGAATATA
        11500     11510     11520     11530     11540     11550

          11550     11560     11570     11580     11590     11600
974467 GTTTACAAAAAACATTAGACTGTGAATCTAACAACAGGAAATCAAATTCCTTATTTACCA
       ::::::::::::::::::::::::::::: :::::::::::: ::  :::::::: ::::
_      GTTTACAAAAAACATTAGACTGTGAATCTGACAACAGGAAATAAACCTCCTTATTCACCA
        11560     11570     11580     11590     11600     11610

          11610     11620     11630     11640     11650     11660
974467 AGAAAGTATGCAAGAACTGCTAATTCATGCACCCATACCTAAAACATATGGCTTTCTTAC
       ::::::  ::::::::::::::::::::::  ::::   ::::: : :::::::::::::
_      AGAAAGATTGCAAGAACTGCTAATTCATGCTTCCATGTTTAAAA-ACATGGCTTTCTTAC
        11620     11630     11640     11650     11660     11670

          11670     11680     11690     11700     11710     11720
974467 TTTTATAGGATAGAAGTAATCCATTGGTCTTAGGAACCAAAAACCTTGGTGCAACTCCAA
       ::::::::::::  :::::::::::::::::::::::::::::::::::::::: :::::
_      TTTTATAGGATAATAGTAATCCATTGGTCTTAGGAACCAAAAACCTTGGTGCAAATCCAA
          11680     11690     11700     11710     11720     11730

          11730     11740     11750     11760     11770     11780
974467 ATAAAAGTAATAAATATAATAACATCCTCAATCCTCATAATCTTGATTTTACTTACAACA
       ::::::::::: :::::  : ::: :::::::: :  ::::::: ::: : ::   : :
```

```
_       ATAAAAGTAATCAATATTTTCACAACCTCAATCTTATTAATCTTCATTCTTCTACTATCC
          11740     11750     11760     11770     11780     11790

          11790     11800     11810     11820     11830     11840
974467 CCAATCATCATCTCCATAACCAACCTACCAAAACTTATTGACTTCCCGTCATATGCTACT
       :::::: : :: :: ::: : ::::::   ::::  ::  :::::::    ::  : :: 
_       CCAATCCTAATTTCAATATCAAACCTAATTAAACACATCAACTTCCCACTGTACACCACC
          11800     11810     11820     11830     11840     11850

          11850     11860     11870     11880     11890     11900
974467 TCATCAATTAAATTCTCATTCCTCCTTAGCCTCTTACCTTTACTACTATTCTTCCACCAC
        ::::::: :::::::: ::: :  :::::::::::::  :: :: :::: :::::: : 
_       ACATCAATCAAATTCTCCTTCATTATTAGCCTCTTACCCCTATTAATATTTTTCCACAAT
          11860     11870     11880     11890     11900     11910

          11910     11920     11930     11940     11950     11960
974467 AACACAGAATACATAATTACTAACTGACATTGACTAACTATTAATTCTATTAAACTTACT
       :: : :::::: :::::::: : ::: :: ::: : :: :: ::::: ::  ::::::  
_       AATATAGAATATATAATTACAACCTGGCACTGAGTCACCATAAATTCAATAGAACTTAAA
          11920     11930     11940     11950     11960     11970

          11970     11980     11990     12000     12010     12020
974467 ATAAGTTTCAAAATTGACTATTTCTCAATCCTATTCCTATCAGTAGCCCTATTCGTAACA
       ::::: ::::::: ::::: :::::: ::::: ::   ::: :::::::: :: :: :::
_       ATAAGCTTCAAAACTGACTTTTTCTCTATCCTGTTTACATCTGTAGCCCTTTTTGTCACA
          11980     11990     12000     12010     12020     12030

          12030     12040     12050     12060     12070     12080
974467 TGATCAATTATACAATTCTCTTCATGATATATACACTCTGACCCCCACATTAACCGATTC
       :::::::::::::::::::::::::::::::::::::: :::::  :::: :: ::::::
_       TGATCAATTATACAATTCTCTTCATGATATATACACTCAGACCCAAACATCAATCGATTC
          12040     12050     12060     12070     12080     12090

          12090     12100     12110     12120     12130     12140
974467 ATTAAGTACTTAATAATATTCCTAATTACTATACTAATTCTAACCTCAGCTAACAATCTA
       ::::: ::  : : : ::::::: ::::: :: :: :: :: :::::::: :::::  ::
_       ATTAAATATCTTACACTATTCCTGATTACCATGCTTATCCTCACCTCAGCCAACAACATA
          12100     12110     12120     12130     12140     12150

          12150     12160     12170     12180     12190     12200
974467 TTCCAACTCTTTATTGGATGAGAAGGGGTAGGAATTATATCCTTCTTATTAATCGGATGA
       :: ::::: :: ::::: ::::::::::: ::::::::::: ::: :: :::: ::::::
_       TTTCAACTTTTCATTGGCTGAGAAGGGGTGGGAATTATATCTTTCCTACTAATTGGATGA
          12160     12170     12180     12190     12200     12210

          12210     12220     12230     12240     12250     12260
974467 TGATATGGCCGTGCAGACGCCAACACAGCGGCCCTCCAAGCAATCCTCTACAACCGAGTC
       :: :: :: ::  ::::::: :: :: :: ::::: :::::::::::::: :::::  ::
_       TGGTACGGACGAACAGACGCAAATACTGCAGCCCTACAAGCAATCCTCTATAACCGCATC
          12220     12230     12240     12250     12260     12270

          12270     12280     12290     12300     12310     12320
974467 GGAGACATTGGTTTCATCCTAGCTATAACCTGATTCTGCCTAAACATAAACTCCTGAGAA
       :::::::: :: :::::  ::::::::   ::::: : ::::::::::::::: ::::::
_       GGAGACATCGGATTCATTTTAGCTATAGTTTGATTTTCCCTAAACATAAACTCATGAGAA
          12280     12290     12300     12310     12320     12330

          12330     12340     12350        12360     12370
974467 CTCCAACAAATTTTCTTAACCAACACCAAC---AATCTAGTCCCTCTCACAGGACTACTA
       :: ::::: ::: : ::  :::::: ::::   :::::: : :: :: : ::: ::: ::
_       CTTCAACAGATTATATTCTCCAACAACAACGACAATCTAATTCCACTTATAGGCCTATTA
          12340     12350     12360     12370     12380     12390

   12380     12390     12400     12410     12420     12430
974467 ATTGCAGCCACAGGAAAATCCGCCCAATTCGGACTTCATCCATGACTCCCATCAGCTATA
       :: ::::: ::::::::::: :: ::::: :: :: :: :::::::: :::::::: :::
_       ATCGCAGCTACAGGAAAATCAGCACAATTTGGCCTCCACCCATGACTACCATCAGCAATA
          12400     12410     12420     12430     12440     12450

   12440     12450     12460     12470     12480     12490
974467 GAAGGCCCAACTCCCGTCTCTGCCCTACTACACTCAAGCACAATAGTTGTGGCAGGGATC
       :::::::: :: :: :: :: :: :::::::::::::: :::::::: :: ::::: :: 
_       GAAGGCCCTACACCAGTTTCAGCACTACTACACTCAAGTACAATAGTAGTTGCAGGAATT
          12460     12470     12480     12490     12500     12510

   12500     12510     12520     12530     12540     12550
974467 TTCCTAATAATCCGATTCCACCCCCTAACCTCAAACAATAGTACTATTATAACAGCCATA
       :::::: :  :::::::::::::::: ::  : :: ::::    :::: ::::: : :::
_       TTCCTACTGGTCCGATTCCACCCCCTCACGACTAATAATAACTTTATTTTAACAACTATA
          12520     12530     12540     12550     12560     12570

   12560     12570     12580     12590     12600     12610
974467 CTATGTCTTGGTGCAATCACCACATTATTCACAGCAATCTGTGCTCTCACCCAAAACGAC
       :: :: :: :: ::  : ::::::::::: ::::: :: :::::::::::::::::::::
_       CTTTGCCTCGGAGCCCTAACCACATTATTTACAGCTATTTGTGCTCTCACCCAAAACGAC
          12580     12590     12600     12610     12620     12630

   12620     12630     12640     12650     12660     12670
974467 ATCAAAAAAATCGTAGCTTTCTCAACATCCAGCCAACTAGGCCTTATAATAGTCACATTA
       :::::::::::: : :: ::::: ::::: :::::::::::::: :::::::: ::  ::
_       ATCAAAAAAATCATTGCCTTCTCTACATCAAGCCAACTAGGCCTGATAATAGTGACGCTA
          12640     12650     12660     12670     12680     12690
```

```
   12680     12690     12700     12710     12720     12730
974467 GGGATTAACCAACCCTACCTTGCTTTCCTCCACATTTGCACCCATGCATTCTTCAAAGCC
       :: :: ::::::::  :::: :: ::::: ::::: :: ::::: :::::::::::::: 
_      GGAATAAACCAACCACACCTAGCATTCCTACACATCTGTACCCACGCATTCTTCAAAGCT
          12700     12710     12720     12730     12740     12750

   12740     12750     12760     12770     12780     12790
974467 ATATTATTCATATGCTCCGGATCAATCATCCATAGCCTCAACGATGAACAAGACATTCGA
       ::: : :: :::::::: :: :::::::: ::::::::    :: ::::::::::: :::
_      ATACTCTTTATATGCTCTGGCTCAATCATTCATAGCCTGGCAGACGAACAAGACATCCGA
          12760     12770     12780     12790     12800     12810

   12800     12810     12820     12830     12840     12850
974467 AAAATAGGCAATATAATAAAAGCAATACCATTCACATCATCTTGTCTCATTATCGGAAGC
       :::::::: :: :: : ::::   ::::::::::::::::: :: ::  : :::::::::
_      AAAATAGGAAACATCACAAAAATCATACCATTCACATCATCATGCCTAGTAATCGGAAGC
          12820     12830     12840     12850     12860     12870

   12860     12870     12880     12890     12900     12910
974467 CTAGCCCTTACCGGAATACCTTTCCTCACAGGATTCTATTCAAAAGATCTCATCATCGAA
       :: ::::: :: :::::::: ::::: ::::: ::::: :::::::: :: :: :: :::
_      CTCGCCCTCACAGGAATACCATTCCTAACAGGGTTCTACTCAAAAGACCTAATTATTGAA
          12880     12890     12900     12910     12920     12930

   12920     12930     12940     12950     12960     12970
974467 GCCATCAACACGTGTAACACCAACGCCTGAGCCCTAATAATCACTTTAATCGCCACATCC
       :: :: :: :: :: ::::::::::::::::::::: :::: ::  :::::::::: :: 
_      GCAATTAATACCTGCAACACCAACGCCTGAGCCCTACTAATTACACTAATCGCCACTTCT
          12940     12950     12960     12970     12980     12990

   12980     12990     13000     13010     13020     13030
974467 ATAACTGCTGTGTACAGCATACGCATCATCTACTTCGTCACCATGACAAAACCACGATAC
       ::::: ::: ::::::::::::: ::::: :::::::: :: :: :::::::: :: :  
_      ATAACAGCTATGTACAGCATACGAATCATTTACTTCGTAACAATAACAAAACCGCGTTTT
          13000     13010     13020     13030     13040     13050

   13040     13050     13060     13070     13080     13090
974467 TCCCCACTAATTACTATTAATGAAAACAACCCAAACCTCATTAATCCAATCAAACGCCTA
        :::: :::::  : ::::: :::::  ::::: ::::::: :: :::::::::::::::
_      CCCCCCCTAATCTCCATTAACGAAAATGACCCAGACCTCATAAACCCAATCAAACGCCTA
          13060     13070     13080     13090     13100     13110

   13100     13110     13120     13130     13140     13150
974467 GCATTAGGAAGCATCCTAGCAGGCTTCCTTATCTCACTAAATATTCCTCCAACTAACATT
       ::::: ::::::::: : ::::: ::  : ::::::   :::::::: ::::: : ::::
_      GCATTCGGAAGCATCTTTGCAGGATTTGTCATCTCATATAATATTCCACCAACCAGCATT
          13120     13130     13140     13150     13160     13170

   13160     13170     13180     13190     13200     13210
974467 CAAATTCTCACAATACCCTGACATTTAAAAATAACAGCCCTACTTATTACAATCCTAGGA
       : : : ::::::::::: :::  ::::::::  ::::::::: ::::: :: :  :::::
_      CCAGTCCTCACAATACCATGATTTTTAAAAACCACAGCCCTAATTATTTCAGTATTAGGA
          13180     13190     13200     13210     13220     13230

   13220     13230     13240     13250     13260     13270
974467 TTTGCCATTGCCCTAGAACTTAACAATTTAACACTAAATCTATCAATAAGCAAACCCACC
       ::    :: :: :::::::: :::::  ::::  :::: ::::::::::  ::: : :  
_      TTCCTAATCGCACTAGAACTAAACAACCTAACCATAAAACTATCAATAAATAAAGCAAAT
          13240     13250     13260     13270     13280     13290

   13280     13290     13300     13310     13320     13330
974467 AAACTATCATCATTCTCAACCTCCCTAGGCTACTACCCACCAATTATACACCGAATTATT
         :   ::::: :::::::: :  :: :: :  : :::: : ::::: ::::: ::::  
_      CCATATTCATCCTTCTCAACTTTACTGGGGTTTTTCCCATCTATTATTCACCGCATTACA
          13300     13310     13320     13330     13340     13350

   13340     13350     13360     13370     13380     13390
974467 CCTCAAAAAACTCTAAATTCTAGCTACAAATTATCCTTAAACCTACTAGACCTAATCTGA
       ::   :::: :::: ::    :::   :::  :::: :::  :: :::::: : ::::: 
_      CCCATAAAATCTCTCAACCTAAGCCTAAAAACATCCCTAACTCTCCTAGACTTGATCTGG
          13360     13370     13380     13390     13400     13410

   13400     13410     13420     13430         13440     13450
974467 CTAGAAAAGACAATCCCAAAATCAACCTCAA----TCACCCAAACCCAACTATCTAAAAT
        ::::::: :: ::::::::::: :::::::    :::: :::::  ::: :  :    :
_      TTAGAAAAAACCATCCCAAAATCCACCTCAACTCTTCACACAAACATAACCACTT----T
          13420     13430     13440     13450     13460

       13460     13470     13480     13490     13500     13510
974467 AATATCCAACCAAAAAGGACTAATCAAACTATATTTCCTATCATTTCTCATCACTATTTC
       :: : :::::::::::::  :::: ::: : :: ::  ::::::: :: :: :  ::   
_      AACAACCAACCAAAAAGGCTTAATTAAATTGTACTTTATATCATTCCTAATTAACATCAT
    13470     13480     13490     13500     13510     13520

       13520     13530     13540     13550     13560     13570
974467 ACTAATTTTTATCTTACACACACTTAATCCCGAGTGATTTCAATAATAATAAAAATCCCC
         ::::: :::::::: :: :: :::::: ::::: :: :: :::::::::::::: :::
_      CTTAATTATTATCTTATACTCAATTAATCTCGAGTAATCTCGATAATAATAAAAATACCC
    13530     13540     13550     13560     13570     13580

       13580     13590     13600     13610     13620     13630
974467 GCAAACAATGACCACCCAGCCACCACTATCATTCAAGTAGCACAACTATAAATAGCCGCA
       :::::::: :: :::::::: :: :: ::::::::::::::::::::::: :: ::::: 
```

```
_       GCAAACAAAGATCACCCAGCTACTACCATCATTCAAGTAGCACAACTATATATTGCCGCT
    13590     13600     13610     13620     13630     13640

       13640     13650     13660     13670     13680     13690
974467 ACCCCAATCCCCCCCTCCAACATAACCCCAACATCATCAATCTCATACATTAATCAATCG
       ::::::::::: :: ::::::::::: ::::::::::::: ::::::::: :: :::::
_      ACCCCAATCCCTCCTTCCAACATAACTCCAACATCATCAACCTCATACATCAACCAATCT
    13650     13660     13670     13680     13690     13700

       13700     13710     13720      13730     13740     13750
974467 CCTAGACTATCAAAATCAACTAACTCCACCTT-ATTATTCAAACTAAACAAGTAGAACAC
       :: : :: ::::: :: :: :: ::::: ::: :: ::   :: ::: ::   : :  :
_      CCCAAACCATCAAGATTAATTA-CTCCAACTTCATCATAATAATTAAGCACACAAATTAA
    13710     13720     13730      13740     13750     13760

        13760     13770     13780     13790     13800     13810
974467 TACCAACTCCATAAAAAGCCCTAAAACAAAAAAACTAAAAATAAACCAATTAGACCCTCA
        :  : ::: ::::  : ::: :: :: :::::::  ::::: :: :: ::::: :: ::
_      AAAAACCTCTATAATCACCCCCAATACTAAAAAACCCAAAATTAATCAGTTAGATCCCCA
     13770     13780     13790     13800     13810     13820

        13820     13830     13840     13850     13860     13870
974467 AGTTTCCGGGTACTCCTCAGTAGCCATAGCAGTTGTATACCCAAATACAACCAACATCCC
       ::: :: :: :: ::::::::::: :::::::: ::::: ::::: ::::::::::::::
_      AGTCTCTGGATATTCCTCAGTAGCTATAGCAGTCGTATATCCAAACACAACCAACATCCC
     13830     13840     13850     13860     13870     13880

        13880     13890     13900     13910     13920     13930
974467 ACCCAAATAAATTAAAAATACTATTAAACCTAAAAACGAACCCCCAAACCCTAAAACTAT
        :: :::::::::::::: :::::::::::::::::::: :: :::::::::::::: ::
_      CCCTAAATAAATTAAAAAAACTATTAAACCTAAAAACGATCCACCAAACCCTAAAACCAT
     13890     13900     13910     13920     13930     13940

        13940     13950     13960     13970     13980     13990
974467 TAAGCACCCAATACATCCACTAACAATCAATCCAAACCCACCATAAATAGGTGAAGGCTT
       ::: :: :::: : : ::::::::::: :: :: :: :: ::::::::::::::::::::
_      TAAACAACCAACAAACCCACTAACAATTAAACCTAAACCTCCATAAATAGGTGAAGGCTT
     13950     13960     13970     13980     13990     14000

        14000     14010     14020     14030     14040     14050
974467 CAACGCCAACCCTAGACAACCAGTCAAAAACAGTAAACTTAAAATAAACATATAATTTGT
        :: :: ::::: :::::::::  :::::: : : ::::::::: ::: ::::::::  :
_      TAATGCTAACCCAAGACAACCAACCAAAAATAATGAACTTAAAACAAAAATATAATTATT
     14010     14020     14030     14040     14050     14060

        14060     14070     14080     14090     14100     14110
974467 CATTATTTCTACACAGCATTTAACTGTGACTAATGACATGAAAAATCATCGTTGTAATTC
       :::::::::::::::::::: ::::: ::: :::::::::::::::::::::::::::::
_      CATTATTTCTACACAGCATTCAACTGCGACCAATGACATGAAAAATCATCGTTGTAATTC
     14070     14080     14090     14100     14110     14120

        14120     14130     14140     14150     14160     14170
974467 AACTATAGAAACATCTAATGACAAACATCCGAAAATCTCACCCCCTATTCAAAATCATCA
       ::::: ::::::: :::::::::::::: :::::: : :::::  :::: ::::: :: :
_      AACTACAGAAACACCTAATGACAAACATACGAAAAACACACCCATTATTTAAAATTATTA
     14130     14140     14150     14160     14170     14180

        14180     14190     14200     14210     14220     14230
974467 ACCACTCCTTTATCGACCTCCCCGCCCCATCTAACATCTCATCATGATGAAACTTCGGTT
       ::::::: :: :: ::::: :: :::::::: ::::: ::::::::::::::::: :: :
_      ACCACTCATTCATTGACCTACCTGCCCCATCCAACATTTCATCATGATGAAACTTTGGGT
     14190     14200     14210     14220     14230     14240

        14240     14250     14260     14270     14280     14290
974467 CTCTACTAGGAGTATGCCTCATAGTACAAATCCTCACAGGCTTATTCCTAGCAATACACT
       : :: :::::::: ::::: ::::: :::::: : :::::  : ::: :::: :::::::
_      CCCTTCTAGGAGTCTGCCTAATAGTCCAAATCATTACAGGTCTTTTCTTAGCCATACACT
     14250     14260     14270     14280     14290     14300

        14300     14310     14320     14330     14340     14350
974467 ACACGTCTGATACCATAACAGCATTCTCATCAGTCACCCACATCTGCCGAGACGTAAACT
       :::: :: ::::: :::::::: :: :::::::: :: ::::: :: ::::::::::: :
_      ACACATCAGATACAATAACAGCCTTTTCATCAGTAACACACATTTGTCGAGACGTAAATT
     14310     14320     14330     14340     14350     14360

        14360     14370     14380     14390     14400     14410
974467 ACGGCTGACTAATCCGATACCTACACGCCAACGGCGCCTCAATATTTTTCATCTGCCTAT
       :::: ::::::::::::::  ::::::: ::::: :::::::::::::: :: ::: :::
_      ACGGGTGACTAATCCGATATATACACGCAAACGGAGCCTCAATATTTTTTATTTGCTTAT
     14370     14380     14390     14400     14410     14420

        14420     14430     14440     14450     14460     14470
974467 TCCTCCATGTGGGACGAGGACTATACTATGGATCCTACACTTTCCTAGAAACCTGAAACA
       :::: ::::: ::::::::  :::: :::::::: :: :: ::  :::::::::::::::
_      TCCTTCATGTCGGACGAGGCTTATATTATGGATCATATACATTTATAGAAACCTGAAACA
     14430     14440     14450     14460     14470     14480

        14480     14490     14500     14510     14520     14530
974467 TTGGGATCATCCTACTATTTGCAGTCATAGCAACTGCATTCATGGGCTATGTACTCCCAT
       ::::  :  : ::::: :: ::::::::::: :: ::::: :: ::::: :: :: ::::
_      TTGGAGTACTTCTACTGTTCGCAGTCATAGCCACAGCATTTATAGGCTACGTCCTTCCAT
     14490     14500     14510     14520     14530     14540
```

```
        14540     14550     14560     14570     14580     14590
974467 GAGGACAAATATCATTCTGAGGAGCTACAGTAATTACAAACCTATTATCAGCTATCCCTT
       :::::::::::::::::::::: :: ::::: :::::::::::  ::::::: ::::: :
_      GAGGACAAATATCATTCTGAGGTGCCACAGTTATTACAAACCTCCTATCAGCCATCCCAT
     14550     14560     14570     14580     14590     14600

        14600     14610     14620     14630     14640     14650
974467 ACATTGGGACTACCCTAGTCGAATGAATCTGAGGAGGCTTCTCAGTAGACAAAGCAACCC
       : ::::: :: ::::::::::::::::: ::::: :::::::::::::::::::: :::
_      ATATTGGAACAACCCTAGTCGAATGAATTTGAGGGGGCTTCTCAGTAGACAAAGCCACCT
     14610     14620     14630     14640     14650     14660

        14660     14670     14680     14690     14700     14710
974467 TAACACGCTTCTTCGCATTCCACTTCATCCTCCCATTCATTATCGCCGCCCTTGCAATTG
       : :: :: :::::::: :::::::::::: : ::::: :::::::: ::::: ::::: :
_      TGACCCGATTCTTCGCTTTCCACTTCATCTTACCATTTATTATCGCGGCCCTAGCAATCG
     14670     14680     14690     14700     14710     14720

        14720     14730     14740     14750     14760     14770
974467 TACATCTTCTTTTCCTCCACGAAACAGGATCAAATAACCCCACAGGATTAAACTCCGACG
       : :: :: :: ::::::::::::::::::::::: ::::: :::::::::::::: :: :
_      TTCACCTCCTCTTCCTCCACGAAACAGGATCAAACAACCCAACAGGATTAAACTCAGATG
     14730     14740     14750     14760     14770     14780

        14780     14790     14800     14810     14820     14830
974467 CAGACAAAATCCCATTCCATCCATATTATACAATTAAAGACCTCCTAGGTGTATTTATAT
       :::: ::::: ::::: :: :: :: :::::::: :::::  :::::::: :  : ::
_      CAGATAAAATTCCATTTCACCCCTACTATACAATCAAAGATATCCTAGGTATCCTAATCA
     14790     14800     14810     14820     14830     14840

        14840     14850     14860     14870     14880     14890
974467 TACTATTATTCCTAATAACCCTAGTACTATTCTTCCCAGACCTACTAGGAGACCCAGACA
       :: : ::: : :: :::::::::::: :::: ::::::::: ::::::::::::::::::
_      TATTCTTAATTCTCATAACCCTAGTATTATTTTTCCCAGACATACTAGGAGACCCAGACA
     14850     14860     14870     14880     14890     14900

        14900     14910     14920     14930     14940     14950
974467 ATTATACACCCGCTAACCCCCTCAACACCCCACCCCACATCAAACCAGAATGATATTTTC
       : :: : ::: ::::: :: :: :::::::::::::: :: ::::: ::::::::::: :
_      ACTACATACCAGCTAATCCACTAAACACCCCACCCCATATTAAACCCGAATGATATTTCC
     14910     14920     14930     14940     14950     14960

        14960     14970     14980     14990     15000     15010
974467 TCTTTGCCTACGCTATTCTACGCTCCATTCCCAACAAACTAGGAGGGGTCGTAGCCCTAA
       : ::::: ::::: ::::::::::: :: ::::: ::::::::::: ::: ::::: :::
_      TATTTGCATACGCCATTCTACGCTCAATCCCCAATAAACTAGGAGGTGTCCTAGCCTTAA
     14970     14980     14990     15000     15010     15020

        15020     15030     15040     15050     15060     15070
974467 TCTTATCAATCCTAATCTTAGCCTTCCTACCATTCCTGCATACCTCAAAACAACGCAGCT
       ::::::: :::::::: :::::: :  :::: ::::: ::::::::::: ::::: :::
_      TCTTATCTATCCTAATTTTAGCCCTAATACCTTTCCTTCATACCTCAAAGCAACGAAGCC
     15030     15040     15050     15060     15070     15080

        15080     15090     15100     15110     15120     15130
974467 TAACATTCCGCCCAATCACCCAAATCCTTTACTGAATCCTAGTAGCCAACCTCCTAGTCT
       ::: ::::::::::::::: :::::  : ::::::::::::::::::::::: ::  :::
_      TAATATTCCGCCCAATCACACAAATTTTGTACTGAATCCTAGTAGCCAACCTACTTATCT
     15090     15100     15110     15120     15130     15140

        15140     15150     15160     15170     15180     15190
974467 TAACATGAATCGGAGGCCAACCAGTAGAACACCCATTTATCATTATTGGTCAACTAGCCT
       :::: ::::: :: :::::::::::::::::::::::::: :: ::::: ::::::::::
_      TAACCTGAATTGGGGGCCAACCAGTAGAACACCCATTTATTATCATTGGCCAACTAGCCT
     15150     15160     15170     15180     15190     15200

        15200     15210     15220     15230     15240     15250
974467 CCATCAGCTATTTTTCAATTATCCTCATTCTCATACCAATCTCTGGAATTGTTGAAGACA
       :::::   :: :: ::::: ::: : ::::: ::::::::::: :::::: : :::::::
_      CCATCTCATACTTCTCAATCATCTTAATTCTTATACCAATCTCAGGAATTATCGAAGACA
     15210     15220     15230     15240     15250     15260

        15260     15270     15280     15290     15300     15310
974467 AAATGTTAAAATGAAATTAATGTCCCGATAGTATAAAAATTACTCTGGTCTTGTAAACCA
       ::::  :::::: : ::  :::::  ::::::::::: :::::::::::::::::::::
_      AAATACTAAAATTATATCCATGTCTTGATAGTATAAACATTACTCTGGTCTTGTAAACCT
     15270     15280     15290     15300     15310     15320

        15320     15330     15340     15350     15360     15370
974467 AAAATGAAGAGTCAGTCTTCTCAGGACAATCAAGAAGAAGGAACTACCTCCCCACCATCA
        :::::::::     :::::::: :::: ::::::::::::: :::: ::::::::: ::
_      GAAATGAAGATCTTCTCTTCTCAAGACA-TCAAGAAGAAGGAGCTAC-TCCCCACCACCA
     15330     15340     15350      15360     15370      15380

        15380     15390     15400     15410     15420     15430
974467 ACACCCAAAGCTGATATTCTACTTAAACTACTTCTTGTCAGTACATAAAATGATATAGGA
        :::::::::::: ::::::: :::::::::::::::  :::::::::: : : :::: :
_      GCACCCAAAGCTGGTATTCTAATTAAACTACTTCTTG--AGTACATAAATTTACATAGTA
       15390     15400     15410     15420       15430     15440

        15440     15450     15460     15470     15480     15490
974467 CATTAAAACATTTATGTATATCGTACATTAAATTATTTTCCCCAAGCATATAAGCATGTA
       ::  :  ::::::::::::::::::::::::: ::::::::::::::::::::::  :::
```

```
_      CAACAGTACATTTATGTATATCGTACATTAAACTATTTTCCCCAAGCATATAAGCTAGTA
         15450     15460     15470     15480     15490     15500

        15500     15510     15520     15530     15540        15550
974467 ATATATATCTAATGATTTAGGACATACATTTAAACTCAACTATAAATTCACA---ACAAC
          :: ::: :::: :: ::: :::: : : :   ::::: ::::::  : :   : : :
_      CATTAAATC-AATGGTTCAGGTCATAAAATAATCATCAAC-ATAAATCAATATATATACC
         15510      15520     15530     15540      15550     15560

           15560       15570     15580      15590     15600
974467 ATGTCTATTCTC--AAATACATTAAGATAATGCTTATTAG-ACATATCTGTGTTATTAGA
       :::  :::: ::  ::: :::::::  ::::: :::: :: :::::::::::::::  ::
_      ATGAATATTATCTTAAACACATTAAACTAATG-TTATAAGGACATATCTGTGTTATCTGA
           15570     15580     15590      15600     15610     15620

    15610     15620     15630     15640     15650     15660
974467 CATGCACCATTAAGTCATAAACCTTTCTCTTCCATATGACTATCCCTGTCCCCAATTGGT
       ::: ::::::  ::::::::::  ::::::::::::::::::::::  :::::: :::::
_      CATACACCATACAGTCATAAACTCTTCTCTTCCATATGACTATCCCCTTCCCCATTTGGT
            15630     15640     15650     15660     15670     15680

    15670     15680     15690     15700     15710     15720
974467 CTCTATTTCTACCATCCTCCGTGAAATCAACAACCCGCCCACTCGTCCCCCTCTTCTCGC
       :: :   :::::::::::::::::::: :::::::::::::::   : :::::::::::::
_      CTATTAATCTACCATCCTCCGTGAAACCAACAACCCGCCCACCAATGCCCCTCTTCTCGC
            15690     15700     15710     15720     15730     15740

    15730     15740     15750     15760     15770     15780
974467 TCCGGGCCCATTCGTCCTGGGGGTGACTATACTGAAACTTTACCAGGCATCTGGTTCTTA
       ::::::::::::   : :::::::  ::: :::::::::::: ::: :::::::::::::
_      TCCGGGCCCATTAAACTTGGGGGTAGCTAAACTGAAACTTTATCAGACATCTGGTTCTTA
            15750     15760     15770     15780     15790     15800

    15790     15800     15810     15820     15830     15840
974467 CTTCAGGGCCATCAATTGGTTCATCGTCCATACGTTCCCCTTAAATAAGACATCTCGATG
       ::::::::::::::: ::  : :::: :::::::::::::::::::::::::::::::::
_      CTTCAGGGCCATCAAATGCGTTATCGCCCATACGTTCCCCTTAAATAAGACATCTCGATG
            15810     15820     15830     15840     15850     15860

    15850     15860     15870     15880     15890     15900
974467 GTAACGGGTCTAATCAGCCCATGATCAACATAACTGTGGTGATATACATTTGGTATTTTT
       ::: :::::::::::::::::::: ::::::::::::::::  :: :::::::::: :::
_      GTATCGGGTCTAATCAGCCCATGACCAACATAACTGTGGTGTCATGCATTTGGTATCTTT
            15870     15880     15890     15900     15910     15920

    15910     15920     15930     15940     15950      15960
974467 TAATTTTCGGATGCCTTCCTCAACATAGCCGTCAAGGCATGAA-GGTCAGCACAAAGTCC
       : ::::: :  : : ::: :::::::::::::::::::::::: :: ::::::: :::: 
_      TTATTTTGGCCTACTTTCATCAACATAGCCGTCAAGGCATGAAAGGACAGCACACAGTCT
            15930     15940     15950     15960     15970     15980

     15970     15980     15990     16000     16010     16020
974467 TGTGGAACCTTTTAGTTAAGGGTCATTTATCCTCATAGACAAAGCTCGAAAGACTATTTT
        :  : ::::    :: :::  :::::  ::: :: :  : :: : :  ::: ::: :: 
_      AGACGCACCTACG-GTGAAGAATCATTAGTCCGCAAAACCCAATCACCTAAGGCTAATT-
            15990      16000     16010     16020     16030

     16030     16040     16050     16060     16070     16080
974467 ATTCATGTTTGTAAGACATAAATATTTATAAATACTGAAAAATCTGTCAACAAACCCCCC
       ::::::: :::: ::::::::::  :  : :::::  ::   :   ::  ::::::::::
_      ATTCATGCTTGTTAGACATAAATGCTACTCAATACCAAATTTTAACTCTCCAAACCCCCC
    16040     16050     16060     16070     16080     16090

     16090     16100     16110     16120     16130      16140
974467 CACCCCCTACACCTGAAACTTCAATGCCAAACCCCAAAAACATTAA-AGCAAGAATTAAA
        :::::::   :::    ::: :::::::::::::::::::: ::: : :  :::  : :
_      -ACCCCCT---CCT----CTT-AATGCCAAACCCCAAAAACACTAAGAACTTGAAAGACA
     16100            16110      16120     16130     16140

      16150     16160     16170     16180     16190     16200
974467 TAAAACAAAAAGCTACTTAATTCTTAAAAGGCTTCTCCATTCTAGTAGACCACAAAATTT
       :: :: :  :: :::   ::  ::  :    ::  :: ::::::::::  : :::::: :
_      TATAATATTAA-CTATCAAACCCT--ATGTCCTGATCAATTCTAGTAGTTCCCAAAATAT
   16150     16160      16170       16180     16190     16200

      16210     16220     16230     16240     16250       16260
974467 TAACTTAAATCTTAGCATTGGTAAAATTTCCCGACACAAAATCTT--TCCTCCTAACTAA
        : :::: :: :::: : : :::::: ::      :::::::: :  :::    : : ::
_      GA-CTTATATTTTAGTACTTGTAAAAATTTT----ACAAAATCATGTTCCGTGAACCAAA
       16210     16220     16230         16240     16250     16260

        16270       16280     16290     16300
974467 ACCCTCTT--TACTTGCCTACCCTCAGAAAATTCCACATACACCAAA
       :: ::  :  ::::    ::: :       : :  ::::  ::  ::
_      ACTCTAATCATACTCTATTACGC-------AATAAACATTAAC--AA
           16270     16280            16290
```

Elapsed time:  0:00:14

**GeneStream align Home Page**

**2. RGC-5 DNA is 100% identical to mouse mitochondrial DNA**



# BLAST

# Basic Local Alignment Search Tool

NCBI/ BLAST/ blastn suite-2sequences/ **Formatting Results - TR2SUYHY111**

**Blast 2 sequences**

**Nucleotide Sequence (380 letters)**

| | | | |
|---|---|---|---|
| **Query ID** | lcl\|59989 | **Subject ID** | gi\|34538597\|ref\|NC_005089.1\| |
| **Description** | None | **Description** | Mus musculus mitochondrion, complete genome |
| **Molecule type** | nucleic acid | **Molecule type** | dna |
| **Query Length** | 380 | **Subject Length** | 16299 |
| | | **Program** | BLASTN 2.2.25+ |

Dot Matrix View

**Plot of lcl|59989 vs gi|34538597|ref|NC_005089.1| [?]**

This dot matrix view shows regions of similarity based upon the BLAST results. The query sequence is represented on the X-axis and the numbers represent the bases/residues of the query. The subject is represented on the Y-axis and again the numbers represent the bases/residues of the subject. Alignments are shown in the plot as lines. Plus strand and protein matches are slanted from the bottom left to the upper right corner, minus strand matches are slanted from the upper left to the lower right. The number of lines shown in the plot is the same as the number of alignments found by BLAST.

## Descriptions

Legend for links to other resources: U UniGene E GEO G Gene S Structure M Map Viewer PubChem BioAssay

| Accession | Description | Max score | Total score | Query coverage | E value | Max ident | Links |
|---|---|---|---|---|---|---|---|
| NC_005089.1 | Mus musculus mitochondrion, complete genome | 553 | 696 | 100% | 2e-160 | 100% | |

## Alignments

```
>ref|NC_005089.1| Mus musculus mitochondrion, complete genome
Length=16299

                                   Sort alignments for this subject sequence by:
                                     E value   Score   Percent identity
                                     Query start position   Subject start position

 Score =  553 bits (299),  Expect = 2e-160
 Identities = 299/299 (100%), Gaps = 0/299 (0%)
```

```
 Strand=Plus/Plus

Query  1      ATCATTAGTCCGCAAAACCCAATCACCTAAGGCTAATTATTCATGCTTGTTAGACATAAA  60
              ||||||||||||||||||||||||||||||||||||||||||||||||||||||||||||
Sbjct  16001  ATCATTAGTCCGCAAAACCCAATCACCTAAGGCTAATTATTCATGCTTGTTAGACATAAA  16060

Query  61     TGCTACTCAATACCAAATTTTAACTCTCCAAAccccccacccccTCCTCTTAATGCCAAA  120
              ||||||||||||||||||||||||||||||||||||||||||||||||||||||||||||
Sbjct  16061  TGCTACTCAATACCAAATTTTAACTCTCCAAACCCCCCACCCCCTCCTCTTAATGCCAAA  16120

Query  121    CCCCAAAAACACTAAGAACTTGAAAGACATATAATATTAACTATCAAACCCTATGTCCTG  180
              ||||||||||||||||||||||||||||||||||||||||||||||||||||||||||||
Sbjct  16121  CCCCAAAAACACTAAGAACTTGAAAGACATATAATATTAACTATCAAACCCTATGTCCTG  16180

Query  181    ATCAATTCTAGTAGTTCCCAAAATATGACTTATATTTTAGTACTTGTAAAAATTTTACAA  240
              ||||||||||||||||||||||||||||||||||||||||||||||||||||||||||||
Sbjct  16181  ATCAATTCTAGTAGTTCCCAAAATATGACTTATATTTTAGTACTTGTAAAAATTTTACAA  16240

Query  241    AATCATGTTCCGTGAACCAAAACTCTAATCATACTCTATTACGCAATAAACATTAACAA  299
              |||||||||||||||||||||||||||||||||||||||||||||||||||||||||||
Sbjct  16241  AATCATGTTCCGTGAACCAAAACTCTAATCATACTCTATTACGCAATAAACATTAACAA  16299


 Score =  143 bits (77),  Expect = 4e-37
 Identities = 80/81 (99%), Gaps = 1/81 (1%)
 Strand=Plus/Plus

Query  300  GTTAATGTAGCTTAATAACAAAGCAAAGCACTGAAAATGCTTAGATGGATAATTGTATCC  359
            ||||||||||||||||||||||||||||||||||||||||||||||||||||||||||||
Sbjct  1    GTTAATGTAGCTTAATAACAAAGCAAAGCACTGAAAATGCTTAGATGGATAATTGTATCC  60

Query  360  CATAAACACAAAGGGTTTGGT  380
            |||||||||||||| ||||||
Sbjct  61   CATAAACACAAAGG-TTTGGT  80
```

**3. Raw data for mean neurites per cell**

| Picture | Cell | Primary Neurites |
|---|---|---|
| 661W 0 1 | 1 | 0 |
| | 2 | 1 |
| | 3 | 0 |
| | 4 | 0 |
| | 5 | 0 |
| | 6 | 0 |
| | 7 | 0 |
| | 8 | 2 |
| | 9 | 0 |
| | 10 | 0 |
| 661W 0 2 | 1 | 0 |
| | 2 | 0 |
| | 3 | 1 |
| | 4 | 0 |
| | 5 | 0 |
| 661W 0 3 | 1 | 0 |
| | 2 | 0 |
| | 3 | 1 |
| | 4 | 0 |
| | 5 | 0 |
| | 6 | 0 |
| | 7 | 0 |
| | 8 | 0 |
| 661W 0 4 | 1 | 1 |
| | 2 | 0 |
| | 3 | 0 |
| | 4 | 0 |
| | 5 | 0 |
| | 6 | 0 |
| | 7 | 0 |
| | 8 | 0 |
| | 9 | 0 |
| 661W 0 5 | 1 | 0 |
| | 2 | 1 |
| | 3 | 0 |
| | 4 | 0 |
| | 5 | 0 |
| 661W 0 6 | 1 | 1 |
| | 2 | 1 |
| | 3 | 0 |
| | 4 | 0 |
| | 5 | 0 |
| | 6 | 0 |
| 661W 100 1 | 1 | 2 |
| | 2 | 0 |
| | 3 | 1 |
| 661W 100 2 | 1 | 4 |
| | 2 | 3 |

| | | |
|---|---|---|
| | 3 | 2 |
| | 4 | 2 |
| | 5 | 2 |
| | 6 | 2 |
| 661W 100 3 | 1 | 1 |
| | 2 | 1 |
| | 3 | 0 |
| 661W 100 4 | 1 | 2 |
| | 2 | 1 |
| | 3 | 2 |
| | 4 | 1 |
| | 5 | 1 |
| | 6 | 2 |
| 661W 100 5 | 1 | 2 |
| | 2 | 2 |
| | 3 | 0 |
| | 4 | 1 |
| | 5 | 1 |
| | 6 | 1 |
| 661W 100 6 | 1 | 3 |
| | 2 | 1 |
| | 3 | 3 |
| | 4 | 2 |
| | 5 | 1 |
| 661W 316 1 | 1 | 2 |
| | 2 | 3 |
| | 3 | 4 |
| | 4 | 2 |
| | 5 | 3 |
| 661W 316 2 | 1 | 3 |
| | 2 | 3 |
| | 3 | 4 |
| | 4 | 3 |
| 661W 316 3 | 1 | 4 |
| | 2 | 2 |
| | 3 | 3 |
| 661W 316 4 | 1 | 2 |
| | 2 | 3 |
| | 3 | 3 |
| | 4 | 4 |
| | 5 | 3 |
| | 6 | 2 |
| | 7 | 2 |
| 661W 316 5 | 1 | 3 |
| | 2 | 4 |
| | 3 | 3 |
| | 4 | 3 |
| | 5 | 4 |
| 661W 316 6 | 1 | 3 |

| | | |
|---|---|---|
| | 2 | 4 |
| | 3 | 3 |
| | 4 | 3 |
| 661W 1000 1 | 1 | 3 |
| | 2 | 3 |
| | 3 | 4 |
| | 4 | 2 |
| 661W 1000 2 | 1 | 1 |
| | 2 | 2 |
| | 3 | 1 |
| | 4 | 0 |
| 661W 1000 3 | 1 | 2 |
| | 2 | 1 |
| | 3 | 1 |
| 661W 1000 4 | 1 | 3 |
| | 2 | 1 |
| | 3 | 1 |
| | 4 | 1 |
| 661W 1000 5 | 1 | 1 |
| | 2 | 1 |
| 661W 1000 6 | 1 | 2 |
| | 2 | 0 |
| | 3 | 2 |
| RGC5 0 1 | 1 | 0 |
| | 2 | 0 |
| | 3 | 1 |
| | 4 | 0 |
| | 5 | 0 |
| | 6 | 0 |
| | 7 | 0 |
| | 8 | 0 |
| | 9 | 0 |
| | 0 | 0 |
| | 10 | 0 |
| | 11 | 0 |
| | 12 | 0 |
| | 13 | 0 |
| | 14 | 0 |
| | 15 | 0 |
| | 16 | 1 |
| | 17 | 1 |
| | 18 | 0 |
| | 19 | 0 |
| | 20 | 0 |
| | 21 | 0 |
| | 22 | 0 |
| | 23 | 1 |
| RGC5 0 2 | 1 | 0 |
| | 2 | 0 |

| | | |
|---|---|---|
| | 3 | 1 |
| | 4 | 1 |
| | 5 | 1 |
| | 6 | 0 |
| | 7 | 0 |
| | 8 | 0 |
| | 9 | 0 |
| | 10 | 0 |
| | 11 | 0 |
| | 12 | 0 |
| | 13 | 1 |
| | 14 | 0 |
| | 15 | 0 |
| | 16 | 0 |
| | 17 | 0 |
| | 18 | 0 |
| | 19 | 0 |
| | 21 | 0 |
| | 22 | 0 |
| RGC5 0 3 | 1 | 1 |
| | 2 | 1 |
| | 3 | 0 |
| | 4 | 1 |
| | 5 | 1 |
| | 6 | 0 |
| | 7 | 0 |
| | 8 | 0 |
| | 9 | 0 |
| | 10 | 1 |
| | 11 | 1 |
| RGC5 0 4 | 1 | 0 |
| | 2 | 0 |
| | 3 | 0 |
| | 4 | 0 |
| | 4 | 0 |
| | 5 | 0 |
| | 6 | 0 |
| | 7 | 0 |
| | 8 | 0 |
| | 9 | 0 |
| | 10 | 0 |
| | 11 | 1 |
| | 12 | 0 |
| | 13 | 0 |
| | 14 | 0 |
| | 15 | 0 |
| | 16 | 1 |
| | 17 | 0 |
| | 18 | 0 |

| | | |
|---|---|---|
| | 19 | 1 |
| | 20 | 0 |
| | 21 | 0 |
| | 22 | 0 |
| | 23 | 1 |
| RGC5 0 5 | 1 | 0 |
| | 2 | 0 |
| | 3 | 0 |
| | 4 | 0 |
| | 5 | 0 |
| | 6 | 0 |
| | 7 | 0 |
| | 8 | 0 |
| | 9 | 0 |
| | 10 | 0 |
| | 11 | 0 |
| | 12 | 0 |
| | 13 | 1 |
| | 14 | 0 |
| | 15 | 0 |
| RGC5 0 6 | 1 | 0 |
| | 2 | 0 |
| | 3 | 0 |
| | 4 | 0 |
| | 5 | 2 |
| | 6 | 0 |
| | 7 | 0 |
| | 8 | 3 |
| | 9 | 0 |
| | 10 | 0 |
| | 11 | 0 |
| | 12 | 0 |
| | 13 | 0 |
| | 14 | 0 |
| | 15 | 0 |
| | 16 | 0 |
| | 17 | 0 |
| | 18 | 0 |
| | 19 | 0 |
| | 20 | 0 |
| | 21 | 0 |
| | 22 | 0 |
| | 23 | 0 |
| | 24 | 0 |
| RGC5 100 1 | 1 | 3 |
| | 2 | 1 |
| | 3 | 0 |
| | 4 | 0 |
| | 5 | 2 |

| | | |
|---|---|---|
| | 6 | 1 |
| RGC5 100 2 | 1 | 2 |
| | 2 | 3 |
| | 3 | 1 |
| | 4 | 2 |
| | 5 | 3 |
| RGC5 100 3 | 1 | 2 |
| | 2 | 1 |
| | 3 | 0 |
| | 4 | 3 |
| | 5 | 2 |
| | 6 | 3 |
| | 7 | 2 |
| RGC5 100 4 | 1 | 2 |
| | 2 | 0 |
| | 3 | 3 |
| | 4 | 2 |
| | 5 | 1 |
| RGC5 100 5 | 1 | 2 |
| | 2 | 4 |
| | 3 | 1 |
| | 4 | 2 |
| | 5 | 2 |
| | 6 | 0 |
| RGC5 100 6 | 1 | 1 |
| | 2 | 3 |
| | 3 | 0 |
| RGC5 316 1 | 1 | 3 |
| | 2 | 4 |
| | 3 | 2 |
| RGC5 316 2 | 1 | 3 |
| | 2 | 1 |
| | 3 | 4 |
| | 4 | 5 |
| | 5 | 4 |
| RGC5 316 3 | 1 | 2 |
| | 2 | 4 |
| | 3 | 2 |
| RGC5 316 4 | 1 | 6 |
| | 2 | 4 |
| RGC5 316 5 | 1 | 3 |
| | 2 | 1 |
| | 3 | 2 |
| | 4 | 3 |
| | 5 | 3 |
| RGC5 316 6 | 1 | 4 |
| | 2 | 3 |
| | 3 | 3 |
| RGC5 1000 1 | 1 | 2 |

| | | |
|---|---|---|
| | 2 | 3 |
| | 3 | 1 |
| | 4 | 3 |
| | 5 | 1 |
| | 6 | 1 |
| RGC5 1000 2 | 1 | 3 |
| | 2 | 2 |
| | 3 | 1 |
| | 4 | 1 |
| | 5 | 1 |
| RGC5 1000 3 | 1 | 0 |
| | 2 | 2 |
| | 3 | 1 |
| | 4 | 2 |
| | 5 | 1 |
| RGC5 1000 4 | 1 | 1 |
| | 2 | 2 |
| | 3 | 2 |
| | 4 | 1 |
| | 5 | 0 |
| RGC5 1000 5 | 1 | 0 |
| | 2 | 1 |
| | 3 | 2 |
| | 4 | 1 |
| | 5 | 1 |
| | 6 | 3 |
| | 7 | 2 |
| RGC5 1000 6 | 1 | 1 |
| | 2 | 1 |
| | 3 | 1 |

| Cell Type | [Staurosporine] | Average Primary Neurites | SEM |
|---|---|---|---|
| 661W | 0 | 0.209302326 | 0.071047685 |
| | 100 nM | 1.586206897 | 0.175586571 |
| | 316 nM | 3.035714286 | 0.130952381 |
| | 1 μM | 1.6 | 0.242535625 |
| | | | |
| RGC-5 | 0 | 0.201680672 | 0.043979239 |
| | 100 nM | 1.6875 | 0.197960875 |
| | 316 nM | 3.142857143 | 0.26979458 |
| | 1 μM | 1.419354839 | 0.152217964 |

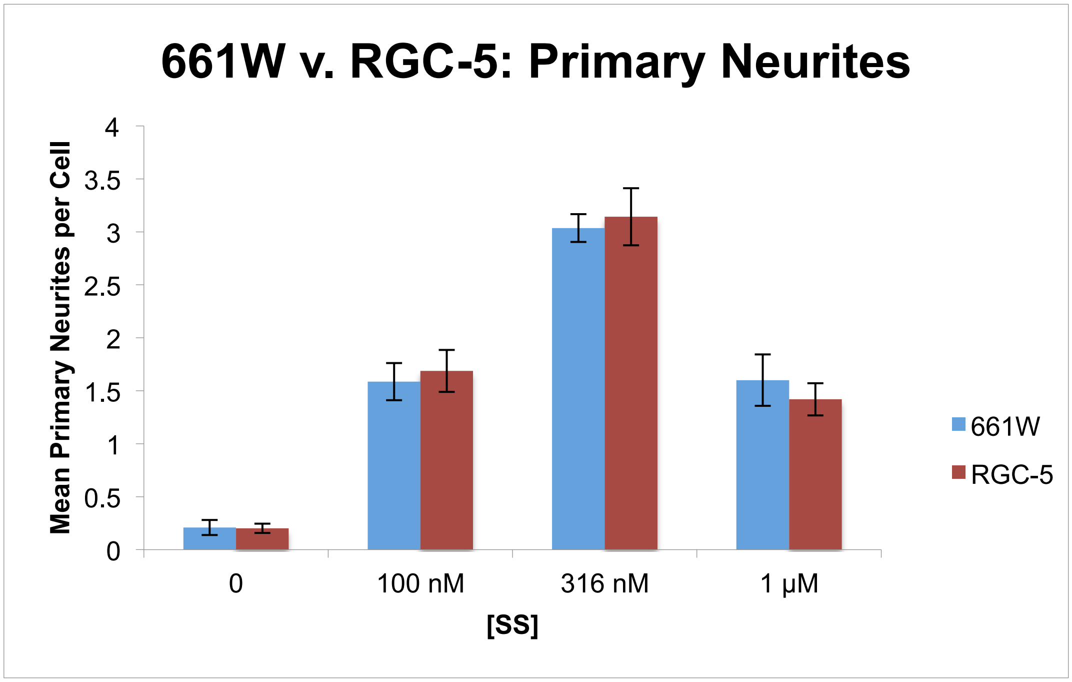

## 4. Neurite length and branch points per cell

| Picture | | Cell | Neurite Type | Length (Pixels) |
|---|---|---|---|---|
| 661W 0SS 1 | N1 | Cluster 02 | Primary | 105.631 |
| 661W 0SS 1 | N2 | Cluster 08 | Primary | 126.427 |
| 661W 0SS 1 | N3 | Cluster 08 | Primary | 82.725 |
| 661W 0SS 2 | N1 | Cluster 03 | Primary | 113.377 |
| 661W 0SS 3 | N1 | Cluster 03 | Primary | 243.588 |
| 661W 0SS 4 | N1 | Cluster 01 | Primary | 120.282 |
| 661W 0SS 5 | N1 | Cluster 02 | Primary | 200.848 |
| 661W 0SS 6 | N1 | Cluster 01 | Primary | 169.85 |
| 661W 100nMSS 1 | N1 | Cluster 01 | Primary | 204.49 |
| 661W 100nMSS 1 | N2 | Cluster 01 | Primary | 279.978 |
| 661W 100nMSS 1 | N3 | Cluster 03 | Primary | 174.95 |
| 661W 100nMSS 2 | N1 | Cluster 01 | Primary | 166.958 |
| 661W 100nMSS 2 | N2 | Cluster 01 | Primary | 108.313 |
| 661W 100nMSS 2 | N3 | Cluster 01 | Primary | 151.602 |
| 661W 100nMSS 2 | N4 | Cluster 01 | Primary | 475.693 |
| 661W 100nMSS 2 | N5 | Cluster 01 | Secondary | 202.589 |
| 661W 100nMSS 2 | N6 | Cluster 02 | Primary | 183.348 |
| 661W 100nMSS 2 | N7 | Cluster 02 | Primary | 131.447 |
| 661W 100nMSS 2 | N8 | Cluster 03 | Primary | 165.054 |
| 661W 100nMSS 3 | N1 | Cluster 01 | Primary | 221.192 |
| 661W 100nMSS 3 | N2 | Cluster 01 | Primary | 168.941 |
| 661W 100nMSS 4 | N1 | Cluster 01 | Primary | 138.627 |
| 661W 100nMSS 4 | N2 | Cluster 01 | Secondary | 29.955 |
| 661W 100nMSS 4 | N3 | Cluster 01 | Primary | 168.669 |
| 661W 100nMSS 4 | N4 | Cluster 02 | Primary | 174.81 |
| 661W 100nMSS 4 | N5 | Cluster 04 | Primary | 113.155 |
| 661W 100nMSS 4 | N6 | Cluster 04 | Primary | 112.817 |
| 661W 100nMSS 4 | N7 | Cluster 05 | Primary | 152.7 |
| 661W 100nMSS 4 | N8 | Cluster 05 | Primary | 78.265 |
| 661W 100nMSS 4 | N9 | Cluster 07 | Primary | 134.634 |
| 661W 100nMSS 4 | N10 | Cluster 08 | Primary | 124.533 |
| 661W 100nMSS 4 | N11 | Cluster 08 | Primary | 92.309 |
| 661W 100nMSS 5 | N1 | Cluster 01 | Primary | 104.727 |
| 661W 100nMSS 5 | N2 | Cluster 01 | Primary | 163.661 |
| 661W 100nMSS 5 | N3 | Cluster 02 | Primary | 98.716 |
| 661W 100nMSS 5 | N4 | Cluster 02 | Secondary | 56.319 |
| 661W 100nMSS 5 | N5 | Cluster 02 | Secondary | 134.393 |
| 661W 100nMSS 5 | N6 | Cluster 02 | Primary | 255.658 |
| 661W 100nMSS 5 | N7 | Cluster 04 | Primary | 145.8 |
| 661W 100nMSS 5 | N8 | Cluster 06 | Primary | 136.255 |
| 661W 100nMSS 5 | N9 | Cluster 06 | Secondary | 177.815 |
| 661W 100nMSS 5 | N10 | Cluster 06 | Tertiary | 152.058 |
| 661W 100nMSS 5 | N11 | Cluster 06 | Tertiary | 40.855 |
| 661W 100nMSS 5 | N12 | Cluster 06 | Secondary | 276.613 |
| 661W 100nMSS 6 | N1 | Cluster 01 | Primary | 256.359 |
| 661W 100nMSS 6 | N2 | Cluster 01 | Primary | 122.703 |
| 661W 100nMSS 6 | N3 | Cluster 01 | Primary | 127.115 |
| 661W 100nMSS 6 | N4 | Cluster 02 | Primary | 85.075 |
| 661W 100nMSS 6 | N5 | Cluster 03 | Primary | 262.959 |
| 661W 316nMSS 1 | N1 | Cluster 01 | Primary | 150.921 |
| 661W 316nMSS 1 | N2 | Cluster 02 | Primary | 140.955 |
| 661W 316nMSS 1 | N3 | Cluster 02 | Primary | 144.107 |
| 661W 316nMSS 1 | N4 | Cluster 03 | Primary | 317.032 |
| 661W 316nMSS 1 | N5 | Cluster 03 | Secondary | 97.507 |
| 661W 316nMSS 1 | N6 | Cluster 03 | Secondary | 153.898 |
| 661W 316nMSS 1 | N7 | Cluster 03 | Primary | 100.465 |
| 661W 316nMSS 1 | N8 | Cluster 03 | Secondary | 64.333 |
| 661W 316nMSS 1 | N9 | Cluster 03 | Secondary | 118.363 |
| 661W 316nMSS 1 | N10 | Cluster 03 | Primary | 152.112 |
| 661W 316nMSS 2 | N1 | Cluster 01 | Primary | 127.938 |
| 661W 316nMSS 2 | N2 | Cluster 01 | Primary | 112.18 |
| 661W 316nMSS 2 | N3 | Cluster 01 | Primary | 147.044 |
| 661W 316nMSS 2 | N4 | Cluster 02 | Primary | 151.856 |
| 661W 316nMSS 2 | N5 | Cluster 02 | Primary | 112.385 |
| 661W 316nMSS 2 | N6 | Cluster 02 | Primary | 136.051 |
| 661W 316nMSS 2 | N7 | Cluster 02 | Primary | 217.799 |
| 661W 316nMSS 2 | N8 | Cluster 03 | Primary | 172.43 |
| 661W 316nMSS 3 | N1 | Cluster 01 | Primary | 41.2 |
| 661W 316nMSS 3 | N2 | Cluster 01 | Secondary | 171.938 |
| 661W 316nMSS 3 | N3 | Cluster 01 | Tertiary | 19.305 |
| 661W 316nMSS 3 | N4 | Cluster 01 | Tertiary | 62.047 |
| 661W 316nMSS 3 | N5 | Cluster 01 | Secondary | 179.792 |
| 661W 316nMSS 3 | N6 | Cluster 01 | Primary | 116.591 |
| 661W 316nMSS 3 | N7 | Cluster 01 | Primary | 114.099 |
| 661W 316nMSS 3 | N8 | Cluster 01 | Secondary | 67.813 |
| 661W 316nMSS 3 | N9 | Cluster 01 | Secondary | 87.665 |
| 661W 316nMSS 3 | N10 | Cluster 01 | Secondary | 55.085 |

| | | | | |
|---|---|---|---|---|
| 661W 316nMSS 3 | N11 | Cluster 01 | Primary | 101.948 |
| 661W 316nMSS 3 | N12 | Cluster 02 | Primary | 142.059 |
| 661W 316nMSS 3 | N13 | Cluster 02 | Primary | 99.149 |
| 661W 316nMSS 3 | N14 | Cluster 03 | Primary | 107.969 |
| 661W 316nMSS 3 | N15 | Cluster 03 | Primary | 121.234 |
| 661W 316nMSS 3 | N16 | Cluster 03 | Secondary | 149.062 |
| 661W 316nMSS 3 | N17 | Cluster 03 | Secondary | 71.091 |
| 661W 316nMSS 4 | N1 | Cluster 02 | Primary | 241.348 |
| 661W 316nMSS 4 | N2 | Cluster 03 | Primary | 102.277 |
| 661W 316nMSS 4 | N3 | Cluster 03 | Primary | 248.396 |
| 661W 316nMSS 4 | N4 | Cluster 03 | Primary | 84.041 |
| 661W 316nMSS 4 | N5 | Cluster 04 | Primary | 147.848 |
| 661W 316nMSS 4 | N6 | Cluster 04 | Secondary | 22.931 |
| 661W 316nMSS 4 | N7 | Cluster 04 | Secondary | 67.56 |
| 661W 316nMSS 4 | N8 | Cluster 04 | Primary | 33.091 |
| 661W 316nMSS 4 | N9 | Cluster 04 | Secondary | 23.17 |
| 661W 316nMSS 4 | N10 | Cluster 04 | Secondary | 45.255 |
| 661W 316nMSS 4 | N11 | Cluster 05 | Primary | 94.71 |
| 661W 316nMSS 4 | N12 | Cluster 05 | Primary | 87.151 |
| 661W 316nMSS 4 | N13 | Cluster 06 | Primary | 40.98 |
| 661W 316nMSS 4 | N14 | Cluster 06 | Secondary | 108.112 |
| 661W 316nMSS 4 | N15 | Cluster 06 | Secondary | 68.865 |
| 661W 316nMSS 4 | N16 | Cluster 06 | Primary | 250.438 |
| 661W 316nMSS 4 | N17 | Cluster 07 | Primary | 236.703 |
| 661W 316nMSS 4 | N18 | Cluster 07 | Secondary | 78.815 |
| 661W 316nMSS 4 | N19 | Cluster 07 | Secondary | 82.868 |
| 661W 316nMSS 4 | N20 | Cluster 07 | Primary | 98.004 |
| 661W 316nMSS 4 | N21 | Cluster 07 | Secondary | 91.423 |
| 661W 316nMSS 4 | N22 | Cluster 07 | Secondary | 183.388 |
| 661W 316nMSS 4 | N23 | Cluster 07 | Tertiary | 17.493 |
| 661W 316nMSS 4 | N24 | Cluster 07 | Tertiary | 105.992 |
| 661W 316nMSS 4 | N25 | Cluster 07 | Primary | 117.608 |
| 661W 316nMSS 4 | N26 | Cluster 08 | Primary | 98.032 |
| 661W 316nMSS 4 | N27 | Cluster 08 | Primary | 97.682 |
| 661W 316nMSS 4 | N28 | Cluster 09 | Primary | 148.988 |
| 661W 316nMSS 4 | N29 | Cluster 09 | Primary | 145.128 |
| 661W 316nMSS 4 | N30 | Cluster 09 | Secondary | 39.787 |
| 661W 316nMSS 4 | N31 | Cluster 09 | Secondary | 44.253 |
| 661W 316nMSS 5 | N1 | Cluster 01 | Primary | 137.326 |
| 661W 316nMSS 5 | N2 | Cluster 01 | Secondary | 294.668 |
| 661W 316nMSS 5 | N3 | Cluster 01 | Tertiary | 78.457 |
| 661W 316nMSS 5 | N4 | Cluster 01 | Tertiary | 158.037 |
| 661W 316nMSS 5 | N5 | Cluster 01 | Secondary | 110.298 |
| 661W 316nMSS 5 | N6 | Cluster 01 | Primary | 27.579 |
| 661W 316nMSS 5 | N7 | Cluster 01 | Secondary | 56.423 |
| 661W 316nMSS 5 | N8 | Cluster 01 | Secondary | 39.622 |
| 661W 316nMSS 5 | N9 | Cluster 01 | Tertiary | 31.316 |
| 661W 316nMSS 5 | N10 | Cluster 01 | Tertiary | 29.492 |
| 661W 316nMSS 5 | N11 | Cluster 01 | Primary | 16.601 |
| 661W 316nMSS 5 | N12 | Cluster 01 | Secondary | 54.233 |
| 661W 316nMSS 5 | N13 | Cluster 01 | Secondary | 51.004 |
| 661W 316nMSS 5 | N14 | Cluster 01 | Tertiary | 41.276 |
| 661W 316nMSS 5 | N15 | Cluster 01 | Tertiary | 47.415 |
| 661W 316nMSS 5 | N16 | Cluster 02 | Primary | 75.432 |
| 661W 316nMSS 5 | N17 | Cluster 02 | Secondary | 139.578 |
| 661W 316nMSS 5 | N18 | Cluster 02 | Secondary | 36.308 |
| 661W 316nMSS 5 | N19 | Cluster 02 | Tertiary | 37.284 |
| 661W 316nMSS 5 | N20 | Cluster 02 | Tertiary | 108.613 |
| 661W 316nMSS 5 | N21 | Cluster 02 | Primary | 134.255 |
| 661W 316nMSS 5 | N22 | Cluster 02 | Secondary | 34.325 |
| 661W 316nMSS 5 | N23 | Cluster 02 | Tertiary | 31.271 |
| 661W 316nMSS 5 | N24 | Cluster 02 | Tertiary | 18.459 |
| 661W 316nMSS 5 | N25 | Cluster 02 | Secondary | 58.957 |
| 661W 316nMSS 5 | N26 | Cluster 02 | Tertiary | 22.001 |
| 661W 316nMSS 5 | N27 | Cluster 02 | Tertiary | 69.026 |
| 661W 316nMSS 5 | N28 | Cluster 02 | Primary | 68.872 |
| 661W 316nMSS 5 | N29 | Cluster 02 | Secondary | 38.858 |
| 661W 316nMSS 5 | N30 | Cluster 02 | Secondary | 27.733 |
| 661W 316nMSS 5 | N31 | Cluster 02 | Tertiary | 29.127 |
| 661W 316nMSS 5 | N32 | Cluster 02 | Tertiary | 39.278 |
| 661W 316nMSS 5 | N33 | Cluster 02 | Type 06 | 33.139 |
| 661W 316nMSS 5 | N35 | Cluster 02 | Type 06 | 47.453 |
| 661W 316nMSS 5 | N36 | Cluster 02 | Primary | 61.71 |
| 661W 316nMSS 5 | N37 | Cluster 02 | Secondary | 53.927 |
| 661W 316nMSS 5 | N38 | Cluster 02 | Secondary | 74.296 |
| 661W 316nMSS 5 | N39 | Cluster 02 | Secondary | 74.912 |
| 661W 316nMSS 5 | N40 | Cluster 02 | Tertiary | 152.267 |
| 661W 316nMSS 5 | N41 | Cluster 02 | Type 06 | 105.077 |

| | | | | |
|---|---|---|---|---|
| 661W 316nMSS 5 | N42 | Cluster 02 | Type 06 | 209.883 |
| 661W 316nMSS 5 | N43 | Cluster 01 | Secondary | 86.997 |
| 661W 316nMSS 6 | N1 | Cluster 01 | Primary | 61.266 |
| 661W 316nMSS 6 | N2 | Cluster 01 | Secondary | 128.6 |
| 661W 316nMSS 6 | N3 | Cluster 01 | Secondary | 115.297 |
| 661W 316nMSS 6 | N4 | Cluster 01 | Primary | 126.803 |
| 661W 316nMSS 6 | N5 | Cluster 01 | Primary | 82.451 |
| 661W 316nMSS 6 | N6 | Cluster 02 | Primary | 183.923 |
| 661W 316nMSS 6 | N7 | Cluster 02 | Primary | 213.466 |
| 661W 316nMSS 6 | N8 | Cluster 02 | Secondary | 58.945 |
| 661W 316nMSS 6 | N9 | Cluster 02 | Tertiary | 62.758 |
| 661W 316nMSS 6 | N10 | Cluster 02 | Tertiary | 27.456 |
| 661W 316nMSS 6 | N11 | Cluster 02 | Secondary | 51.018 |
| 661W 316nMSS 6 | N12 | Cluster 02 | Primary | 170.53 |
| 661W 316nMSS 6 | N13 | Cluster 03 | Primary | 107.63 |
| 661W 316nMSS 6 | N14 | Cluster 03 | Primary | 34.72 |
| 661W 316nMSS 6 | N15 | Cluster 03 | Secondary | 72.091 |
| 661W 316nMSS 6 | N16 | Cluster 03 | Secondary | 31.709 |
| 661W 316nMSS 6 | N17 | Cluster 03 | Tertiary | 35.539 |
| 661W 316nMSS 6 | N18 | Cluster 03 | Tertiary | 28.601 |
| 661W 316nMSS 6 | N19 | Cluster 03 | Type 06 | 26.942 |
| 661W 316nMSS 6 | N20 | Cluster 03 | Type 06 | 18.583 |
| 661W 316nMSS 6 | N21 | Cluster 03 | Primary | 110.698 |
| 661W 316nMSS 6 | N22 | Cluster 03 | Secondary | 42.32 |
| 661W 316nMSS 6 | N23 | Cluster 03 | Secondary | 50.612 |
| 661W 316nMSS 6 | N25 | Cluster 03 | Primary | 81.463 |
| 661W 316nMSS 6 | N27 | Cluster 04 | Primary | 73.668 |
| 661W 316nMSS 6 | N28 | Cluster 04 | Primary | 80.878 |
| 661W 316nMSS 6 | N29 | Cluster 04 | Secondary | 29.727 |
| 661W 316nMSS 6 | N30 | Cluster 04 | Secondary | 95.398 |
| 661W 316nMSS 6 | N31 | Cluster 04 | Primary | 117.025 |
| 661W 316nMSS 6 | N32 | Cluster 05 | Primary | 48.527 |
| 661W 316nMSS 6 | N33 | Cluster 05 | Secondary | 54.202 |
| 661W 316nMSS 6 | N34 | Cluster 05 | Secondary | 50.437 |
| 661W 316nMSS 6 | N35 | Cluster 05 | Primary | 69.915 |
| 661W 316nMSS 6 | N36 | Cluster 05 | Secondary | 61.544 |
| 661W 316nMSS 6 | N37 | Cluster 05 | Secondary | 60.773 |
| 661W 316nMSS 6 | N38 | Cluster 05 | Primary | 50.412 |
| 661W 316nMSS 6 | N39 | Cluster 05 | Secondary | 36.997 |
| 661W 316nMSS 6 | N40 | Cluster 05 | Secondary | 45.165 |
| 661W 1uMSS 1 | N1 | Cluster 01 | Primary | 21.109 |
| 661W 1uMSS 1 | N2 | Cluster 01 | Secondary | 20.047 |
| 661W 1uMSS 1 | N3 | Cluster 01 | Secondary | 52.344 |
| 661W 1uMSS 1 | N4 | Cluster 01 | Tertiary | 62.197 |
| 661W 1uMSS 1 | N5 | Cluster 01 | Tertiary | 33.988 |
| 661W 1uMSS 1 | N6 | Cluster 01 | Primary | 38.804 |
| 661W 1uMSS 1 | N7 | Cluster 01 | Secondary | 129.427 |
| 661W 1uMSS 1 | N10 | Cluster 01 | Secondary | 199.983 |
| 661W 1uMSS 1 | N11 | Cluster 01 | Primary | 279.357 |
| 661W 1uMSS 1 | N13 | Cluster 02 | Primary | 59.85 |
| 661W 1uMSS 1 | N14 | Cluster 02 | Secondary | 20.884 |
| 661W 1uMSS 1 | N15 | Cluster 02 | Secondary | 65.429 |
| 661W 1uMSS 1 | N16 | Cluster 02 | Primary | 58.252 |
| 661W 1uMSS 1 | N17 | Cluster 02 | Primary | 25.441 |
| 661W 1uMSS 1 | N18 | Cluster 02 | Secondary | 29.965 |
| 661W 1uMSS 1 | N19 | Cluster 02 | Tertiary | 23.672 |
| 661W 1uMSS 1 | N20 | Cluster 02 | Tertiary | 26.87 |
| 661W 1uMSS 1 | N21 | Cluster 02 | Secondary | 63.786 |
| 661W 1uMSS 1 | N22 | Cluster 02 | Tertiary | 63.234 |
| 661W 1uMSS 1 | N23 | Cluster 02 | Type 06 | 49.084 |
| 661W 1uMSS 1 | N24 | Cluster 02 | Type 06 | 47.687 |
| 661W 1uMSS 1 | N25 | Cluster 02 | Tertiary | 74.228 |
| 661W 1uMSS 1 | N26 | Cluster 02 | Primary | 83.217 |
| 661W 1uMSS 1 | N27 | Cluster 02 | Secondary | 133.6 |
| 661W 1uMSS 1 | N28 | Cluster 02 | Secondary | 22.432 |
| 661W 1uMSS 1 | N29 | Cluster 02 | Tertiary | 31.926 |
| 661W 1uMSS 1 | N30 | Cluster 02 | Tertiary | 27.785 |
| 661W 1uMSS 1 | N31 | Cluster 02 | Type 06 | 34.54 |
| 661W 1uMSS 1 | N32 | Cluster 02 | Type 06 | 32.792 |
| 661W 1uMSS 1 | N33 | Cluster 02 | Type 06 | 31.029 |
| 661W 1uMSS 1 | N34 | Cluster 02 | Type 06 | 43.591 |
| 661W 1uMSS 1 | N35 | Cluster 02 | Type 06 | 29.682 |
| 661W 1uMSS 1 | N36 | Cluster 02 | Type 06 | 25.958 |
| 661W 1uMSS 1 | N38 | Cluster 02 | Primary | 85.008 |
| 661W 1uMSS 1 | N39 | Cluster 03 | Primary | 71.851 |
| 661W 1uMSS 1 | N40 | Cluster 03 | Primary | 83.947 |
| 661W 1uMSS 1 | N41 | Cluster 03 | Secondary | 71.327 |
| 661W 1uMSS 1 | N42 | Cluster 03 | Secondary | 44.315 |

| | | | | |
|---|---|---|---|---|
| 661W 1uMSS 1 | N43 | Cluster 03 | Primary | 187.363 |
| 661W 1uMSS 1 | N44 | Cluster 03 | Primary | 75.833 |
| 661W 1uMSS 1 | N45 | Cluster 04 | Primary | 62.26 |
| 661W 1uMSS 1 | N46 | Cluster 04 | Primary | 86.084 |
| 661W 1uMSS 1 | N47 | Cluster 04 | Secondary | 86.504 |
| 661W 1uMSS 1 | N48 | Cluster 04 | Secondary | 109.63 |
| 661W 1uMSS 2 | N1 | Cluster 01 | Primary | 129.018 |
| 661W 1uMSS 2 | N2 | Cluster 02 | Primary | 64.672 |
| 661W 1uMSS 2 | N3 | Cluster 02 | Secondary | 57.805 |
| 661W 1uMSS 2 | N4 | Cluster 02 | Secondary | 57.856 |
| 661W 1uMSS 2 | N5 | Cluster 02 | Secondary | 37.475 |
| 661W 1uMSS 2 | N6 | Cluster 02 | Primary | 148.007 |
| 661W 1uMSS 2 | N7 | Cluster 02 | Primary | 33.261 |
| 661W 1uMSS 2 | N9 | Cluster 02 | Secondary | 24.305 |
| 661W 1uMSS 2 | N10 | Cluster 02 | Secondary | 22.718 |
| 661W 1uMSS 2 | N11 | Cluster 02 | Tertiary | 19.824 |
| 661W 1uMSS 2 | N12 | Cluster 02 | Tertiary | 9.954 |
| 661W 1uMSS 2 | N15 | Cluster 03 | Primary | 109.172 |
| 661W 1uMSS 2 | N16 | Cluster 03 | Secondary | 27.628 |
| 661W 1uMSS 2 | N17 | Cluster 03 | Secondary | 38.172 |
| 661W 1uMSS 2 | N18 | Cluster 03 | Primary | 157.996 |
| 661W 1uMSS 2 | N19 | Cluster 03 | Secondary | 53.995 |
| 661W 1uMSS 2 | N20 | Cluster 03 | Secondary | 111.532 |
| 661W 1uMSS 2 | N21 | Cluster 03 | Primary | 110.011 |
| 661W 1uMSS 3 | N1 | Cluster 01 | Primary | 99.12 |
| 661W 1uMSS 3 | N2 | Cluster 01 | Primary | 96.774 |
| 661W 1uMSS 4 | N1 | Cluster 01 | Primary | 29.267 |
| 661W 1uMSS 4 | N2 | Cluster 01 | Secondary | 66.134 |
| 661W 1uMSS 4 | N3 | Cluster 01 | Secondary | 49.01 |
| 661W 1uMSS 4 | N4 | Cluster 01 | Primary | 44.483 |
| 661W 1uMSS 4 | N5 | Cluster 01 | Secondary | 25.719 |
| 661W 1uMSS 4 | N6 | Cluster 01 | Secondary | 30.226 |
| 661W 1uMSS 4 | N7 | Cluster 01 | Secondary | 62.518 |
| 661W 1uMSS 4 | N8 | Cluster 01 | Primary | 68.126 |
| 661W 1uMSS 4 | N9 | Cluster 01 | Primary | 44.504 |
| 661W 1uMSS 4 | N10 | Cluster 01 | Secondary | 31.937 |
| 661W 1uMSS 4 | N11 | Cluster 01 | Secondary | 32.916 |
| 661W 1uMSS 4 | N12 | Cluster 01 | Tertiary | 26.621 |
| 661W 1uMSS 4 | N13 | Cluster 01 | Tertiary | 104.098 |
| 661W 1uMSS 4 | N14 | Cluster 02 | Primary | 109.993 |
| 661W 1uMSS 4 | N15 | Cluster 03 | Primary | 98.265 |
| 661W 1uMSS 4 | N16 | Cluster 04 | Primary | 85.539 |
| 661W 1uMSS 5 | N1 | Cluster 01 | Primary | 85.194 |
| 661W 1uMSS 5 | N4 | Cluster 02 | Primary | 39.479 |
| 661W 1uMSS 5 | N5 | Cluster 02 | Secondary | 90.598 |
| 661W 1uMSS 5 | N6 | Cluster 02 | Secondary | 174.318 |
| 661W 1uMSS 5 | N7 | Cluster 02 | Tertiary | 36.854 |
| 661W 1uMSS 5 | N8 | Cluster 02 | Tertiary | 63.965 |
| 661W 1uMSS 5 | N9 | Cluster 02 | Primary | 31.986 |
| 661W 1uMSS 5 | N10 | Cluster 02 | Secondary | 93.123 |
| 661W 1uMSS 5 | N11 | Cluster 02 | Secondary | 65.667 |
| 661W 1uMSS 5 | N12 | Cluster 03 | Primary | 111.961 |
| 661W 1uMSS 6 | N1 | Cluster 01 | Primary | 45.586 |
| 661W 1uMSS 6 | N2 | Cluster 01 | Secondary | 63.183 |
| 661W 1uMSS 6 | N3 | Cluster 01 | Secondary | 131.489 |
| 661W 1uMSS 6 | N4 | Cluster 01 | Secondary | 131.894 |
| 661W 1uMSS 6 | N5 | Cluster 01 | Primary | 66.657 |
| 661W 1uMSS 6 | N6 | Cluster 01 | Primary | 31.843 |
| 661W 1uMSS 6 | N7 | Cluster 01 | Secondary | 27.859 |
| 661W 1uMSS 6 | N8 | Cluster 01 | Secondary | 59.924 |
| 661W 1uMSS 6 | N9 | Cluster 01 | Primary | 82.613 |
| 661W 1uMSS 6 | N10 | Cluster 01 | Primary | 60.706 |
| | | | | |
| RGC5 0SS 1 | N1 | Cluster 03 | Primary | 77.166 |
| RGC5 0SS 1 | N2 | Cluster 06 | Primary | 146.597 |
| RGC5 0SS 1 | N3 | Cluster 07 | Primary | 103.578 |
| RGC5 0SS 1 | N4 | Cluster 10 | Primary | 123.472 |
| RGC5 0SS 2 | N1 | Cluster 03 | Primary | 101.245 |
| RGC5 0SS 2 | N2 | Cluster 04 | Primary | 87.76 |
| RGC5 0SS 2 | N3 | Cluster 05 | Primary | 133.501 |
| RGC5 0SS 2 | N4 | Cluster 10 | Primary | 67.768 |
| RGC5 0SS 3 | N1 | Cluster 01 | Primary | 116.628 |
| RGC5 0SS 3 | N2 | Cluster 02 | Primary | 99.412 |
| RGC5 0SS 3 | N3 | Cluster 04 | Primary | 97.032 |
| RGC5 0SS 3 | N4 | Cluster 05 | Primary | 98.021 |
| RGC5 0SS 3 | N5 | Cluster 10 | Primary | 162.64 |
| RGC5 0SS 3 | N6 | Cluster 09 | Primary | 131.166 |
| RGC5 0SS 4 | N1 | Cluster 01 | Primary | 162.836 |

| | | | | |
|---|---|---|---|---|
| RGC5 0SS 4 | N2 | Cluster 06 | Primary | 180.106 |
| RGC5 0SS 4 | N3 | Cluster 09 | Primary | 124.636 |
| RGC5 0SS 4 | N4 | Cluster 03 | Primary | 152.201 |
| RGC5 0SS 5 | N1 | Cluster 03 | Primary | 129.661 |
| RGC5 0SS 6 | N1 | Cluster 05 | Primary | 143.398 |
| RGC5 0SS 6 | N2 | Cluster 05 | Primary | 129.249 |
| RGC5 0SS 6 | N3 | Cluster 08 | Primary | 132.502 |
| RGC5 0SS 6 | N4 | Cluster 08 | Primary | 158.996 |
| RGC5 0SS 6 | N5 | Cluster 08 | Primary | 131.727 |
| RGC5 100nMSS 1 | N1 | Cluster 01 | Primary | 81.276 |
| RGC5 100nMSS 1 | N2 | Cluster 01 | Primary | 45.274 |
| RGC5 100nMSS 1 | N3 | Cluster 01 | Secondary | 58.116 |
| RGC5 100nMSS 1 | N4 | Cluster 01 | Tertiary | 57.411 |
| RGC5 100nMSS 1 | N5 | Cluster 01 | Tertiary | 55.063 |
| RGC5 100nMSS 1 | N6 | Cluster 01 | Secondary | 45.166 |
| RGC5 100nMSS 1 | N7 | Cluster 01 | Primary | 99.432 |
| RGC5 100nMSS 1 | N8 | Cluster 04 | Primary | 192.49 |
| RGC5 100nMSS 2 | N1 | Cluster 01 | Primary | 56.103 |
| RGC5 100nMSS 2 | N2 | Cluster 01 | Secondary | 81.139 |
| RGC5 100nMSS 2 | N3 | Cluster 01 | Secondary | 112.632 |
| RGC5 100nMSS 2 | N4 | Cluster 01 | Tertiary | 90.829 |
| RGC5 100nMSS 2 | N5 | Cluster 01 | Tertiary | 109.578 |
| RGC5 100nMSS 2 | N6 | Cluster 01 | Primary | 296.894 |
| RGC5 100nMSS 2 | N7 | Cluster 02 | Primary | 75.031 |
| RGC5 100nMSS 2 | N8 | Cluster 02 | Secondary | 119.436 |
| RGC5 100nMSS 2 | N9 | Cluster 02 | Secondary | 237.454 |
| RGC5 100nMSS 2 | N10 | Cluster 02 | Primary | 163.856 |
| RGC5 100nMSS 2 | N11 | Cluster 03 | Primary | 196.146 |
| RGC5 100nMSS 2 | N12 | Cluster 03 | Primary | 173.35 |
| RGC5 100nMSS 2 | N13 | Cluster 04 | Primary | 207.574 |
| RGC5 100nMSS 2 | N14 | Cluster 04 | Primary | 234.936 |
| RGC5 100nMSS 3 | N1 | Cluster 01 | Primary | 138.281 |
| RGC5 100nMSS 3 | N2 | Cluster 02 | Primary | 67.884 |
| RGC5 100nMSS 3 | N3 | Cluster 02 | Secondary | 84.662 |
| RGC5 100nMSS 3 | N4 | Cluster 02 | Secondary | 65.573 |
| RGC5 100nMSS 3 | N5 | Cluster 02 | Primary | 245.029 |
| RGC5 100nMSS 3 | N6 | Cluster 02 | Primary | 119.802 |
| RGC5 100nMSS 3 | N7 | Cluster 02 | Secondary | 105.821 |
| RGC5 100nMSS 3 | N8 | Cluster 02 | Secondary | 115.624 |
| RGC5 100nMSS 3 | N9 | Cluster 03 | Primary | 53.651 |
| RGC5 100nMSS 3 | N10 | Cluster 03 | Secondary | 215.459 |
| RGC5 100nMSS 3 | N11 | Cluster 03 | Secondary | 97.951 |
| RGC5 100nMSS 3 | N12 | Cluster 04 | Primary | 130.528 |
| RGC5 100nMSS 3 | N13 | Cluster 04 | Primary | 85.757 |
| RGC5 100nMSS 3 | N14 | Cluster 04 | Secondary | 69.683 |
| RGC5 100nMSS 3 | N15 | Cluster 04 | Secondary | 113.954 |
| RGC5 100nMSS 3 | N16 | Cluster 04 | Primary | 87.768 |
| RGC5 100nMSS 3 | N17 | Cluster 05 | Primary | 107.67 |
| RGC5 100nMSS 3 | N18 | Cluster 06 | Primary | 449.547 |
| RGC5 100nMSS 3 | N19 | Cluster 07 | Primary | 138.043 |
| RGC5 100nMSS 3 | N20 | Cluster 07 | Primary | 236.373 |
| RGC5 100nMSS 4 | N2 | Cluster 01 | Primary | 95.062 |
| RGC5 100nMSS 4 | N3 | Cluster 02 | Primary | 122.391 |
| RGC5 100nMSS 4 | N4 | Cluster 03 | Primary | 102.716 |
| RGC5 100nMSS 4 | N5 | Cluster 03 | Primary | 42.505 |
| RGC5 100nMSS 4 | N6 | Cluster 03 | Secondary | 111.686 |
| RGC5 100nMSS 4 | N7 | Cluster 03 | Secondary | 85.979 |
| RGC5 100nMSS 4 | N8 | Cluster 03 | Primary | 108.643 |
| RGC5 100nMSS 4 | N9 | Cluster 04 | Primary | 90.587 |
| RGC5 100nMSS 4 | N10 | Cluster 04 | Primary | 107.168 |
| RGC5 100nMSS 4 | N11 | Cluster 05 | Primary | 127.603 |
| RGC5 100nMSS 4 | N12 | Cluster 05 | Secondary | 60.5 |
| RGC5 100nMSS 4 | N13 | Cluster 05 | Secondary | 192.764 |
| RGC5 100nMSS 5 | N1 | Cluster 01 | Primary | 84.657 |
| RGC5 100nMSS 5 | N2 | Cluster 02 | Primary | 105.249 |
| RGC5 100nMSS 5 | N3 | Cluster 02 | Secondary | 45.042 |
| RGC5 100nMSS 5 | N4 | Cluster 02 | Secondary | 61.255 |
| RGC5 100nMSS 5 | N5 | Cluster 02 | Primary | 140.096 |
| RGC5 100nMSS 5 | N6 | Cluster 03 | Primary | 143.664 |
| RGC5 100nMSS 5 | N7 | Cluster 04 | Primary | 107.794 |
| RGC5 100nMSS 5 | N8 | Cluster 05 | Primary | 34.834 |
| RGC5 100nMSS 5 | N9 | Cluster 05 | Secondary | 27.579 |
| RGC5 100nMSS 5 | N10 | Cluster 05 | Tertiary | 48.648 |
| RGC5 100nMSS 5 | N11 | Default | Tertiary | 104.352 |
| RGC5 100nMSS 5 | N12 | Cluster 05 | Secondary | 91.652 |
| RGC5 100nMSS 6 | N1 | Cluster 01 | Primary | 124.609 |
| RGC5 100nMSS 6 | N2 | Cluster 02 | Primary | 144.708 |
| RGC5 316nMSS 1 | N1 | Cluster 01 | Primary | 59.516 |

| | | | | |
|---|---|---|---|---|
| RGC5 316nMSS 1 | N2 | Cluster 01 | Secondary | 16.636 |
| RGC5 316nMSS 1 | N3 | Cluster 01 | Tertiary | 30.558 |
| RGC5 316nMSS 1 | N4 | Cluster 01 | Tertiary | 30.091 |
| RGC5 316nMSS 1 | N5 | Cluster 01 | Secondary | 88.704 |
| RGC5 316nMSS 1 | N6 | Cluster 01 | Primary | 33.329 |
| RGC5 316nMSS 1 | N7 | Cluster 01 | Secondary | 33.558 |
| RGC5 316nMSS 1 | N8 | Cluster 01 | Secondary | 77.751 |
| RGC5 316nMSS 1 | N9 | Cluster 01 | Primary | 35.099 |
| RGC5 316nMSS 1 | N10 | Cluster 01 | Secondary | 52.072 |
| RGC5 316nMSS 1 | N11 | Cluster 01 | Secondary | 45.166 |
| RGC5 316nMSS 1 | N12 | Cluster 01 | Secondary | 43.979 |
| RGC5 316nMSS 1 | N13 | Cluster 02 | Primary | 92.3 |
| RGC5 316nMSS 1 | N14 | Cluster 02 | Secondary | 59.855 |
| RGC5 316nMSS 1 | N15 | Cluster 02 | Secondary | 31.067 |
| RGC5 316nMSS 1 | N16 | Cluster 02 | Primary | 94.593 |
| RGC5 316nMSS 2 | N1 | Cluster 01 | Primary | 48.693 |
| RGC5 316nMSS 2 | N2 | Cluster 01 | Secondary | 79.433 |
| RGC5 316nMSS 2 | N3 | Cluster 01 | Secondary | 53.87 |
| RGC5 316nMSS 2 | N4 | Cluster 01 | Secondary | 133.128 |
| RGC5 316nMSS 2 | N5 | Cluster 01 | Primary | 165.87 |
| RGC5 316nMSS 2 | N6 | Cluster 01 | Secondary | 156.419 |
| RGC5 316nMSS 2 | N7 | Cluster 01 | Secondary | 97.894 |
| RGC5 316nMSS 2 | N8 | Cluster 01 | Primary | 176.801 |
| RGC5 316nMSS 2 | N9 | Cluster 02 | Primary | 64.095 |
| RGC5 316nMSS 2 | N10 | Cluster 03 | Primary | 193.739 |
| RGC5 316nMSS 2 | N11 | Cluster 03 | Primary | 92.647 |
| RGC5 316nMSS 2 | N12 | Cluster 03 | Primary | 99.725 |
| RGC5 316nMSS 2 | N13 | Cluster 03 | Secondary | 33.08 |
| RGC5 316nMSS 2 | N14 | Cluster 03 | Secondary | 36.61 |
| RGC5 316nMSS 2 | N15 | Cluster 03 | Primary | 107.889 |
| RGC5 316nMSS 3 | N1 | Cluster 01 | Primary | 91.244 |
| RGC5 316nMSS 3 | N2 | Cluster 01 | Secondary | 46.558 |
| RGC5 316nMSS 3 | N3 | Cluster 01 | Secondary | 55.656 |
| RGC5 316nMSS 3 | N4 | Cluster 01 | Primary | 102.828 |
| RGC5 316nMSS 3 | N5 | Cluster 02 | Primary | 56.207 |
| RGC5 316nMSS 3 | N6 | Cluster 02 | Secondary | 68.877 |
| RGC5 316nMSS 3 | N7 | Cluster 02 | Secondary | 97.945 |
| RGC5 316nMSS 3 | N8 | Cluster 02 | Primary | 101.407 |
| RGC5 316nMSS 4 | N1 | Cluster 01 | Primary | 72.901 |
| RGC5 316nMSS 4 | N4 | Cluster 01 | Secondary | 18.659 |
| RGC5 316nMSS 4 | N5 | Cluster 01 | Secondary | 25.653 |
| RGC5 316nMSS 4 | N6 | Cluster 01 | Tertiary | 13.099 |
| RGC5 316nMSS 4 | N7 | Cluster 01 | Tertiary | 51.502 |
| RGC5 316nMSS 4 | N8 | Cluster 01 | Primary | 75.216 |
| RGC5 316nMSS 4 | N9 | Cluster 01 | Secondary | 39.234 |
| RGC5 316nMSS 4 | N10 | Cluster 01 | Secondary | 64.611 |
| RGC5 316nMSS 4 | N11 | Cluster 01 | Tertiary | 3.162 |
| RGC5 316nMSS 4 | N12 | Cluster 01 | Tertiary | 67.481 |
| RGC5 316nMSS 4 | N13 | Cluster 01 | Primary | 108.918 |
| RGC5 316nMSS 4 | N14 | Cluster 01 | Primary | 91.375 |
| RGC5 316nMSS 4 | N15 | Cluster 01 | Secondary | 46.174 |
| RGC5 316nMSS 4 | N16 | Cluster 01 | Secondary | 38.075 |
| RGC5 316nMSS 4 | N17 | Cluster 01 | Secondary | 55.066 |
| RGC5 316nMSS 4 | N18 | Cluster 01 | Primary | 90.497 |
| RGC5 316nMSS 4 | N19 | Cluster 01 | Primary | 142.872 |
| RGC5 316nMSS 4 | N20 | Cluster 02 | Primary | 91.186 |
| RGC5 316nMSS 4 | N22 | Cluster 02 | Primary | 103.868 |
| RGC5 316nMSS 4 | N23 | Cluster 02 | Primary | 32.371 |
| RGC5 316nMSS 4 | N24 | Cluster 02 | Secondary | 44.816 |
| RGC5 316nMSS 4 | N25 | Cluster 02 | Secondary | 49.132 |
| RGC5 316nMSS 4 | N26 | Cluster 02 | Tertiary | 60.694 |
| RGC5 316nMSS 4 | N27 | Cluster 02 | Tertiary | 51.277 |
| RGC5 316nMSS 4 | N28 | Cluster 02 | Type 06 | 54.63 |
| RGC5 316nMSS 4 | N29 | Cluster 02 | Type 06 | 60.485 |
| RGC5 316nMSS 4 | N30 | Cluster 02 | Primary | 53.115 |
| RGC5 316nMSS 4 | N31 | Cluster 02 | Secondary | 44.27 |
| RGC5 316nMSS 4 | N32 | Cluster 02 | Secondary | 103.894 |
| RGC5 316nMSS 5 | N1 | Cluster 01 | Primary | 55.396 |
| RGC5 316nMSS 5 | N2 | Cluster 01 | Secondary | 68.036 |
| RGC5 316nMSS 5 | N3 | Cluster 01 | Secondary | 29.191 |
| RGC5 316nMSS 5 | N4 | Cluster 01 | Primary | 22.432 |
| RGC5 316nMSS 5 | N5 | Cluster 01 | Secondary | 62.482 |
| RGC5 316nMSS 5 | N6 | Cluster 01 | Secondary | 69.655 |
| RGC5 316nMSS 5 | N7 | Cluster 01 | Primary | 31.75 |
| RGC5 316nMSS 5 | N8 | Cluster 01 | Secondary | 131.82 |
| RGC5 316nMSS 5 | N9 | Cluster 01 | Secondary | 109.701 |
| RGC5 316nMSS 5 | N10 | Cluster 02 | Primary | 63.959 |
| RGC5 316nMSS 5 | N11 | Cluster 03 | Primary | 37.869 |

| | | | | |
|---|---|---|---|---|
| RGC5 316nMSS 5 | N12 | Cluster 03 | Secondary | 69.086 |
| RGC5 316nMSS 5 | N13 | Cluster 03 | Secondary | 76.028 |
| RGC5 316nMSS 5 | N14 | Cluster 04 | Primary | 81.558 |
| RGC5 316nMSS 5 | N15 | Cluster 04 | Primary | 33.869 |
| RGC5 316nMSS 5 | N16 | Cluster 04 | Primary | 142.452 |
| RGC5 316nMSS 5 | N17 | Cluster 05 | Primary | 203.647 |
| RGC5 316nMSS 5 | N18 | Cluster 05 | Primary | 114.706 |
| RGC5 316nMSS 5 | N19 | Cluster 05 | Secondary | 52.375 |
| RGC5 316nMSS 5 | N20 | Cluster 05 | Secondary | 57.669 |
| RGC5 316nMSS 5 | N21 | Cluster 05 | Primary | 60.256 |
| RGC5 316nMSS 5 | N22 | Cluster 05 | Secondary | 38.413 |
| RGC5 316nMSS 5 | N23 | Cluster 05 | Secondary | 35.29 |
| RGC5 316nMSS 6 | N1 | Cluster 01 | Primary | 152.924 |
| RGC5 316nMSS 6 | N2 | Cluster 01 | Secondary | 70.078 |
| RGC5 316nMSS 6 | N3 | Cluster 01 | Secondary | 90.993 |
| RGC5 316nMSS 6 | N4 | Cluster 01 | Primary | 94.411 |
| RGC5 316nMSS 6 | N5 | Cluster 01 | Primary | 81.73 |
| RGC5 316nMSS 6 | N7 | Cluster 01 | Primary | 98.069 |
| RGC5 316nMSS 6 | N8 | Cluster 02 | Primary | 24.222 |
| RGC5 316nMSS 6 | N9 | Cluster 02 | Secondary | 104.864 |
| RGC5 316nMSS 6 | N10 | Cluster 02 | Secondary | 92.155 |
| RGC5 316nMSS 6 | N11 | Cluster 02 | Primary | 110.639 |
| RGC5 316nMSS 6 | N12 | Cluster 02 | Secondary | 36.338 |
| RGC5 316nMSS 6 | N13 | Cluster 02 | Secondary | 38.576 |
| RGC5 316nMSS 6 | N14 | Cluster 02 | Tertiary | 47.471 |
| RGC5 316nMSS 6 | N15 | Cluster 02 | Tertiary | 65.641 |
| RGC5 316nMSS 6 | N16 | Cluster 03 | Primary | 124.203 |
| RGC5 316nMSS 6 | N17 | Cluster 03 | Primary | 120.546 |
| RGC5 316nMSS 6 | N18 | Cluster 03 | Primary | 196.644 |
| RGC5 1umSS 1 | N1 | Cluster 01 | Primary | 30.386 |
| RGC5 1umSS 1 | N2 | Cluster 01 | Primary | 46.25 |
| RGC5 1umSS 1 | N3 | Cluster 01 | Secondary | 36.244 |
| RGC5 1umSS 1 | N4 | Cluster 01 | Secondary | 23.987 |
| RGC5 1umSS 1 | N5 | Cluster 01 | Primary | 45.309 |
| RGC5 1umSS 1 | N6 | Cluster 01 | Primary | 61.322 |
| RGC5 1umSS 1 | N7 | Cluster 01 | Secondary | 24.332 |
| RGC5 1umSS 1 | N8 | Cluster 01 | Tertiary | 22.968 |
| RGC5 1umSS 1 | N9 | Cluster 01 | Tertiary | 8.422 |
| RGC5 1umSS 1 | N10 | Cluster 01 | Secondary | 58.401 |
| RGC5 1umSS 1 | N11 | Cluster 01 | Tertiary | 96.246 |
| RGC5 1umSS 1 | N12 | Cluster 01 | Tertiary | 48.864 |
| RGC5 1umSS 1 | N13 | Cluster 01 | Type 06 | 35.658 |
| RGC5 1umSS 1 | N14 | Cluster 01 | Type 06 | 44.486 |
| RGC5 1umSS 1 | N15 | Cluster 02 | Primary | 111.725 |
| RGC5 1umSS 1 | N16 | Cluster 02 | Secondary | 85.725 |
| RGC5 1umSS 1 | N17 | Cluster 02 | Tertiary | 57.42 |
| RGC5 1umSS 1 | N18 | Cluster 02 | Tertiary | 24.706 |
| RGC5 1umSS 1 | N19 | Cluster 02 | Type 06 | 27.056 |
| RGC5 1umSS 1 | N20 | Cluster 02 | Type 06 | 69.697 |
| RGC5 1umSS 1 | N21 | Cluster 02 | Secondary | 75.734 |
| RGC5 1umSS 1 | N22 | Cluster 02 | Tertiary | 91.115 |
| RGC5 1umSS 1 | N23 | Cluster 02 | Tertiary | 45.18 |
| RGC5 1umSS 1 | N24 | Cluster 02 | Type 06 | 84.938 |
| RGC5 1umSS 1 | N25 | Cluster 02 | Type 06 | 39.189 |
| RGC5 1umSS 1 | N26 | Cluster 02 | Primary | 52.195 |
| RGC5 1umSS 1 | N27 | Cluster 02 | Secondary | 25.072 |
| RGC5 1umSS 1 | N28 | Cluster 02 | Secondary | 24.664 |
| RGC5 1umSS 1 | N29 | Cluster 02 | Primary | 41.099 |
| RGC5 1umSS 1 | N30 | Cluster 02 | Secondary | 45.592 |
| RGC5 1umSS 1 | N31 | Cluster 02 | Secondary | 23.483 |
| RGC5 1umSS 1 | N32 | Cluster 02 | Tertiary | 50.981 |
| RGC5 1umSS 1 | N33 | Cluster 02 | Tertiary | 33.261 |
| RGC5 1umSS 1 | N34 | Cluster 02 | Primary | 101.222 |
| RGC5 1umSS 1 | N35 | Cluster 02 | Secondary | 27.022 |
| RGC5 1umSS 1 | N36 | Cluster 02 | Secondary | 12.222 |
| RGC5 1umSS 1 | N37 | Cluster 02 | Tertiary | 40.248 |
| RGC5 1umSS 1 | N38 | Cluster 02 | Tertiary | 38.966 |
| RGC5 1umSS 1 | N39 | Cluster 02 | Primary | 17.216 |
| RGC5 1umSS 1 | N40 | Cluster 02 | Secondary | 81.125 |
| RGC5 1umSS 1 | N41 | Cluster 02 | Secondary | 123.423 |
| RGC5 1umSS 1 | N42 | Cluster 03 | Primary | 31.835 |
| RGC5 1umSS 1 | N43 | Cluster 03 | Secondary | 71.107 |
| RGC5 1umSS 1 | N44 | Cluster 03 | Secondary | 88.181 |
| RGC5 1umSS 1 | N45 | Cluster 03 | Primary | 57.236 |
| RGC5 1umSS 1 | N46 | Cluster 03 | Primary | 17.222 |
| RGC5 1umSS 1 | N47 | Cluster 03 | Secondary | 30.255 |
| RGC5 1umSS 1 | N48 | Cluster 03 | Secondary | 28.017 |
| RGC5 1umSS 1 | N49 | Cluster 03 | Primary | 45.811 |

| | | | | |
|---|---|---|---|---|
| RGC5 1umSS 1 | N50 | Cluster 03 | Secondary | 19.316 |
| RGC5 1umSS 1 | N51 | Cluster 03 | Secondary | 18.463 |
| RGC5 1umSS 1 | N52 | Cluster 03 | Primary | 23.402 |
| RGC5 1umSS 1 | N53 | Cluster 03 | Secondary | 28.549 |
| RGC5 1umSS 1 | N54 | Cluster 03 | Secondary | 24.061 |
| RGC5 1umSS 1 | N55 | Cluster 03 | Tertiary | 21.508 |
| RGC5 1umSS 1 | N56 | Cluster 03 | Tertiary | 26.109 |
| RGC5 1umSS 1 | N58 | Cluster 03 | Primary | 51.973 |
| RGC5 1umSS 1 | N59 | Cluster 03 | Secondary | 45.98 |
| RGC5 1umSS 1 | N60 | Cluster 03 | Tertiary | 40.931 |
| RGC5 1umSS 1 | N61 | Cluster 03 | Type 06 | 41.091 |
| RGC5 1umSS 1 | N62 | Cluster 03 | Type 06 | 33.853 |
| RGC5 1umSS 1 | N63 | Cluster 03 | Tertiary | 42.432 |
| RGC5 1umSS 1 | N64 | Cluster 03 | Tertiary | 31.601 |
| RGC5 1umSS 1 | N65 | Cluster 03 | Secondary | 24.519 |
| RGC5 1umSS 1 | N66 | Cluster 03 | Primary | 126.168 |
| RGC5 1umSS 1 | N67 | Cluster 03 | Secondary | 51.265 |
| RGC5 1umSS 1 | N68 | Cluster 03 | Secondary | 64.066 |
| RGC5 1umSS 1 | N69 | Cluster 03 | Tertiary | 26.715 |
| RGC5 1umSS 1 | N70 | Cluster 03 | Tertiary | 31.869 |
| RGC5 1umSS 1 | N71 | Cluster 04 | Primary | 18.37 |
| RGC5 1umSS 1 | N72 | Cluster 02 | Secondary | 31.799 |
| RGC5 1umSS 1 | N73 | Cluster 04 | Secondary | 26.656 |
| RGC5 1umSS 1 | N74 | Cluster 04 | Tertiary | 26.068 |
| RGC5 1umSS 1 | N75 | Cluster 04 | Tertiary | 74.261 |
| RGC5 1umSS 1 | N76 | Cluster 04 | Type 06 | 25.013 |
| RGC5 1umSS 1 | N77 | Cluster 04 | Type 06 | 67.058 |
| RGC5 1umSS 1 | N78 | Cluster 04 | Primary | 30.565 |
| RGC5 1umSS 1 | N79 | Cluster 04 | Secondary | 18.261 |
| RGC5 1umSS 1 | N80 | Cluster 04 | Secondary | 38.566 |
| RGC5 1umSS 1 | N81 | Cluster 04 | Primary | 49.404 |
| RGC5 1umSS 1 | N82 | Cluster 04 | Primary | 21.986 |
| RGC5 1umSS 1 | N83 | Cluster 04 | Secondary | 96.567 |
| RGC5 1umSS 1 | N84 | Cluster 04 | Secondary | 34.358 |
| RGC5 1umSS 1 | N85 | Cluster 05 | Primary | 131.808 |
| RGC5 1umSS 1 | N86 | Cluster 05 | Primary | 89.377 |
| RGC5 1umSS 1 | N87 | Cluster 05 | Secondary | 73.558 |
| RGC5 1umSS 1 | N88 | Cluster 05 | Secondary | 65.282 |
| RGC5 1umSS 1 | N89 | Cluster 05 | Primary | 17.214 |
| RGC5 1umSS 1 | N90 | Cluster 05 | Secondary | 83.562 |
| RGC5 1umSS 1 | N91 | Cluster 05 | Secondary | 31.28 |
| RGC5 1umSS 1 | N92 | Cluster 05 | Tertiary | 76.758 |
| RGC5 1umSS 1 | N93 | Cluster 05 | Tertiary | 67.374 |
| RGC5 1umSS 1 | N94 | Cluster 05 | Type 06 | 46.244 |
| RGC5 1umSS 1 | N95 | Cluster 05 | Type 06 | 69.147 |
| RGC5 1umSS 1 | N96 | Cluster 05 | Primary | 80.888 |
| RGC5 1umSS 1 | N97 | Cluster 05 | Secondary | 94.206 |
| RGC5 1umSS 1 | N98 | Cluster 05 | Secondary | 80.922 |
| RGC5 1umSS 1 | N99 | Cluster 06 | Primary | 26.105 |
| RGC5 1umSS 1 | N100 | Cluster 06 | Secondary | 23.958 |
| RGC5 1umSS 1 | N101 | Cluster 06 | Secondary | 109.692 |
| RGC5 1umSS 1 | N102 | Cluster 06 | Primary | 27.533 |
| RGC5 1umSS 1 | N103 | Cluster 06 | Secondary | 14.376 |
| RGC5 1umSS 1 | N104 | Cluster 06 | Tertiary | 25.545 |
| RGC5 1umSS 1 | N105 | Cluster 06 | Type 06 | 27.628 |
| RGC5 1umSS 1 | N106 | Cluster 06 | Type 06 | 12.285 |
| RGC5 1umSS 1 | N107 | Cluster 06 | Type 07 | 28.848 |
| RGC5 1umSS 1 | N108 | Cluster 06 | Type 07 | 33.467 |
| RGC5 1umSS 1 | N109 | Cluster 06 | Tertiary | 54.102 |
| RGC5 1umSS 1 | N110 | Cluster 06 | Type 06 | 18.637 |
| RGC5 1umSS 1 | N111 | Cluster 06 | Type 06 | 43.709 |
| RGC5 1umSS 1 | N112 | Cluster 06 | Type 07 | 23.945 |
| RGC5 1umSS 1 | N113 | Cluster 06 | Type 07 | 41.681 |
| RGC5 1umSS 1 | N114 | Cluster 06 | Primary | 73.52 |
| RGC5 1umSS 1 | N115 | Cluster 06 | Secondary | 35.441 |
| RGC5 1umSS 1 | N116 | Cluster 06 | Tertiary | 17.261 |
| RGC5 1umSS 1 | N117 | Cluster 06 | Tertiary | 32.911 |
| RGC5 1umSS 1 | N118 | Cluster 06 | Secondary | 26.887 |
| RGC5 1umSS 1 | N119 | Cluster 06 | Primary | 57.514 |
| RGC5 1umSS 1 | N120 | Cluster 06 | Primary | 68.301 |
| RGC5 1umSS 1 | N121 | Cluster 06 | Primary | 41.166 |
| RGC5 1umSS 1 | N122 | Cluster 06 | Secondary | 49.23 |
| RGC5 1umSS 1 | N123 | Cluster 06 | Secondary | 59.774 |
| RGC5 1umSS 1 | N124 | Cluster 06 | Secondary | 38.202 |
| RGC5 1umSS 2 | N1 | Cluster 01 | Primary | 120.857 |
| RGC5 1umSS 2 | N2 | Cluster 01 | Secondary | 108.878 |
| RGC5 1umSS 2 | N3 | Cluster 01 | Secondary | 55.846 |
| RGC5 1umSS 2 | N4 | Cluster 01 | Primary | 74.714 |

| | | | | |
|---|---|---|---|---|
| RGC5 1umSS 2 | N5 | Cluster 01 | Secondary | 109.765 |
| RGC5 1umSS 2 | N6 | Cluster 01 | Tertiary | 57.19 |
| RGC5 1umSS 2 | N7 | Cluster 01 | Tertiary | 88.584 |
| RGC5 1umSS 2 | N8 | Cluster 01 | Primary | 52.842 |
| RGC5 1umSS 2 | N9 | Cluster 01 | Secondary | 25.209 |
| RGC5 1umSS 2 | N10 | Cluster 01 | Secondary | 24.69 |
| RGC5 1umSS 2 | N11 | Cluster 01 | Primary | 124.761 |
| RGC5 1umSS 2 | N12 | Cluster 01 | Secondary | 45.13 |
| RGC5 1umSS 2 | N13 | Cluster 01 | Secondary | 99.061 |
| RGC5 1umSS 2 | N14 | Cluster 01 | Tertiary | 78.466 |
| RGC5 1umSS 2 | N15 | Cluster 01 | Tertiary | 55.344 |
| RGC5 1umSS 2 | N16 | Cluster 01 | Type 06 | 102.201 |
| RGC5 1umSS 2 | N17 | Cluster 01 | Type 06 | 41.546 |
| RGC5 1umSS 2 | N18 | Cluster 01 | Primary | 52.911 |
| RGC5 1umSS 2 | N19 | Cluster 01 | Secondary | 61.42 |
| RGC5 1umSS 2 | N20 | Cluster 01 | Tertiary | 93.781 |
| RGC5 1umSS 2 | N21 | Cluster 01 | Secondary | 58.276 |
| RGC5 1umSS 2 | N22 | Cluster 01 | Tertiary | 25.869 |
| RGC5 1umSS 2 | N23 | Cluster 01 | Tertiary | 19.822 |
| RGC5 1umSS 2 | N24 | Cluster 01 | Primary | 35.31 |
| RGC5 1umSS 2 | N25 | Cluster 01 | Secondary | 133.886 |
| RGC5 1umSS 2 | N26 | Cluster 01 | Secondary | 84.662 |
| RGC5 1umSS 2 | N27 | Cluster 01 | Tertiary | 56.981 |
| RGC5 1umSS 2 | N28 | Cluster 01 | Tertiary | 58.683 |
| RGC5 1umSS 2 | N29 | Cluster 01 | Type 06 | 99.904 |
| RGC5 1umSS 2 | N30 | Cluster 01 | Type 06 | 27.544 |
| RGC5 1umSS 2 | N31 | Cluster 02 | Primary | 172.093 |
| RGC5 1umSS 2 | N32 | Cluster 02 | Primary | 37.047 |
| RGC5 1umSS 2 | N33 | Cluster 02 | Secondary | 75.161 |
| RGC5 1umSS 2 | N34 | Cluster 02 | Secondary | 59.912 |
| RGC5 1umSS 2 | N35 | Cluster 02 | Tertiary | 79.56 |
| RGC5 1umSS 2 | N36 | Cluster 02 | Tertiary | 29.567 |
| RGC5 1umSS 2 | N37 | Cluster 02 | Type 06 | 36.634 |
| RGC5 1umSS 2 | N38 | Cluster 02 | Type 06 | 38.263 |
| RGC5 1umSS 2 | N39 | Cluster 02 | Type 07 | 37.29 |
| RGC5 1umSS 2 | N40 | Cluster 02 | Type 07 | 81.285 |
| RGC5 1umSS 2 | N41 | Cluster 02 | Type 08 | 52.249 |
| RGC5 1umSS 2 | N42 | Cluster 02 | Type 08 | 40.82 |
| RGC5 1umSS 2 | N43 | Cluster 02 | Primary | 197.399 |
| RGC5 1umSS 2 | N44 | Cluster 02 | Secondary | 25.7 |
| RGC5 1umSS 2 | N45 | Cluster 02 | Tertiary | 100.975 |
| RGC5 1umSS 2 | N46 | Cluster 02 | Tertiary | 79.398 |
| RGC5 1umSS 2 | N47 | Cluster 02 | Secondary | 18.646 |
| RGC5 1umSS 2 | N48 | Cluster 02 | Tertiary | 34.938 |
| RGC5 1umSS 2 | N49 | Cluster 02 | Tertiary | 39.538 |
| RGC5 1umSS 2 | N50 | Cluster 02 | Primary | 63.301 |
| RGC5 1umSS 2 | N51 | Cluster 02 | Secondary | 64.706 |
| RGC5 1umSS 2 | N52 | Cluster 02 | Secondary | 47.717 |
| RGC5 1umSS 2 | N53 | Cluster 02 | Primary | 67.016 |
| RGC5 1umSS 2 | N54 | Cluster 02 | Secondary | 50.355 |
| RGC5 1umSS 2 | N55 | Cluster 02 | Secondary | 50.496 |
| RGC5 1umSS 2 | N56 | Cluster 03 | Primary | 90.047 |
| RGC5 1umSS 2 | N57 | Cluster 03 | Secondary | 30.601 |
| RGC5 1umSS 2 | N58 | Cluster 03 | Secondary | 37.647 |
| RGC5 1umSS 2 | N59 | Cluster 03 | Primary | 76.814 |
| RGC5 1umSS 2 | N60 | Cluster 03 | Secondary | 16.971 |
| RGC5 1umSS 2 | N61 | Cluster 03 | Secondary | 64.207 |
| RGC5 1umSS 2 | N62 | Cluster 03 | Secondary | 65.428 |
| RGC5 1umSS 2 | N63 | Cluster 03 | Primary | 81.448 |
| RGC5 1umSS 2 | N64 | Cluster 03 | Primary | 151.853 |
| RGC5 1umSS 2 | N65 | Cluster 03 | Primary | 92.299 |
| RGC5 1umSS 2 | N66 | Cluster 03 | Primary | 71.46 |
| RGC5 1umSS 2 | N67 | Cluster 03 | Secondary | 13.177 |
| RGC5 1umSS 2 | N68 | Cluster 03 | Secondary | 68.211 |
| RGC5 1umSS 2 | N69 | Cluster 03 | Tertiary | 33.489 |
| RGC5 1umSS 2 | N70 | Cluster 03 | Tertiary | 30.537 |
| RGC5 1umSS 2 | N71 | Cluster 03 | Primary | 43.32 |
| RGC5 1umSS 2 | N72 | Cluster 03 | Secondary | 39.278 |
| RGC5 1umSS 2 | N73 | Cluster 03 | Secondary | 68.812 |
| RGC5 1umSS 2 | N74 | Cluster 03 | Tertiary | 31.119 |
| RGC5 1umSS 2 | N75 | Cluster 03 | Tertiary | 40.959 |
| RGC5 1umSS 2 | N76 | Cluster 03 | Tertiary | 39.779 |
| RGC5 1umSS 2 | N77 | Cluster 03 | Primary | 58.269 |
| RGC5 1umSS 2 | N78 | Cluster 03 | Secondary | 45.926 |
| RGC5 1umSS 2 | N79 | Cluster 03 | Secondary | 36.04 |
| RGC5 1umSS 2 | N80 | Cluster 04 | Primary | 22.438 |
| RGC5 1umSS 2 | N81 | Cluster 04 | Secondary | 29.873 |
| RGC5 1umSS 2 | N82 | Cluster 04 | Tertiary | 35.238 |

| | | | | |
|---|---|---|---|---|
| RGC5 1umSS 2 | N83 | Cluster 04 | Tertiary | 36.612 |
| RGC5 1umSS 2 | N84 | Cluster 04 | Type 06 | 32.054 |
| RGC5 1umSS 2 | N85 | Cluster 04 | Type 06 | 32.341 |
| RGC5 1umSS 2 | N87 | Cluster 04 | Primary | 134.442 |
| RGC5 1umSS 2 | N89 | Cluster 04 | Secondary | 43.189 |
| RGC5 1umSS 2 | N91 | Cluster 04 | Primary | 95.64 |
| RGC5 1umSS 2 | N92 | Cluster 04 | Secondary | 39.673 |
| RGC5 1umSS 2 | N93 | Cluster 04 | Secondary | 43.842 |
| RGC5 1umSS 2 | N94 | Cluster 04 | Primary | 182.715 |
| RGC5 1umSS 3 | N1 | Cluster 01 | Primary | 52.265 |
| RGC5 1umSS 3 | N2 | Cluster 01 | Secondary | 48.864 |
| RGC5 1umSS 3 | N3 | Cluster 01 | Secondary | 59.446 |
| RGC5 1umSS 3 | N4 | Cluster 01 | Primary | 54.47 |
| RGC5 1umSS 3 | N5 | Cluster 01 | Secondary | 86.352 |
| RGC5 1umSS 3 | N6 | Cluster 02 | Tertiary | 39.562 |
| RGC5 1umSS 3 | N7 | Cluster 01 | Tertiary | 67.036 |
| RGC5 1umSS 3 | N8 | Cluster 01 | Secondary | 9.437 |
| RGC5 1umSS 3 | N9 | Cluster 01 | Tertiary | 34.666 |
| RGC5 1umSS 3 | N10 | Cluster 01 | Tertiary | 19.893 |
| RGC5 1umSS 3 | N11 | Cluster 01 | Primary | 98.684 |
| RGC5 1umSS 3 | N13 | Cluster 02 | Primary | 42.283 |
| RGC5 1umSS 3 | N14 | Cluster 02 | Secondary | 47.645 |
| RGC5 1umSS 3 | N15 | Cluster 02 | Secondary | 58.188 |
| RGC5 1umSS 3 | N16 | Cluster 02 | Primary | 57.707 |
| RGC5 1umSS 3 | N17 | Cluster 02 | Secondary | 77.025 |
| RGC5 1umSS 3 | N18 | Cluster 02 | Tertiary | 108.177 |
| RGC5 1umSS 3 | N19 | Cluster 02 | Tertiary | 22.911 |
| RGC5 1umSS 3 | N20 | Cluster 02 | Secondary | 72.704 |
| RGC5 1umSS 3 | N21 | Cluster 02 | Primary | 70.591 |
| RGC5 1umSS 3 | N22 | Cluster 03 | Primary | 71.059 |
| RGC5 1umSS 3 | N23 | Cluster 03 | Primary | 59.925 |
| RGC5 1umSS 3 | N24 | Cluster 03 | Secondary | 38.81 |
| RGC5 1umSS 3 | N25 | Cluster 03 | Secondary | 23.965 |
| RGC5 1umSS 3 | N26 | Cluster 03 | Primary | 89.027 |
| RGC5 1umSS 3 | N27 | Cluster 04 | Primary | 55.945 |
| RGC5 1umSS 3 | N28 | Cluster 04 | Secondary | 31.986 |
| RGC5 1umSS 3 | N29 | Cluster 04 | Secondary | 34.475 |
| RGC5 1umSS 3 | N30 | Cluster 04 | Primary | 73.109 |
| RGC5 1umSS 3 | N35 | Cluster 05 | Primary | 180.549 |
| RGC5 1umSS 3 | N36 | Cluster 05 | Primary | 155.139 |
| RGC5 1umSS 3 | N37 | Cluster 05 | Primary | 86.402 |
| RGC5 1umSS 3 | N38 | Cluster 05 | Secondary | 65.26 |
| RGC5 1umSS 3 | N39 | Cluster 05 | Tertiary | 46.934 |
| RGC5 1umSS 3 | N40 | Cluster 05 | Tertiary | 37.272 |
| RGC5 1umSS 3 | N41 | Cluster 05 | Type 06 | 146.814 |
| RGC5 1umSS 3 | N42 | Cluster 05 | Type 06 | 24.12 |
| RGC5 1umSS 3 | N43 | Cluster 05 | Secondary | 35.336 |
| RGC5 1umSS 3 | N44 | Cluster 05 | Tertiary | 126.885 |
| RGC5 1umSS 3 | N45 | Cluster 05 | Tertiary | 53.897 |
| RGC5 1umSS 3 | N46 | Cluster 05 | Primary | 21.357 |
| RGC5 1umSS 3 | N47 | Cluster 05 | Secondary | 41.943 |
| RGC5 1umSS 3 | N48 | Cluster 05 | Secondary | 41.753 |
| RGC5 1umSS 3 | N49 | Cluster 05 | Tertiary | 37.911 |
| RGC5 1umSS 3 | N50 | Cluster 05 | Tertiary | 46.156 |
| RGC5 1umSS 3 | N51 | Cluster 05 | Tertiary | 39.973 |
| RGC5 1umSS 3 | N52 | Cluster 05 | Primary | 107.143 |
| RGC5 1umSS 4 | N1 | Cluster 01 | Primary | 148.946 |
| RGC5 1umSS 4 | N2 | Cluster 02 | Primary | 105.086 |
| RGC5 1umSS 4 | N3 | Cluster 02 | Primary | 109.871 |
| RGC5 1umSS 4 | N4 | Cluster 02 | Primary | 177.149 |
| RGC5 1umSS 4 | N5 | Cluster 04 | Primary | 88.19 |
| RGC5 1umSS 4 | N6 | Cluster 05 | Primary | 33.411 |
| RGC5 1umSS 4 | N7 | Cluster 05 | Secondary | 73.411 |
| RGC5 1umSS 4 | N8 | Cluster 05 | Secondary | 252.265 |
| RGC5 1umSS 4 | N9 | Cluster 05 | Primary | 67.613 |
| RGC5 1umSS 4 | N10 | Cluster 05 | Primary | 188.639 |
| RGC5 1umSS 5 | N1 | Cluster 01 | Primary | 75.428 |
| RGC5 1umSS 5 | N2 | Cluster 01 | Secondary | 40.853 |
| RGC5 1umSS 5 | N3 | Cluster 01 | Secondary | 34.789 |
| RGC5 1umSS 5 | N4 | Cluster 01 | Primary | 53.737 |
| RGC5 1umSS 5 | N5 | Cluster 01 | Secondary | 13.474 |
| RGC5 1umSS 5 | N6 | Cluster 01 | Secondary | 35.238 |
| RGC5 1umSS 5 | N7 | Cluster 01 | Tertiary | 61.722 |
| RGC5 1umSS 5 | N8 | Cluster 01 | Type 06 | 26.255 |
| RGC5 1umSS 5 | N9 | Cluster 01 | Type 06 | 59.873 |
| RGC5 1umSS 5 | N10 | Cluster 01 | Type 07 | 31.739 |
| RGC5 1umSS 5 | N11 | Cluster 01 | Type 07 | 32.245 |
| RGC5 1umSS 5 | N12 | Cluster 01 | Type 07 | 34.612 |

| | | | | |
|---|---|---|---|---|
| RGC5 1umSS 5 | N13 | Cluster 01 | Tertiary | 153.242 |
| RGC5 1umSS 5 | N14 | Cluster 01 | Primary | 126.902 |
| RGC5 1umSS 5 | N15 | Cluster 01 | Secondary | 76.063 |
| RGC5 1umSS 5 | N16 | Cluster 01 | Secondary | 56.512 |
| RGC5 1umSS 5 | N17 | Cluster 01 | Primary | 38.889 |
| RGC5 1umSS 5 | N18 | Cluster 01 | Secondary | 282.615 |
| RGC5 1umSS 5 | N19 | Cluster 01 | Tertiary | 34.841 |
| RGC5 1umSS 5 | N20 | Cluster 01 | Tertiary | 99.281 |
| RGC5 1umSS 5 | N21 | Cluster 02 | Primary | 94.319 |
| RGC5 1umSS 5 | N22 | Cluster 02 | Primary | 74.261 |
| RGC5 1umSS 5 | N23 | Cluster 02 | Primary | 53.615 |
| RGC5 1umSS 5 | N24 | Cluster 02 | Secondary | 37.425 |
| RGC5 1umSS 5 | N25 | Cluster 02 | Secondary | 91.66 |
| RGC5 1umSS 5 | N26 | Cluster 03 | Primary | 103.969 |
| RGC5 1umSS 5 | N27 | Cluster 03 | Primary | 121.972 |
| RGC5 1umSS 5 | N28 | Cluster 03 | Secondary | 55.371 |
| RGC5 1umSS 5 | N29 | Cluster 03 | Secondary | 53.378 |
| RGC5 1umSS 5 | N30 | Cluster 03 | Secondary | 25.448 |
| RGC5 1umSS 5 | N31 | Cluster 03 | Tertiary | 32.968 |
| RGC5 1umSS 5 | N32 | Cluster 03 | Tertiary | 67.867 |
| RGC5 1umSS 5 | N33 | Cluster 03 | Primary | 64.267 |
| RGC5 1umSS 5 | N34 | Cluster 03 | Secondary | 31.316 |
| RGC5 1umSS 5 | N35 | Cluster 03 | Secondary | 49.909 |
| RGC5 1umSS 5 | N36 | Cluster 03 | Primary | 123.05 |
| RGC5 1umSS 5 | N37 | Cluster 03 | Secondary | 80.872 |
| RGC5 1umSS 5 | N38 | Cluster 03 | Secondary | 109.319 |
| RGC5 1umSS 5 | N39 | Cluster 03 | Primary | 40.166 |
| RGC5 1umSS 5 | N40 | Cluster 03 | Secondary | 43.496 |
| RGC5 1umSS 5 | N41 | Cluster 03 | Secondary | 111.527 |
| RGC5 1umSS 5 | N42 | Cluster 03 | Tertiary | 90.769 |
| RGC5 1umSS 5 | N43 | Cluster 03 | Tertiary | 44.288 |
| RGC5 1umSS 5 | N44 | Cluster 04 | Primary | 60.102 |
| RGC5 1umSS 5 | N45 | Cluster 04 | Secondary | 55.906 |
| RGC5 1umSS 5 | N46 | Cluster 04 | Secondary | 26.835 |
| RGC5 1umSS 5 | N47 | Cluster 04 | Primary | 50.112 |
| RGC5 1umSS 5 | N48 | Cluster 04 | Secondary | 62.578 |
| RGC5 1umSS 5 | N49 | Cluster 04 | Secondary | 62.944 |
| RGC5 1umSS 5 | N50 | Cluster 04 | Tertiary | 82.182 |
| RGC5 1umSS 5 | N51 | Cluster 04 | Tertiary | 26.376 |
| RGC5 1umSS 5 | N52 | Cluster 04 | Primary | 40.19 |
| RGC5 1umSS 5 | N53 | Cluster 04 | Secondary | 75.93 |
| RGC5 1umSS 5 | N54 | Cluster 04 | Tertiary | 35.495 |
| RGC5 1umSS 5 | N55 | Cluster 04 | Type 06 | 47.214 |
| RGC5 1umSS 5 | N56 | Cluster 04 | Type 06 | 43.479 |
| RGC5 1umSS 5 | N57 | Cluster 04 | Tertiary | 90.481 |
| RGC5 1umSS 5 | N58 | Cluster 04 | Secondary | 47.329 |
| RGC5 1umSS 5 | N59 | Cluster 05 | Primary | 101.597 |
| RGC5 1umSS 5 | N60 | Cluster 05 | Secondary | 83.636 |
| RGC5 1umSS 5 | N61 | Cluster 05 | Secondary | 111.932 |
| RGC5 1umSS 5 | N62 | Cluster 05 | Primary | 83.254 |
| RGC5 1umSS 5 | N63 | Cluster 05 | Secondary | 41.278 |
| RGC5 1umSS 5 | N64 | Cluster 05 | Secondary | 59.736 |
| RGC5 1umSS 5 | N65 | Cluster 05 | Primary | 66.799 |
| RGC5 1umSS 5 | N66 | Cluster 05 | Secondary | 31.354 |
| RGC5 1umSS 5 | N67 | Cluster 05 | Secondary | 22.527 |
| RGC5 1umSS 5 | N68 | Cluster 05 | Secondary | 26.326 |
| RGC5 1umSS 5 | N70 | Cluster 05 | Tertiary | 16.971 |
| RGC5 1umSS 5 | N71 | Cluster 05 | Tertiary | 36.089 |
| RGC5 1umSS 5 | N72 | Cluster 05 | Tertiary | 125.65 |
| RGC5 1umSS 6 | N1 | Cluster 01 | Primary | 86.037 |
| RGC5 1umSS 6 | N2 | Cluster 01 | Secondary | 50.723 |
| RGC5 1umSS 6 | N3 | Cluster 01 | Secondary | 95.224 |
| RGC5 1umSS 6 | N4 | Cluster 01 | Secondary | 51.849 |
| RGC5 1umSS 6 | N5 | Cluster 01 | Tertiary | 92.462 |
| RGC5 1umSS 6 | N6 | Cluster 02 | Primary | 102.181 |
| RGC5 1umSS 6 | N7 | Cluster 01 | Primary | 44.808 |
| RGC5 1umSS 6 | N8 | Cluster 01 | Secondary | 40.443 |
| RGC5 1umSS 6 | N9 | Cluster 01 | Secondary | 23.852 |
| RGC5 1umSS 6 | N10 | Cluster 01 | Tertiary | 23.386 |
| RGC5 1umSS 6 | N11 | Cluster 01 | Tertiary | 48.349 |
| RGC5 1umSS 6 | N12 | Cluster 01 | Tertiary | 44.711 |
| RGC5 1umSS 6 | N13 | Cluster 01 | Primary | 46.77 |
| RGC5 1umSS 6 | N14 | Cluster 01 | Secondary | 48.449 |
| RGC5 1umSS 6 | N15 | Cluster 01 | Secondary | 46.057 |
| RGC5 1umSS 6 | N16 | Cluster 01 | Primary | 35.371 |
| RGC5 1umSS 6 | N17 | Cluster 01 | Secondary | 42.533 |
| RGC5 1umSS 6 | N18 | Cluster 01 | Tertiary | 19.727 |
| RGC5 1umSS 6 | N19 | Cluster 01 | Tertiary | 36.793 |

| | | | | |
|---|---|---|---|---|
| RGC5 1umSS 6 | N20 | Cluster 01 | Tertiary | 108.989 |
| RGC5 1umSS 6 | N21 | Cluster 01 | Secondary | 37.841 |
| RGC5 1umSS 6 | N22 | Cluster 02 | Primary | 170.57 |
| RGC5 1umSS 6 | N23 | Cluster 02 | Primary | 176.308 |
| RGC5 1umSS 6 | N24 | Cluster 02 | Secondary | 47.955 |
| RGC5 1umSS 6 | N25 | Cluster 02 | Secondary | 56.164 |
| RGC5 1umSS 6 | N26 | Cluster 02 | Tertiary | 59.307 |
| RGC5 1umSS 6 | N27 | Cluster 02 | Tertiary | 127.994 |
| RGC5 1umSS 6 | N29 | Cluster 03 | Primary | 29.237 |
| RGC5 1umSS 6 | N30 | Cluster 03 | Secondary | 26.376 |
| RGC5 1umSS 6 | N31 | Cluster 03 | Secondary | 60.3 |

| Picture | Cell | Total Neurite Field (Pixels) | Branch Points |
|---|---|---|---|
| 661W 0 1 | 1 | 0 | 0 |
| | 2 | 105.631 | 0 |
| | 3 | 0 | 0 |
| | 4 | 0 | 0 |
| | 5 | 0 | 0 |
| | 6 | 0 | 0 |
| | 7 | 0 | 0 |
| | 8 | 209.152 | 0 |
| | 9 | 0 | 0 |
| | 10 | 0 | 0 |
| 661W 0 2 | 1 | 0 | 0 |
| | 2 | 0 | 0 |
| | 3 | 113.377 | 0 |
| | 4 | 0 | 0 |
| | 5 | 0 | 0 |
| 661W 0 3 | 1 | 0 | 0 |
| | 2 | 0 | 0 |
| | 3 | 243.588 | 0 |
| | 4 | 0 | 0 |
| | 5 | 0 | 0 |
| | 6 | 0 | 0 |
| | 7 | 0 | 0 |
| | 8 | 0 | 0 |
| 661W 0 4 | 1 | 120.282 | 0 |
| | 2 | 0 | 0 |
| | 3 | 0 | 0 |
| | 4 | 0 | 0 |
| | 5 | 0 | 0 |
| | 6 | 0 | 0 |
| | 7 | 0 | 0 |
| | 8 | 0 | 0 |
| | 9 | 0 | 0 |
| 661W 0 5 | 1 | 0 | 0 |
| | 2 | 200.848 | 0 |
| | 3 | 0 | 0 |
| | 4 | 0 | 0 |
| | 5 | 0 | 0 |
| 661W 0 6 | 1 | 169.85 | 0 |
| | 2 | 0 | 0 |
| | 3 | 0 | 0 |
| | 4 | 0 | 0 |
| | 5 | 0 | 0 |
| | 6 | 0 | 0 |
| 661W 100 1 | 1 | 484.468 | 0 |
| | 2 | 0 | 0 |
| | 3 | 174.95 | 0 |
| 661W 100 2 | 1 | 1105.155 | 1 |
| | 2 | 314.795 | 0 |
| | 3 | 165.054 | 0 |
| 661W 100 3 | 1 | 221.192 | 0 |
| | 2 | 168.941 | 0 |
| | 3 | 0 | 0 |
| 661W 100 4 | 1 | 337.251 | 1 |
| | 2 | 174.81 | 0 |
| | 3 | 0 | 0 |
| | 4 | 225.972 | 0 |
| | 5 | 230.965 | 0 |
| | 6 | 0 | 0 |
| | 7 | 134.634 | 0 |
| | 8 | 216.842 | 0 |
| 661W 100 5 | 1 | 268.388 | 0 |
| | 2 | 545.086 | 1 |
| | 3 | 0 | 0 |
| | 4 | 145.8 | 0 |
| | 5 | 0 | 0 |
| | 6 | 783.596 | 2 |
| 661W 100 6 | 1 | 506.177 | 0 |
| | 2 | 85.075 | 0 |
| | 3 | 262.959 | 0 |
| 661W 316 1 | 1 | 150.921 | 4 |
| | 2 | 285.062 | 3 |
| | 3 | 1003.71 | 5 |
| 661W 316 2 | 1 | 387.162 | 4 |
| | 2 | 618.091 | 3 |
| | 3 | 172.43 | 5 |
| 661W 316 3 | 1 | 1017.483 | 6 |
| | 2 | 241.208 | 5 |

| | | | |
|---|---|---|---|
| | 3 | 449.356 | 2 |
| 661W 316 4 | 1 | 0 | 5 |
| | 2 | 241.348 | 3 |
| | 3 | 434.714 | 2 |
| | 4 | 339.855 | 2 |
| | 5 | 181.861 | 4 |
| | 6 | 468.395 | 1 |
| | 7 | 1012.294 | 3 |
| | 8 | 195.714 | 2 |
| | 9 | 378.156 | 2 |
| 661W 316 5 | 1 | 1173.747 | 6 |
| | 2 | 1782.041 | 3 |
| 661W 316 6 | 1 | 514.417 | 4 |
| | 2 | 768.096 | 2 |
| | 3 | 640.908 | 4 |
| | 4 | 396.696 | 5 |
| | 5 | 477.972 | 5 |
| 661W 1000 1 | 1 | 837.256 | 3 |
| | 2 | 1189.942 | 8 |
| | 3 | 534.636 | 1 |
| | 4 | 344.478 | 1 |
| 661W 1000 2 | 1 | 129.018 | 0 |
| | 2 | 475.877 | 3 |
| | 3 | 608.506 | 2 |
| 661W 1000 3 | 1 | 195.894 | 0 |
| 661W 1000 4 | 1 | 615.559 | 4 |
| | 2 | 109.993 | 0 |
| | 3 | 98.265 | 0 |
| | 4 | 85.539 | 0 |
| 661W 1000 5 | 1 | 85.194 | 0 |
| | 2 | 595.99 | 3 |
| | 3 | 111.961 | 0 |
| 661W 1000 6 | 1 | 701.754 | 2 |
| | | | |
| RGC5 0 1 | 1 | 0 | 0 |
| | 2 | 0 | 0 |
| | 3 | 77.166 | 0 |
| | 4 | 0 | 0 |
| | 5 | 0 | 0 |
| | 6 | 0 | 0 |
| | 7 | 0 | 0 |
| | 8 | 0 | 0 |
| | 9 | 0 | 0 |
| | 0 | 0 | 0 |
| | 10 | 0 | 0 |
| | 11 | 0 | 0 |
| | 12 | 0 | 0 |
| | 13 | 0 | 0 |
| | 14 | 0 | 0 |
| | 15 | 0 | 0 |
| | 16 | 146.597 | 0 |
| | 17 | 103.578 | 0 |
| | 18 | 0 | 0 |
| | 19 | 0 | 0 |
| | 20 | 0 | 0 |
| | 21 | 0 | 0 |
| | 22 | 0 | 0 |
| | 23 | 123.472 | 0 |
| RGC5 0 2 | 1 | 0 | 0 |
| | 2 | 0 | 0 |
| | 3 | 101.245 | 0 |
| | 4 | 87.76 | 0 |
| | 5 | 133.501 | 0 |
| | 6 | 0 | 0 |
| | 7 | 0 | 0 |
| | 8 | 0 | 0 |
| | 9 | 0 | 0 |
| | 10 | 0 | 0 |
| | 11 | 0 | 0 |
| | 12 | 0 | 0 |
| | 13 | 67.768 | 0 |
| | 14 | 0 | 0 |
| | 15 | 0 | 0 |
| | 16 | 0 | 0 |
| | 17 | 0 | 0 |
| | 18 | 0 | 0 |
| | 19 | 0 | 0 |
| | 21 | 0 | 0 |

| | | | |
|---|---|---|---|
| | 22 | 0 | 0 |
| RGC5 0 3 | 1 | 116.628 | 0 |
| | 2 | 99.412 | 0 |
| | 3 | 0 | 0 |
| | 4 | 97.032 | 0 |
| | 5 | 98.021 | 0 |
| | 6 | 0 | 0 |
| | 7 | 0 | 0 |
| | 8 | 0 | 0 |
| | 9 | 0 | 0 |
| | 10 | 162.64 | 0 |
| | 11 | 131.166 | 0 |
| RGC5 0 4 | 1 | 0 | 0 |
| | 2 | 0 | 0 |
| | 3 | 0 | 0 |
| | 4 | 0 | 0 |
| | 4 | 0 | 0 |
| | 5 | 0 | 0 |
| | 6 | 0 | 0 |
| | 7 | 0 | 0 |
| | 8 | 0 | 0 |
| | 9 | 0 | 0 |
| | 10 | 0 | 0 |
| | 11 | 162.836 | 0 |
| | 12 | 0 | 0 |
| | 13 | 0 | 0 |
| | 14 | 0 | 0 |
| | 15 | 0 | 0 |
| | 16 | 180.106 | 0 |
| | 17 | 0 | 0 |
| | 18 | 0 | 0 |
| | 19 | 124.636 | 0 |
| | 20 | 0 | 0 |
| | 21 | 0 | 0 |
| | 22 | 0 | 0 |
| | 23 | 152.201 | 0 |
| RGC5 0 5 | 1 | 0 | 0 |
| | 2 | 0 | 0 |
| | 3 | 0 | 0 |
| | 4 | 0 | 0 |
| | 5 | 0 | 0 |
| | 6 | 0 | 0 |
| | 7 | 0 | 0 |
| | 8 | 0 | 0 |
| | 9 | 0 | 0 |
| | 10 | 0 | 0 |
| | 11 | 0 | 0 |
| | 12 | 0 | 0 |
| | 13 | 129.661 | 0 |
| | 14 | 0 | 0 |
| | 15 | 0 | 0 |
| RGC5 0 6 | 1 | 0 | 0 |
| | 2 | 0 | 0 |
| | 3 | 0 | 0 |
| | 4 | 0 | 0 |
| | 5 | 272.647 | 0 |
| | 6 | 0 | 0 |
| | 7 | 0 | 0 |
| | 8 | 423.225 | 0 |
| | 9 | 0 | 0 |
| | 10 | 0 | 0 |
| | 11 | 0 | 0 |
| | 12 | 0 | 0 |
| | 13 | 0 | 0 |
| | 14 | 0 | 0 |
| | 15 | 0 | 0 |
| | 16 | 0 | 0 |
| | 17 | 0 | 0 |
| | 18 | 0 | 0 |
| | 19 | 0 | 0 |
| | 20 | 0 | 0 |
| | 21 | 0 | 0 |
| | 22 | 0 | 0 |
| | 23 | 0 | 0 |
| | 24 | 0 | 0 |
| RGC5 100 1 | 1 | 441.738 | 2 |
| | 2 | 0 | 0 |
| | 3 | 0 | 0 |

| | | | |
|---|---|---|---|
| | 4 | 192.49 | 0 |
| RGC5 100 2 | 1 | 747.175 | 2 |
| | 2 | 595.777 | 1 |
| | 3 | 369.496 | 0 |
| | 4 | 442.51 | 0 |
| RGC5 100 3 | 1 | 138.281 | 0 |
| | 2 | 804.395 | 2 |
| | 3 | 367.061 | 0 |
| | 4 | 487.69 | 1 |
| | 5 | 107.67 | 0 |
| | 6 | 449.547 | 0 |
| | 7 | 374.416 | 0 |
| RGC5 100 4 | 1 | 95.062 | 0 |
| | 2 | 122.391 | 0 |
| | 3 | 451.529 | 1 |
| | 4 | 197.755 | 0 |
| | 5 | 380.867 | 0 |
| RGC5 100 5 | 1 | 84.657 | 0 |
| | 2 | 351.642 | 1 |
| | 3 | 143.664 | 0 |
| | 4 | 107.794 | 0 |
| | 5 | 307.065 | 0 |
| | 6 | 0 | 0 |
| RGC5 100 6 | 1 | 124.609 | 0 |
| | 2 | 144.708 | 0 |
| | 3 | 0 | 0 |
| RGC5 316 1 | 1 | 546.459 | 4 |
| | 2 | 277.815 | 3 |
| RGC5 316 2 | 1 | 912.108 | 3 |
| | 2 | 64.095 | 5 |
| | 3 | 563.69 | 5 |
| RGC5 316 3 | 1 | 296.286 | 4 |
| | 2 | 324.436 | 3 |
| RGC5 316 4 | 1 | 1004.495 | 5 |
| | 2 | 749.738 | 4 |
| RGC5 316 5 | 1 | 580.463 | 3 |
| | 2 | 63.959 | 2 |
| | 3 | 182.983 | 4 |
| | 4 | 257.879 | 3 |
| | 5 | 562.356 | 4 |
| RGC5 316 6 | 1 | 588.205 | 5 |
| | 2 | 519.906 | 3 |
| | 3 | 441.393 | 5 |
| RGC5 1000 1 | 1 | 582.875 | 3 |
| | 2 | 311.189 | 1 |
| | 3 | 417.231 | 2 |
| | 4 | 558.932 | 1 |
| | 5 | 1007.62 | 2 |
| | 6 | 381.289 | 0 |
| RGC5 1000 2 | 1 | 988.764 | 1 |
| | 2 | 754.447 | 3 |
| | 3 | 673.861 | 2 |
| | 4 | 336.79 | 1 |
| RGC5 1000 3 | 1 | 321.31 | 1 |
| | 2 | 231.688 | 0 |
| | 3 | 282.786 | 1 |
| | 4 | 195.515 | 1 |
| | 5 | 211.356 | 2 |
| RGC5 1000 4 | 1 | 148.946 | 0 |
| | 2 | 392.106 | 1 |
| | 3 | 0 | 1 |
| | 4 | 121.601 | 3 |
| RGC5 1000 5 | 1 | 762.312 | 2 |
| | 2 | 351.28 | 1 |
| | 3 | 248.714 | 1 |
| | 4 | 211.832 | 1 |
| | 5 | 358.912 | 0 |
| RGC5 1000 6 | 1 | 236.498 | 1 |
| | 2 | 411.149 | 2 |
| | 3 | 115.913 | 1 |

| Cell Type | [SS] | Avg. Total Neurite Field (pixels) | SEM Field | Neurite Field (microns) | SEM Microns |
|---|---|---|---|---|---|
| 661W | 0 | 27.04018605 | 9.964172942 | 7.135178522 | 2.623048133 |
| | 100 nM | 252.0042308 | 50.99342063 | 66.4971451 | 13.42391361 |
| | 316 nM | 533.26548 | 80.46719426 | 140.7144312 | 21.18282419 |
| | 1 µM | 419.991375 | 82.31988228 | 110.8244386 | 21.67054052 |
| | | | | 0 | 0 |
| RGC-5 | 0 | 25.13695798 | 5.031467617 | 6.632967777 | 1.324523552 |
| | 100 nM | 276.8961724 | 40.81665871 | 73.06545964 | 10.74490186 |
| | 316 nM | 466.8391765 | 65.22493689 | 123.1863146 | 17.17033114 |
| | 1 µM | 393.145037 | 49.41088614 | 103.7404114 | 13.00731465 |

| Cell Type | [SS] | Avg. Branch Points | SEM Points |
|---|---|---|---|
| 661W | 0 | 0 | 0 |
| | 100 nM | 0.192307692 | 0.096384339 |
| | 316 nM | 3.6 | 0.282842712 |
| | 1 µM | 1.6875 | 0.545578821 |
| | | | |
| RGC-5 | 0 | 0 | 0 |
| | 100 nM | 0.344827586 | 0.124329354 |
| | 316 nM | 3.823529412 | 0.230652752 |
| | 1 µM | 1.296296296 | 0.167219722 |

ttest:

| RGC-5 v. 661W Neurite Field | |
|---|---|
| 0 v. 0 | 0.869353993 |
| 100 v. 100 | 0.704772971 |
| 316 v. 316 | 0.524991525 |
| 1000 v. 1000 | 0.781997454 |

| RGC-5 v. 661W Branch Points | |
|---|---|
| 0 v 0 | #DIV/0! |
| 100 v 100 | 0.336850478 |
| 316 v 316 | 0.543696376 |
| 1000 v 1000 | 0.501790477 |

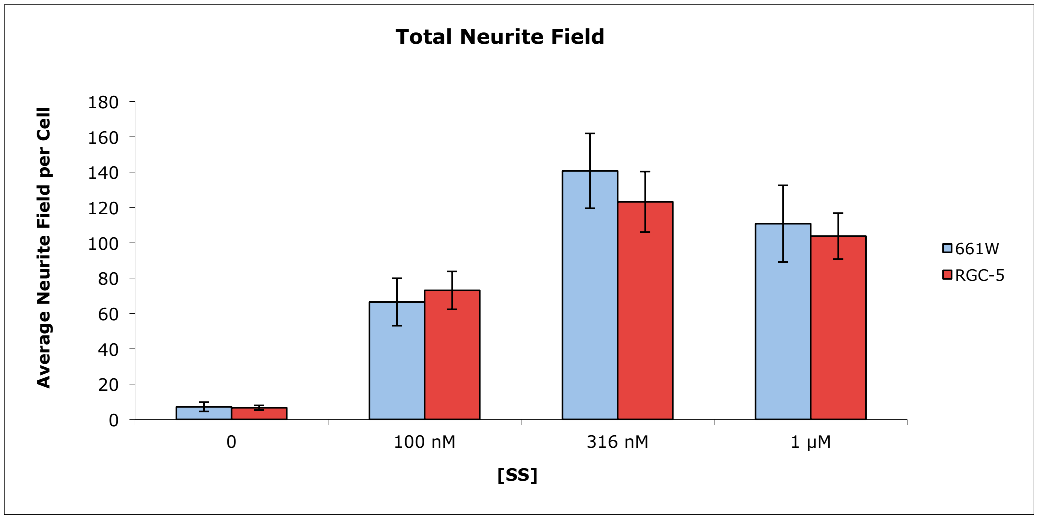
Total Neurite Field
Average Neurite Field per Cell
180
160
140
120
100
80
60
40
20
0
0
100 nM
316 nM
1 µM
[SS]
661W
RGC-5

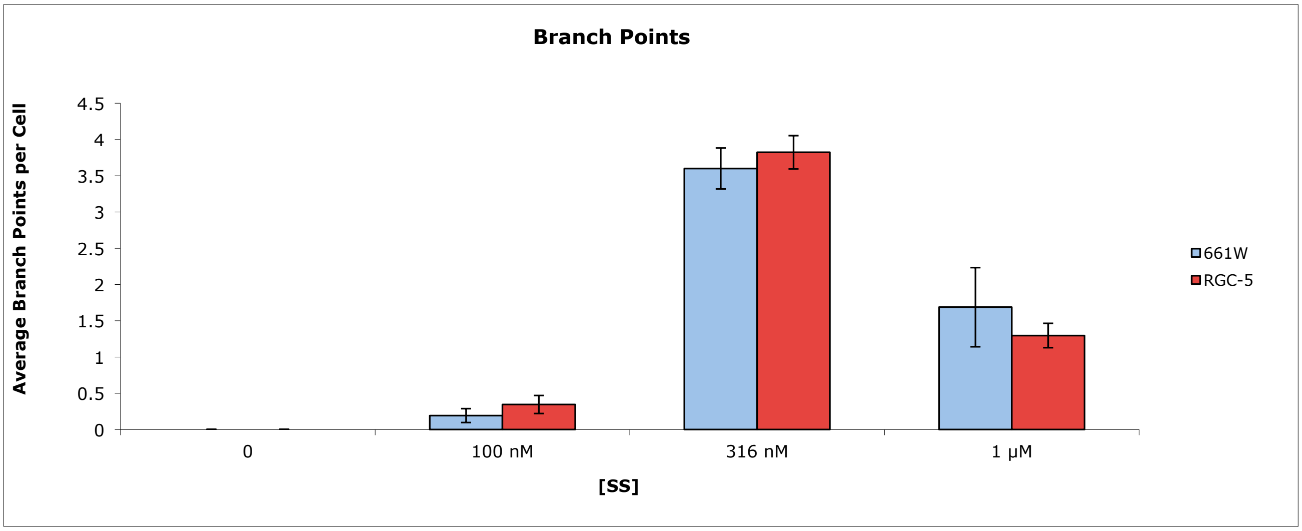
Branch Points
Average Branch Points per Cell
4.5
4
3.5
3
2.5
2
1.5
1
0.5
0
0
100 nM
316 nM
1 µM
[SS]
661W
RGC-5
